# *PLIN5* Promotes Lipid Reconstitution in Goat Intramuscular Fat via the PPARγ Signaling Pathway

**DOI:** 10.3390/biology14111547

**Published:** 2025-11-04

**Authors:** Yuhan Dai, Yuling Yang, Haiyang Li, Yinggui Wang, Yong Wang, Yaqiu Lin, Lian Huang, Zhanyu Du, Hua Xiang, Changhui Zhang, Jiangjiang Zhu

**Affiliations:** 1 Qinghai-Tibetan Plateau Animal Genetic Resource Reservation and Utilization Key Laboratory of Sichuan Province, Southwest Minzu University, Chengdu 610041, China; dyh20010228@163.com (Y.D.); yulingyang1012@163.com (Y.Y.); haiyang6715@163.com (H.L.); yingguiwang326@163.com (Y.W.); wangyong010101@hotmail.com (Y.W.); linyq1999@163.com (Y.L.); kin8248806@163.com (L.H.); yuzhan.du@outlook.com (Z.D.); xianghua2008411@163.com (H.X.); 2 Key Laboratory of Qinghai-Tibetan Plateau Animal Genetic Resource Reservation and Utilization, Southwest Minzu University, Ministry of Education, Chengdu 610041, China

**Keywords:** *PLIN5*, *PPARγ*, PI3K-AKT pathway, IMF, intramuscular adipocyte

## Abstract

**Simple Summary:**

Goat muscle intramuscular fat (IMF) is associated with the proliferation and differentiation of intramuscular precursor adipocytes, and IMF has various effects on meat quality. *PLIN5* is a lipid droplet protein that plays an important role in lipid metabolism. However, the specific function and intrinsic mechanism of *PLIN5* in goat IMF deposition remain unclear. Therefore, in this study, we clarified the function and potential mechanism of *PLIN5* in goat intramuscular precursor adipocytes via experimental techniques such as overexpression, interference, untargeted lipid omics sequencing, inhibitor addition, and so on. It was found that *PLIN5* regulates intramuscular lipid deposition in goat muscle via PPARγ and regulates intramuscular cell proliferation via the PI3K-AKT signaling pathway, thereby altering IMF. Untargeted lipidomic sequencing indicated that the differential lipids LPI (18:0) and LPI (16:0) may be involved in the activation of the PPARγ signaling pathway. These findings elucidate the potential regulatory mechanism of *PLIN5* on IMF deposition and provide a theoretical basis for improving meat quality by targeting and regulating IMF deposition, which is important in breeding to improve goat meat quality.

**Abstract:**

Intramuscular fat (IMF) content is an important factor of goat meat quality, which is related to the proliferation and differentiation of intramuscular preadipocytes. Perilipin 5 (PLIN5) is a lipid droplet-associated protein; however, the specific function and underlying mechanism of *PLIN5* in goat IMF deposition are still unclear. In this study, overexpression of *PLIN5* significantly enhanced apoptosis and reduced the proliferation of preadipocytes and also promoted cellular lipid deposition via both the upregulation of the expression of peroxisome proliferator-activated receptor gamma (*PPARγ*) and a significant increase in the expression of lipogenesis-related genes. The inhibition of *PLIN5* then confirmed these results. Untargeted lipidomics sequencing identified a total of 34 differentially expressed lipids after *PLIN5* overexpression in goat preadipocytes and analysis by KEGG pathway enrichment, which are mainly involved in the PI3K-AKT signaling pathway. The lipid omics findings also show that ceramides and lysophosphatidylinositol were significantly upregulated, e.g., Cer (d35:1), Cer (d18:2/22:1), LPI (18:0), and LPI (16:0), after overexpression of the *PLIN5* gene. Higher expression of LPI (16:0) or LPI (18:0) may regulate lipid droplet accumulation by activating *PPARγ*. Rescue experiments with the PI3K-AKT inhibitor (LY294002) and the PPARγ inhibitor (GW9662) showed that the PI3K-AKT signaling pathway is involved in the regulation of cell proliferation, and PPARγ is involved in the regulation of lipid deposition. In conclusion, our findings demonstrate that *PLIN5* regulates lipid reconstitution in goat intramuscular fat via PPARγ and PI3K-AKT signaling pathways. This regulation delivered theoretical support for improving meat quality from the aspect of IMF deposition.

## 1. Introduction

Goat meat has the advantage of being high in protein and low in cholesterol, and is in line with the healthy eating habits of modern society. It is generally assumed that intramuscular fat (IMF) content positively influences sensory quality traits, including flavor, juiciness, and tenderness of goat meat [1]. IMF content is affected by adipocyte size and the number of intramuscular preadipocytes, which are determined by the differentiation and proliferation, respectively [2,3]. Multiple lipid metabolism genes are involved in the complex process of muscular fat deposition. For example, the upregulation of forkhead box 6 (*FOXO6*) inhibited proliferation and promoted apoptosis in chicken preadipocytes [4]. Liver kinase B1 (*LKB1*) [5], cell death-inducing DFFA-like effector B (*CIDEB*) [6], and Acyl-CoA dehydrogenase (*ACADS*) [7] were also demonstrated to critically regulate both adipogenic differentiation and lipid metabolic processes during preadipocyte development. Therefore, screening for key genes and elucidating the underlying molecular mechanisms of intramuscular fat deposition in Capra hircus are crucial for enhancing goat meat quality.

PLIN5 (myocardial lipid droplet protein, perilipin 5, also known as MLDP, LSDPS, LSDA5, or OXPAT) is a key member of the perilipin (PLIN) protein family (PLIN1-5) and is highly expressed in cardiac, hepatic, brown adipose tissue (BAT), and skeletal muscle [8,9,10]. *PLIN5* localizes to multiple cellular compartments, including lipid droplets, the endoplasmic reticulum, mitochondria, and cytoplasm [11], orchestrating dynamic interorganelle communication between LDs and mitochondria [12,13]. It plays a pivotal role in regulating lipogenic processes. Expression of *PLIN5* in cells promotes both triacylglycerol (TAG) storage and fatty acid oxidation and coats lipid droplets during rapid TAG synthesis [9]. *PLIN5*-deficient murine accelerated intramyocellular lipolysis concomitant with depleted muscular lipid reserves under fasting conditions [14]. In ruminants, PLIN5 is associated with meat quality traits in cattle [15] and lipolysis in adipose tissue of periparturient dairy cows [16].

Furthermore, *PLIN5* is also involved in cellular proliferation and apoptosis regulation. Demethylated PLIN5 inhibits cell proliferation and increases apoptosis [17]. Expression of *PLIN5* significantly inhibited apoptosis, oxidative stress, and pro-inflammatory response induced by glucose deprivation/re-oxidation (OGD/R) [18] in primary neuronal cells. However, the role of *PLIN5* in goat intramuscular fat deposition remains unclear.

The PI3K/AKT signaling axis and *PPARγ* (peroxisome proliferator-activated receptor γ) gene are essential for basic life processes such as cell proliferation and differentiation. Evidence demonstrates that PI3K/AKT is a key pathway regulating bovine intramuscular adipocyte differentiation [19] and affecting the proliferation of porcine intramuscular preadipocytes [20]. PPARγ, one of the most studied members within the peroxisome proliferator-activated receptor (PPAR) family, plays a crucial role in the control of adipocyte differentiation and adipose tissue formation in vivo [21]. *PPARγ* may begin the adipogenic process and is important for maintaining the adipocyte phenotype with an activating ligand in mammals [22]. In nonruminants, most long-chain fatty acids (LCFAs), and specifically polyunsaturated fatty acids (PUFAs), are natural ligands and bind to *PPARγ*, eliciting changes in gene expression and rates of lipogenesis [23]. It was shown that palmitate (16:0) and stearate (18:0) are *PPARγ* ligands. Specifically, 16:0 enhances PPARγ activation-induced fatty acid synthesis and esterification reactions while increasing triglycerides (TAG) in cows; in contrast to palmate, 18:0 decreased mammary gland fatty acid synthesis de novo in epithelial cells but increased TAG synthesis [24]. Therefore, we hypothesize that the PI3K/AKT signaling axis and PPARγ critically regulate lipid homeostasis within caprine intramuscular preadipocytes.

This study employed RNA interference and overexpression to investigate the role of *PLIN5* in intramuscular preadipocytes. *PLIN5* enhances adipogenesis and apoptotic processes while suppressing proliferation in goat intramuscular preadipocytes. Furthermore, lipid omics analysis was performed to identify differentially abundant lipid species associated with *PLIN5* overexpression. Through Western blot analyses and rescue experiments, we demonstrated that *PLIN5* mediates lipid deposition in goat adipocytes by positively regulating *PPARγ*, while concurrently modulating lipid shunt metabolism. Importantly, under conditions of *PPARγ* deficiency resulting from *PLIN5* downregulation, the PI3K-AKT pathway is activated to promote cellular proliferation. Modulation of *PLIN5* expression regulated lipid partitioning between deposition and proliferation pathways in caprine intramuscular adipocytes, widened the regulatory network of IMF formation, and established a theoretical foundation for meat quality improvement via targeted regulation of IMF deposition.

## 2. Materials and Methods

### 2.1. Ethics Statement

All experimental exercises were reviewed and approved by the Institutional Animal Care and Use Committee, Southwest Minzu University (Chengdu, China). Permit number: S2020-013.

### 2.2. Cell Isolation and Culture

Intramuscular preadipocytes were isolated from the longissimus dorsi muscle of three male Jianzhou goats (randomly selected three unrelated two-day-old goats). Cell isolation and culture procedures were performed following a previously established protocol developed by our laboratory [25]. Briefly, the dorsal longissimus muscle tissue was aseptically collected from goats, rinsed twice with phosphate-buffered saline (PBS) containing 1% penicillin/streptomycin (*v*/*v*), then minced using surgical scissors and homogenized uniformly. The tissue was digested with double-volume collagenase type II (Sigma-Aldrich, C2-BIOC, St. Louis, MO, USA) in a 37 °C water bath for 1.5 h. The enzymatic reaction was then terminated by adding an equal volume of DMEM/F12 medium (Gibco, Carlsbad, CA, USA) supplemented with 10% fetal bovine serum (FBS). The suspension was sequentially filtered through sterile gauze and a 75 μM cell filter, followed by centrifugation at 2000 rpm for 5 min. The pellet was resuspended in erythrocyte lysis buffer (BOSTER, AR1118, Wuhan, China), with subsequent centrifugation at 2000 rpm for 5 min again to remove supernatant. Resuspended cells in DMEM/F12 medium (Gibco, Carlsbad, CA, USA) supplemented with 10% FBS were plated in 25 cm^2^ culture flasks and maintained at 37 °C with 5% CO_2_. Following a 2 h incubation, the initial medium containing non-adherent cells (e.g., muscle cells) was discarded and replaced with fresh medium, with subsequent medium changes performed every 48 h. Cells were passaged at a 1:3 ratio and cultured to the third generation before being seeded into 10 cm^2^ culture plates. For adipogenic differentiation, the growth medium was replaced with adipocyte induction medium (MEM/F12 supplemented with 10% FBS, 1% antibiotic/antimycotic solution, and 50 μmol L−1oleic acid). The goat intramuscular preadipocytes reached 70–80% confluence. DMEM-F12 (Gibco, Carlsbad, CA, USA) containing 10% FBS (Gibco, Carlsbad, CA, USA), 1‰ Penicillin-Streptomycin, and 50 µM oleic acid (Sigma, St Louis, MO, USA) was used to induce adipogenic differentiation to goat intramuscular adipocytes [26,27].

### 2.3. PLIN5 Gene Cloning and Biological Analysis

Total RNA was extracted from various goat tissues (heart, liver, spleen, lungs, kidneys, longissimus dorsi, biceps femoris) using the Trizol method. Subsequently, cDNA was synthesized following the manufacturer’s protocol provided with the reverse transcription kit. The cloning primers were designed according to the predicted caprine *PLIN5* mRNA sequence (GenBank accession: XM_018050879.1). The coding sequence was amplified using cardiac tissue cDNA as template through TA cloning to obtain the *PLIN5* gene sequence (GenBank: PP824817.1). The specific primers and annealing temperature are listed in Supplementary Table S1. Bioinformatic analysis was performed using various online software (Supplementary Table S2).

### 2.4. Construction of pc DNA3.1-PLIN5 Overexpression Vector and siRNA Synthesis

The pcDNA3.1(+) vector and *PLIN5* gene were digested with EcoRI and HindIII restriction enzymes, respectively. Then linearized fragments were ligated using T4 DNA ligase and transformed into DH5α competent cells (Trelief^TM^ 5α Chemically Competent Cell, DLC101, Chengdu, China). Positive clones were screened and verified by sequencing, named pcDNA3.1-*PLIN5* plasmid. (Supplementary Material Figure S1). The pcDNA3.1(+) empty vector served as the negative control for subsequent experimental groups. si-*PLIN5* (CUGAUCAGGGCCACGUGCATT), si-*PPARγ* (CCCGAUGGUUGCAGAUUAUTT), and Negative control si-NC (UUCUCCGAACGUGUCACGUTT) were provided by Shanghai GenePharma Co., Ltd. (Shanghai, China).

### 2.5. Cell Transfection

Transfection was initiated when intramuscular preadipocytes cultured in 6-well plates reached 80% confluency. According to the manufacturer’s instructions, 1 μg plasmid per well in 6-well plates was transfected with Lipofectamine™ 3000 (Thermo Fisher Scientific, Waltham, MA, USA). For siRNA transfection, 20 μM siRNA per well was transfected with Lipofectamine™ RNAiMAX (Thermo Fisher Scientific, Waltham, MA, USA). Briefly, 900 μL was added to each well of a six-well plate to starve the cells by treating them with reduced serum medium (Opti-MEM^®^I(1X), Gibco, Carlsbad, CA, USA) for 4 h. Then, prepare a 100 μL transfection mixture by combining Lipofectamine™ 3000, p3000, plasmid, and Opti-MEM^®^I (or Lipofectamine™ RNAiMAX, siRNA, and Opti-MEM^®^I). The final concentration of siRNA is 20 nM. Gently mix the components by vortexing, then allow the mixture to stand at room temperature for 10 to 15 min before slowly adding it to the starved cells. Incubate for 6 h. Aspirate 1 mL of liquid from each well. After PBS washing 3–5 times, added 2 mL to each well of a six-well plate with 50 μM oleic acid induction medium. Collect samples after 48 h for subsequent experiments.

### 2.6. Oil Red O, Bodipy Staining, and Triglyceride Content Determination

Following previous laboratory protocols, cells were washed thrice with PBS after treatment, fixed with 4% formaldehyde for 30 min, and then washed three times with PBS to remove residual fixative, and freshly Oil Red O dye solution was added (Solarbio, G1262, Beijing, China, 3 mL Oil Red 5 g/L dissolved in isopropyl alcohol and 2 mL ddH_2_O mixture). Following staining for 30 min, the cells were cleaned with PBS, and images were taken under a microscope. Then, 600 μL of isopropanol was added to each well of the 6-well plate. Lipid content was quantified at 510 nm using a microplate reader (Thermo Fisher 1510, Waltham, MA, USA) and data corrected using BCA. Similarly, following formaldehyde fixation, cells were stained with 300 µL BODIPYTM 493/503 working solution (1 μg/mL, Thermo Fisher Scientific, D3922, Waltham, MA, USA) and incubated on a shaker at room temperature for 30 min. After three PBS washes, nuclei were stained with 300 μL DAPI solution (1 μg/mL, Solarbio, C0060, Beijing, China) for 10 min. After PBS washing three times, a fluorescence microscope was used to take photos.

Total triglyceride (TG) content was extracted using a triglyceride assay kit (Applygen, E1013, Beijing, China). Following cell lysis, supernatants were collected by centrifugation at 2000× *g* for 2 min. TG levels were absorbance value at a wavelength of 550 nm by a microplate reader. At the same time, a BCA protein quantification kit (Thermo Fisher Scientific, 23225, Waltham, MA, USA) was used for protein quantification; the absorbance value was measured at a wavelength of 562 nm, and corrected for triglycerides.

### 2.7. CCK-8 Assay and EDU Staining

Intramuscular adipocyte proliferation was quantified using the CCK-8 Cell Proliferation Assay Kit (Life-ilab, Shanghai, China) according to the manufacturer’s protocol. Cells were seeded into 96-well plates, and pcDNA 3.1-*PLIN5*, Si-*PLIN5*, si-*PPARγ,* and the corresponding negative controls were transfected into the cells with a transfection reagent. Similarly, cells were seeded into 96-well plates, LY294002 (Beyotime, S1737-1 mg, 5 μΜ) and the corresponding DMSO as controls were added into the cells. At 0 h, 12 h, 24 h, 36 h, and 48 h post-transfection, 10 μL CCK-8 reagent was added per well after 30 min incubation at 37 °C, and absorbance was measured at 450 nm using a microplate reader. BeyoClick™ EDU-488 Cell Proliferation assay kit (Beyotime Biotechnology, C0071S, Shanghai, China) was used for EDU staining, following all procedures as recommended by the manufacturer.

### 2.8. Analysis by Flow Cytometry

Cells were seeded into six-well plates, which were digested using DETA-free trypsin and subsequently collected. Cell cycle distribution and apoptotic rates were analyzed by flow cytometry using Propidium iodide (Solarbio, C0080, Beijing, China) and an Annexin V-FITC/PI Apoptosis Detection Kit (Vazyme, A211, Nanjing, China) according to the manufacturers’ protocols.

### 2.9. Total RNA Isolation, cDNA Synthesis, and q-PCR Analysis

Total cellular RNA was extracted using RNAiso Plus (Takara, 9109, Kusatsu, Japan). Subsequently, reverse-transcribed into cDNA with the ExonGen Reverse Transcription Kit A502-02, Chengdu, China), utilizing 1 μg of RNA as template. Real-time fluorescence quantitative PCR was performed using Taq Pro Universal SYBR qPCR Master Mix (ExonGen, A411-01, Chengdu, China) and a Bio-Rad CFX96 PCR system. UXT was used as an internal reference gene, and the relative expression was calculated using the 2^−∆∆CT^ method. The related primer sequences are shown in Supplementary Table S3.

### 2.10. Western Blot

Cells were seeded into 6-well plates, and pcDNA 3.1-*PLIN5*, Si-*PLIN5*, si-*PPARγ*, and the corresponding negative controls were transfected into the cells with a transfection reagent. Similarly, cells were seeded into 6-well plates, LY294002 (Beyotime, S1737-1 mg, 5 μΜ, ShangHai, China), GW9662 (Beyotime, SC9123-25 mg, 15 μΜ, ShangHai, China), and the corresponding DMSO as controls were added into the cells. Collect protein samples after 48 h. Following collection, cells were lysed using RIPA (Beyotime; P0013, ShangHai, China) lysis buffer supplemented with protease/phosphatase inhibitor for Western blotting. Protein concentrations were quantified using the BCA Protein Assay Kit (Thermo Fisher Scientific, 23225, Waltham, MA, USA). The protein was separated by a 10% SDS-PAGE electrophoresis system and transferred to a PVDF membrane. Polyclonal rabbit anti-PLIN5 (Abclonal, A25598, 1:1000 dilution, Wuhan, China), PPARγ (Proteintech, 16643-1-Ap, 1:8000 dilution, Wuhan, China), Casepase-3 (Wanleibio, WL02117, 1:1000 dilution, Shenyang, China), AKT1 (Abcam, ab32505, 1:1000 dilution, Cambridge, ENG, UK), p-AKT1 Ser473 (Cell Signaling Technology, 4060, 1:2000 dilution, Danvers, MA, USA), p38-mapk (Cell Signaling Technology, 4, 1:1000 dilution, Danvers, MA, USA), p-p38-mapk (Cell Signaling Technology, 3285S, 1:1000 dilution, Danvers, MA, USA) and monoclonal mouse anti-β-actin monoclonal (Boster, BMo627, 1:8000 dilution, ChengDu, China) were used as the primary antibodies. Band densities were normalized to β-actin for all Western Blot experiments. Goat anti-mouse IgG coupled to HRP (Boster, BA1050, 1:10,000 dilution, ChengDu, China) and Goat anti-rabbit IgG coupled to HRP (Boster, BA1054, 1:10,000 dilution, ChengDu, China) were used as secondary antibodies. Signals were detected using the chemiluminescent ECL Western blot detection system (Oriscience, Chengdu, China). Image J (1.51j8, NIH, Bethesda, MD, USA) was used to calculate the protein gray value. All WB raw images can be found in Supplementary Materials Figures S7 and S8.

### 2.11. Untargeted Lipidomics Sequencing

Goat intramuscular preadipocytes were prepared in twelve 100 mm dishes. After medium removal, cells were washed twice with ice-cold PBS and digested with 0.5 mL trypsin per dish. The digestion was terminated with an equal volume of DMEM/F-12 (Gibco, Beijing, China) supplemented with 10% fetal bovine serum (FBS). Following three additional PBS washes, cells were pelleted by centrifugation at 1000× *g* for 5 min at 4 °C and collected in 1.5 mL tubes. Methanol (0.75 mL) was added to a 100 μL sample, which was placed into a glass tube with a Teflon-lined cap, and the tube was vortexed. 2.5 mL of MTBE was added, and the mixture was incubated for 1 h at room temperature on a shaker. Phase separation was induced by adding 0.625 mL of MS-grade water. After 10 min of incubation at room temperature, the sample was centrifuged at 1000× *g* for 10 min. The upper (organic) phase was collected, and the lower phase was re-extracted with 1 mL of the solvent mixture (MTBE/methanol/water (10:3:2.5, *v*/*v*/*v*)). And collecting the upper phase. Combined organic phases were dried and dissolved in 100 μL of isopropanol for storage. Then analyzed by LC-MS/MS.

### 2.12. In Silico Docking Analysis

Lipid molecules LPI (16:0) and LPI (18:0) were retrieved from PubChem in SMILES format. The PPARγ protein structure was predicted using AlphaFold. Molecular docking was performed using Autodock Vina within SwissDock.

### 2.13. Statistical Analysis

All quantitative data were expressed as means ± SEM. GraphPad Prism software (8.3.0, GraphPad software, La Jolla, CA, USA) and SPSS 19.0 were used for statistical calculations. The RT-qPCR data were analyzed using transcript Ct values normalized with UXT as the endogenous control gene (ΔCt). Student’s *t*-test and one-way ANOVA followed by Tukey’s post hoc test (for normally distributed data) or Dunnett’s T3 test (when comparing multiple groups to a single control), LSD, and Duncan were used for statistical analysis between the two groups and multiple groups. The difference was statistically significant when “*” *p* < 0.05, “**” *p* < 0.01.

## 3. Results

### 3.1. PLIN5 Is Associated with Intramuscular Fat Deposition

To study the function of *PLIN5*, we cloned the *PLIN5* mRNA sequence from the heart tissue of Jianzhou goat (Figure S1A, GenBank: PP824817.1). The full-length of goat *PLIN5* is 1984 bp, including 147 bp of 5′UTR, 1370 bp of CDS, and 467 bp of 3′UTR, and encodes a 456 amino acid polypeptide (Figure S1B). Using cardiac muscle tissue as a control, we found that the expression level of *PLIN5* was particularly prominent in goat liver and the longest dorsal muscle, followed by the biceps femoris, with low expression in the spleen, kidney, and lung (Figure S1C). Additional bioinformatics analyses are shown in the Supplementary (Figure S2).

**Figure 1:**
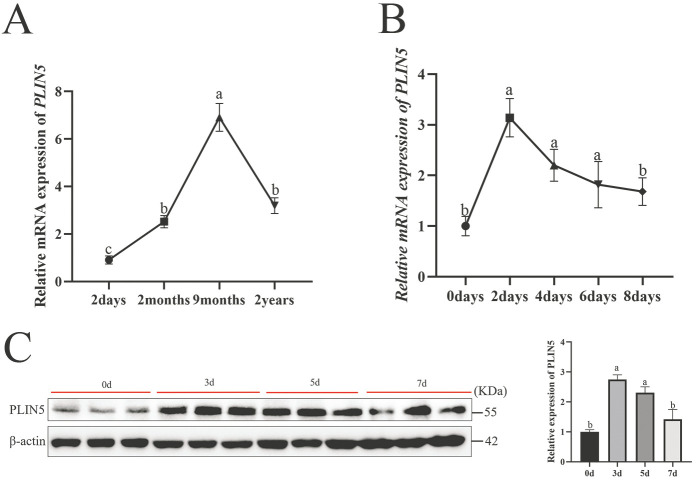
*PLIN5* is associated with intramuscular fat deposition. (**A**) *PLIN5* mRNA expression levels (*n* = 3) in the longest dorsal muscles of goats at different developmental stages (2 days, 2 months, 9 months, and 24 months). (**B**) Expression pattern of *PLIN5* during differentiation of goat preadipocytes (mRNA expression levels, *n* = 3). (**C**) Expression pattern of PLIN5 during differentiation of goat preadipocytes (protein expression levels, *n* = 3). Different lowercase letters indicate statistically significant differences (*p* < 0.05).

To investigate the role of *PLIN5* in intramuscular lipid deposition, we first analyzed *PLIN5* mRNA expression patterns in the goat longissimus dorsi muscle across developmental stages (2 days to 24 months). The expression of *PLIN5* gradually increased, then decreased, and the highest expression was reached in 9 months (Figure 1A). Secondly, the expression of PLIN5 in goat intramuscular preadipocytes during differentiation was detected. The mRNA levels from day 0 to day 8, which peaked at 2 days and reached the lowest point at 8 days, and protein levels from day 0 to day 7, which peaked at 3 days and reached the lowest point at 7 days (Figure 1B,C). These results predict that PLIN5 plays a key role in the adipogenesis of intramuscular preadipocytes.

### 3.2. Overexpression of PLIN5 Promotes Adipogenesis and Inhibits Proliferation of Goat Intramuscular Preadipocytes

**Figure 2:**
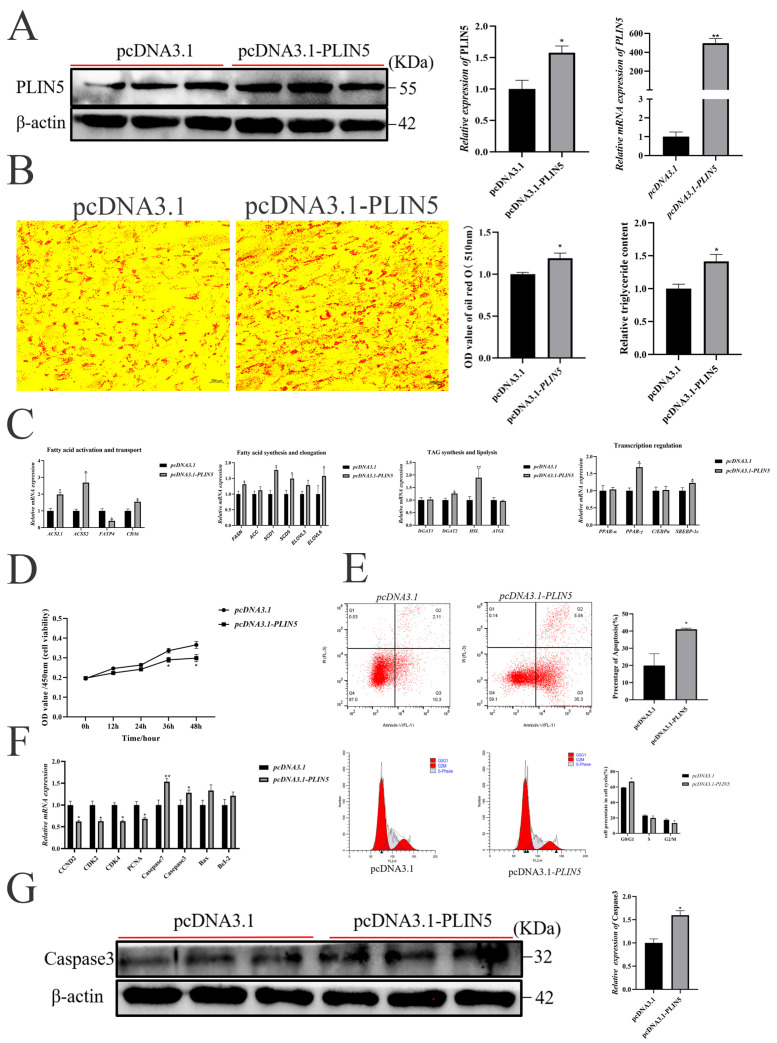
Overexpression of *PLIN5* Promotes Adipogenesis and Inhibits Proliferation of Goat Intramuscular Preadipocytes. (**A**) *PLIN5* overexpression efficiency detection (*n* = 3). UXT was used as an internal reference gene. (**B**) Oil red O staining (200×) and triglyceride content determination after overexpression of *PLIN5* (day 2 after adipogenic differentiation, *n* = 3). (**C**) Relative expression of lipid metabolism-related genes after overexpression of *PLIN5* (day 2 after adipogenic differentiation, *n* = 3). (**D**) Cell viability assay after *PLIN5* overexpression. (**E**) Effects on apoptosis and cell cycle of goat adipocytes after PLIN5 overexpression (*n* = 3). (**F**) Relative expression of proliferation-apoptosis related genes after overexpression of *PLIN5* (*n* = 3). (**G**) Relative protein expression of Caspase3 in cells after transfection with pcDNA3.1-*PLIN5* or pcDNA3.1 (*n* = 3). Data are expressed as mean ± SEM. “*” *p* < 0.05, “**” *p* < 0.01.

To verify the function of *PLIN5* in intramuscular preadipocyte adipogenesis, we transfected goat primary intramuscular preadipocytes with a subcloned vector pcDNA3.1-*PLIN5*. The results showed that *PLIN5* mRNA expression level and WB expression level were significantly increased after overexpression compared with the control group (Figure 2A). Oil red O staining (Figure 2B) and Bodipy staining (Figure S3A) showed a significant increase in lipid droplet content after overexpression of PLIN5. At the same time, the triglyceride (TAG) content was detected after *PLIN5* overexpression, and the results showed that the triglyceride content was significantly increased (Figure 2B). In addition, we also detected the expression of lipid metabolism-related genes (Figure 2C). The RT-qPCR results demonstrated that the overexpression of *PLIN5* significantly upregulated the expression of acyl-CoA synthetase long chain family member 1 (*ACSL1*), acyl-CoA synthetase short chain family member 2 (*ACSS2*), scavenger receptor B2 (*CD36*), fatty acid synthesis gene (*FASN*), the fatty acid desaturase gene (*SCD1* and *SCD5*), elongase of very long chain fatty acids 6 (*ELOVL6*), diacylglycerol o-acyltransferase 2 (*DGAT2*), hormone-sensitive triglyceride lipase (*HSL*), peroxisome proliferator-activated receptor gamma (*PPARγ*) and sterol regulatory element-binding protein 1c (*SREBP-1c*). But the expression of fatty acid transport protein 4 (*FATP4*) was significantly downregulated. There was no significant effect on the expression of the Acetyl-CoA carboxylase (*ACC*), very long chain fatty acids elongase 3 (*ELOVL3*), diacylglycerol o-acyltransferase 1 (*DGAT1*), adipose triglyceride lipase (*ATGL*), peroxisome proliferator-activated receptor alpha (*PPARα*), and enhancer-binding protein alpha (*C*/*EBPα*).

Next, the *PLIN5* overexpression significantly inhibited cell proliferation compared with the control group by CCK-8 determination (36 h and 48 h, Figure 2D). Flow cytometry experiments showed a significant increase in apoptosis (Figure 2E). In addition, cell cycle analysis showed that overexpression of *PLIN5* increased the proportion of cells in G0/G1 phase and decreased the proportion of cells in S phase and G2/M phase (Figure 2E). In addition, the overexpression of *PLIN5* significantly decreased the mRNA levels of cyclin D2 (*CCND2*), cyclin dependent kinase 2 (*CDK2*), cyclin dependent kinase 4 (*CDK4*) and proliferating cell nuclear antigen (*PCNA*); increased the expression of *Casepase-7* and *Casepase-3*; and had no significant effect on BCL2 associated X (*Bax*) and cell lymphoma-2 (*BcL2*) expressions (Figure 2F). Correspondingly, the WB results show that Caspase-3 protein expression increased after overexpression of *PLIN5* (Figure 2G).

### 3.3. Knockdown of PLIN5 Inhibits Adipogenesis and Promotes Proliferation of Goat Intramuscular Preadipocytes

**Figure 3:**
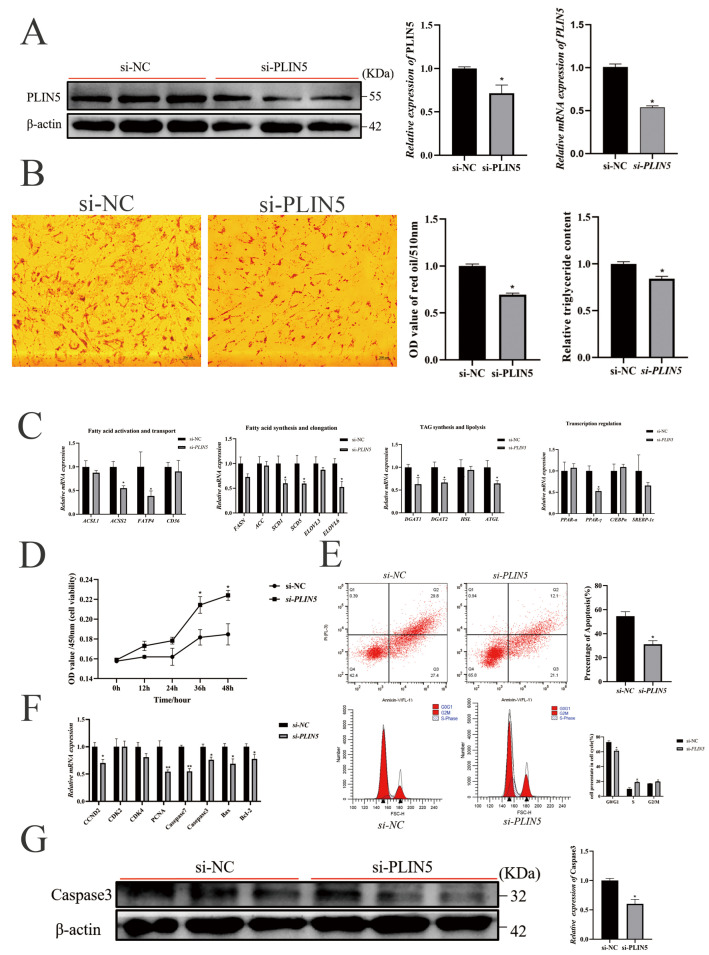
Knockdown of *PLIN5* promotes proliferation and inhibits apoptosis in goat intramuscular precursor adipocytes. (**A**) PLIN5 interference efficiency detection (*n* = 3). UXT was used as an internal reference gene. (**B**) Oil red O staining (200 times) and triglyceride content determination (2 days after adipogenic differentiation, *n* = 3) after interference with *PLIN5*. (**C**) The relative expression of lipid metabolism-related genes after knockdown of *PLIN5* (2 days after adipogenic differentiation, *n* = 3). (**D**) Cell viability detection after *PLIN5* knockdown. (**E**) Effects of *PLIN5* interference on apoptosis and cell cycle of goat adipocytes (*n* = 3). (**F**) The relative expression of proliferation-apoptosis-related genes after PLIN5 knockdown (*n* = 3). (**G**) The relative expression of Caspase-3 protein in cells after si-*PLIN5* or si-NC transfection (*n* = 3). Data are expressed as mean ± SEM. “*” *p* < 0.05, “**” *p* < 0.01.

To verify whether knockdown of *PLIN5* expression inhibits lipogenesis and promotes cell proliferation, we reduced the expression of *PLIN5* by siRNA-mediated RNA silencing (Figure 3A), which significantly reduced cellular lipid droplets by Oil red O (Figure 3B) and Bodipy stainings (Figure S3B). The suppression of *PLIN5* was significantly decreased the expression of *ACSS2*, *FATP3*, *SCD1*, *SCD5*, *ELOVL6*, *DGAT1*, *DGAT2*, *ATGL*, and *PPARγ*. There was no significant effect on the expression of *ACSL1*, *CD36*, *FASN*, *ACC*, *ELOVL3*, *HSL*, *PPARα*, *C/EBPα*, and *SREBP-1c* (Figure 3C). The knockdown of *PLIN5* significantly promoted cell proliferation at 36 h (*p* < 0.05) and 48 h (*p* < 0.05) by CCK-8 assay (Figure 3D), and reduced the apoptosis by flow cytometry experiments (Figure 3E). Cell cycle analysis showed that knockdown of *PLIN5* decreased the proportion of cells in the G0/G1 phase and increased the proportion of cells in S phase and G2/M phase (Figure 3E). Meanwhile, knockdown of *PLIN5* significantly reduced the mRNA expression levels of *CCND2*, *PCNA*, *Caspase-7*, *Caspase-3*, *Bax*, and *BcL2*, but had no significant effect on the mRNA expression levels of *CDK2* and *CDK3* (Figure 3F). Correspondingly, compared with the si-NC group, the protein expression level of Caspase-3 was significantly decreased by the silencing of *PLIN5* (Figure 3G).

### 3.4. Identification and Analysis of Differential Lipids After PLIN5 Overexpression

**Figure 4:**
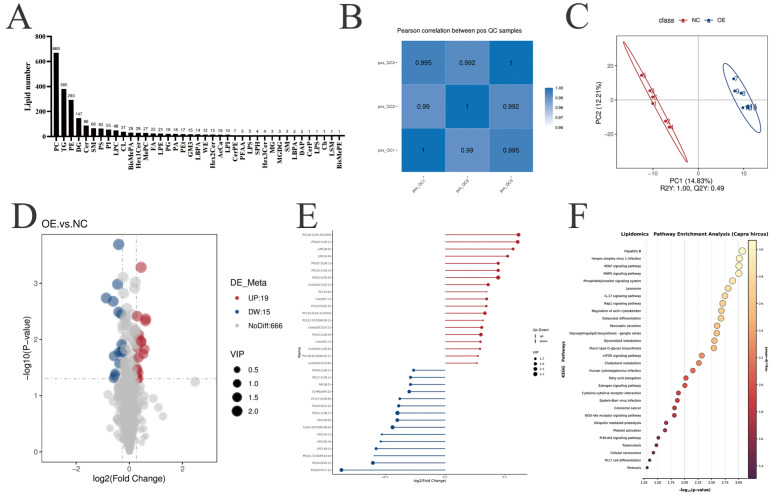
Identification and analysis of differential lipids after *PLIN5* overexpression. (**A**) Number of all lipids and lipid species identified by liquid chromatography tandem mass spectrometry lipidomics (LC-MS/MS) in positive and negative ion modes (*n* = 6). (**B**) Data Quality Control (QC) correlation type analysis. (**C**) PLS-DA partial least squares discriminant analysis (red: NC group, blue: OE group). (**D**) Differential lipid volcano plot: significantly upregulated lipid compounds are indicated by red dots, significantly downregulated lipid compounds are indicated by blue dots, and the size of the dots represents the VIP value. (**E**) Matchstick plot of lipid compounds, with the color of the dots representing up- and downregulation, blue representing downregulation, and red representing upregulation; the length of the rods representing the magnitude of log2 (fold change); and the size of the dots representing the magnitude of the VIP value. (**F**) KEGG analysis after *PLIN5* overexpression.

To fully understand the effect of *PLIN5*-mediated lipid remodeling in goat intramuscular preadipocytes, we conducted liquid chromatography tandem mass spectrometry lipidomics (LC-MS/MS) comparing *PLIN5*-overexpressing (OE) and negative control (NC) groups. A total of 2137 lipid compounds were identified in 12 samples (*n* = 6), with 1337 lipid compounds in the positive ion mode and 700 lipid compounds in the negative ion mode (Figure 4A and Supplementary Table S4A). To validate data accuracy and reliability, quality control (QC) samples were analyzed for inter-batch correlation. QC samples exhibited strong inter-sample correlations (|r| > 0.98), confirming analytical stability and high data reliability throughout the experimental workflow (Figure 4B and Supplementary Table S4B). Partial least squares discriminant analysis (PLS-DA) was performed to reveal the differences in metabolic patterns among different groups for overall lipid changes (Figure 4C and Supplementary Table S4C). Differential lipid compounds were screened according to the criteria of VIP > 1.0, FC > 1.2 or FC < 0.833, and *p*-value < 0.05. A total of 33 differentially expressed lipids were identified from 700 lipid compounds in the negative ion mode, including 19 compounds that were upregulated and 15 compounds that were downregulated (Figure 4D and Supplementary Table S4D). To visualize the most prominent lipidomic changes, fold change values of differentially abundant lipid species were log2-transformed. The 20 most significantly upregulated and downregulated lipid species were selected for visualization in a ranked-order matchstick plot. These matchstick plots show that ceramides and lysophosphatidylinositol were significantly upregulated, e.g., Cer (d35:1), Cer (d18:2/22:1), LPI (18:0), and LPI (16:0) (Figure 4E and Supplementary Table S4E). The 33 differentially expressed lipids were mainly involved in MAPK, mTOR, fatty acid elongation, and PI3K-AKT signaling pathways, as analyzed by KEGG pathway enrichment (Figure 4F and Supplementary Table S4F). In the positive ion mode, including 19 compounds that were up-regulated and 59 compounds that were down-regulated (Figure S4A and Supplementary Table S4G). The matchstick plot shows that in positive ion mode, triglycerides and diglycerides were significantly up-regulated, e.g., TG (19:0/18:1), DG (20:1/18:0), DG (20:1/16:0), DG (18:1/16:0), and TG (25:0/16:1/16:1) (Figure S4B and Supplementary Table S4H). The differentially expressed lipids were mainly involved in metabolism and organismal systems analyzed by KEGG classification enrichment (Figure S4C and Supplementary Table S4I).

### 3.5. PLIN5 Inhibits Cell Proliferation by Suppressing the PI3K-AKT Signaling Pathway

**Figure 5:**
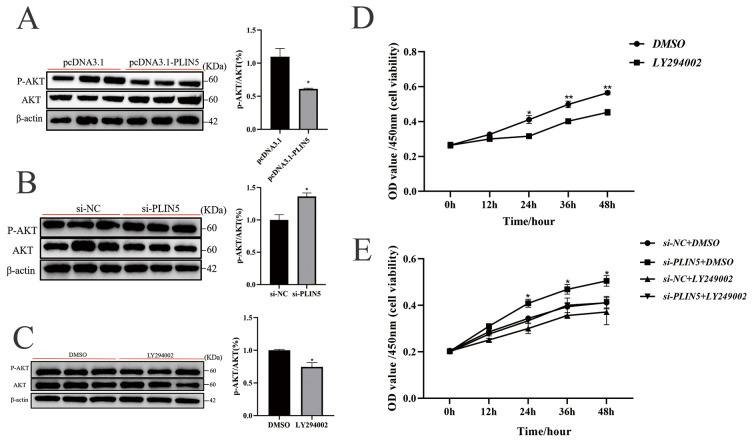
*PLIN5* inhibits cell proliferation by suppressing the PI3K-AKT signaling pathway. (**A**) Western blot was used to detect the expression of P-AKT and AKT protein and the ratio of p-AKT/AKT after *PLIN5* overexpression (*n* = 3). (**B**) Western blot was used to detect the expression of P-AKT and AKT protein, and the ratio of p-AKT/AKT after *PLIN5* knockdown (*n* = 3). (**C**) Western blot was used to detect the expression levels of P-AKT and AKT protein and the ratio of p-AKT/AKT after adding PI3K inhibitor LY294002 (Beyotime, S1737-1 mg, 5 μΜ, *n* = 3). (**D**) LY294002 inhibited goat adipocyte proliferation (*n* = 3). (**E**) The promoting effect of *PLIN5* knockdown on cell proliferation was partially offset by the PI3K inhibitor (*n* = 3). The data are expressed as mean ± SEM. “*” *p* < 0.05, “**” *p* < 0.01.

Non-targeted lipidomics analysis revealed significant enrichment of the MAPK and PI3K-AKT signaling pathways in the KEGG analysis of differentially expressed lipids. These results imply that *PLIN5*’s role in promoting the proliferation of goat preadipocytes and lipid deposition may be mediated by these pathways. To test this hypothesis, we screened the relevant pathways. The PI3K-AKT pathway was found to be significantly downregulated in cells transfected with pcDNA3.1-*PLIN5*, whereas the MAPK-p38 pathway showed no significant change (Figure 5A and Figure S3C). Accordingly, after knocking down *PLIN5*, the protein expression of p-AKT was significantly increased compared with the control group (Figure 5B). The results suggest that *PLIN5*-mediated inhibition of goat preadipocyte proliferation may be regulated through the PI3K-AKT signaling pathway. To validate this hypothesis, we used DMSO as a negative control and treated the cells with LY294002 (PI3K pathway inhibitor, 5 μΜ, Supplementary Materials Figure S5A). And then, the treatment of PI3K inhibitors (LY294002) decreased p-AKT protein expression (Figure 5C). The use of LY294002 also significantly inhibited the proliferation of goat intramuscular precursor adipocytes (Figure 5D). The combined use of si-*PLIN5* and the PI3K pathway inhibitor (LY294002) significantly inhibited cell activity compared to the si-*PLIN5* group, and the effect of knocking down *PLIN5* to promote cell proliferation was attenuated but still higher than that of the group with the addition of LY294002 inhibitor alone (Figure 5E). The result was then validated by EDU staining (Figure S5B). These results indicate that *PLIN5* inhibits cell proliferation by suppressing the PI3K-AKT signaling pathway.

### 3.6. PLIN5 Promotes Cellular Lipid Deposition by Regulating PPARγ

**Figure 6:**
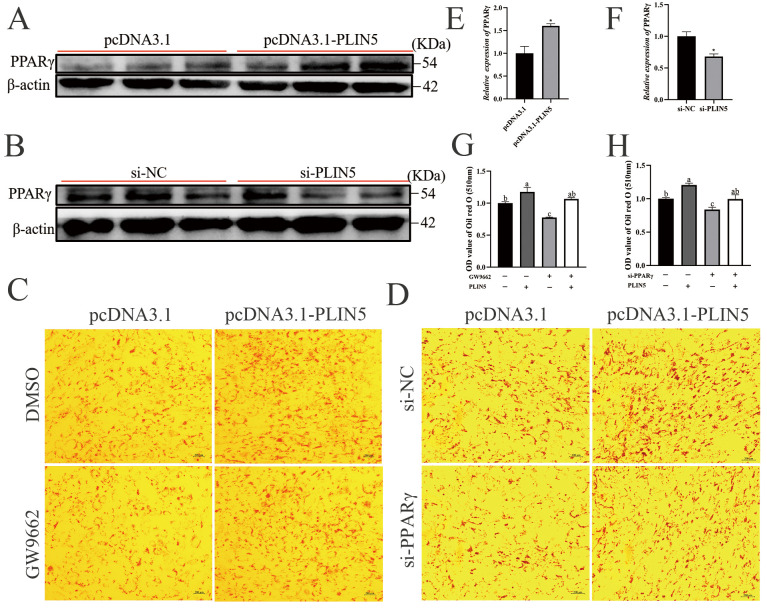
PLIN5 promotes cellular lipid deposition by regulating PPARγ. (**A**) Western blot detection of PPARγ protein expression levels after overexpression. (**B**) Western blot detection of PPARγ protein expression levels after knockdown of *PLIN5* (*n* = 3). (**C**) *PLIN5* overexpressing cells were spiked with PPARγ inhibitor (GW9662; 15 μΜ), stained with oil red O, and quantified for droplet content (day 2 after lipidogenic differentiation; 200×, *n* = 3). (**D**) pcDNA3.1-PLIN5 and si-PPARγ were co-transfected into cells, and droplet content was stained and quantified by Oil Red O staining (day 2 after lipid-forming differentiation; 200×, *n* = 3). (**E**) PPARγ protein expression levels after overexpression of *PLIN5* (*n* = 3). (**F**) PPARγ protein expression levels after knockdown of *PLIN5* (*n* = 3). (**G**) The Oil Red O levels after adding GW9662 and pcDNA3.1-*PLIN5* (*n* = 3). (**H**) The Oil Red O levels after knockdown of PPARγ and overexpression of *PLIN5* (*n* = 3). Data are expressed as mean ± SEM. “*” *p* < 0.05. Different lowercase letters indicate statistically significant differences (*p* < 0.05).

Untargeted lipidomics analysis revealed that LPI (18:0) and LPI (16:0) were significantly upregulated (Figure 4E). In visceral, LPI significantly increased PPARγ gene expression [28]. The PPAR family is a ligand-dependent transcription factor; a range of naturally occurring ligands can activate PPAR-γ, including unsaturated fatty acids, eicosanoids, and components of oxidized LDLs [23]. We hypothesized that the LPI (18:0) and LPI (16:0) significantly upregulated upon expression of *PLIN5* might lead to activation of *PPARγ*, thereby regulating lipid utilization. To verify our conjecture, we performed molecular docking prediction of the significantly upregulated lysophosphatidylinositol (LPI (16:0) and LPI (18:0)) in the OE group. The LPI (16:0) and LPI (18:0) binding pockets are structurally similar to PPARγ agonists (rosiglitazone) and may be able to bind to the ligand-binding domains of PPARγ proteins, thereby activating PPARγ (Figure S6). So, we postulated that PLIN5 may enhance lipid accumulation via LPI (16:0) and LPI (18:0), activating PPARγ pathways. For validating the hypothesis, we examined PPARγ protein expression after *PLIN5* expression. The protein expression of PPARγ was significantly increased after overexpression of *PLIN5* (Figure 6A,E) and decreased by *PLIN5* silencing (Figure 6B,F), which is the same as the mRNA level (Figure 2C and Figure 3C). Furthermore, the rescue treatment was performed by *PLIN5* overexpression after adding the si-*PPARγ* or GW9662 (PPARγ inhibitor). The results show that the effect of *PPARγ* knockdown on the reduced accumulation of lipid droplets was restored by overexpression of *PLIN5* (Figure 6C,D,G,H). These data demonstrate that *PLIN5* enhances intramuscular lipid accumulation in caprine preadipocytes via PPARγ pathways.

### 3.7. PPARγ Activation Is Required for PLIN5-Mediated Lipid Deposition and Proliferation Inhibition

**Figure 7:**
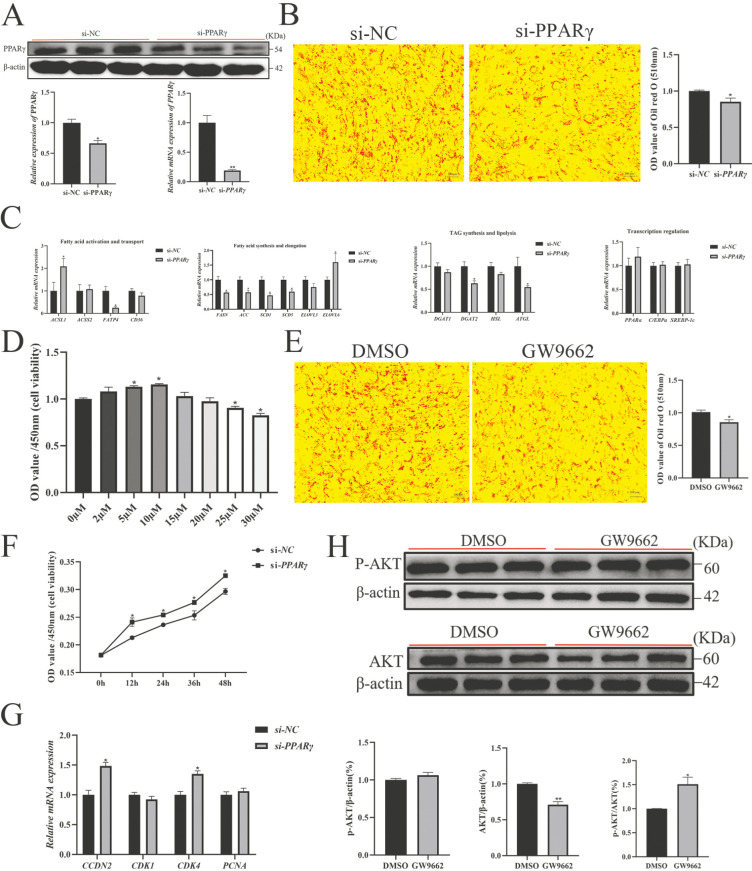
*PPARγ* activation is required for *PLIN5*-mediated lipid deposition and proliferation inhibition (**A**) si-*PPARγ* was transfected into cells, and the expression level of PPARγ was detected by RT-qPCR and Western blot (*n* = 3). (**B**) Knockdown *PPARγ*, oil red O staining, and quantitative detection of droplet content (2 days after adipogenic differentiation, 200×, *n* = 3). (**C**) The relative expression of proliferation and apoptosis-related genes after knockdown of *PPARγ* (*n* = 3). (**D**) The optimal concentration of PPARγ inhibitor GW9662 detected by CCK8 method was 15 μΜ. (**E**) PPARγ inhibitor (GW9662, 15 μΜ), oil red O staining and quantitative detection of droplet content (on the second day after adipogenic differentiation, 200×, *n* = 3). (**F**) si-*PPARγ* promoted goat adipocyte proliferation. (**G**) Detection of relative expression of proliferation-related genes after si-PPARγ transfection into cells (*n* = 3). (**H**) The protein expression of p-AKT and AKT in cells after adding GW9662 (*n* = 3). The data are expressed as mean ± SEM. “*” *p* < 0.05, “**” *p* < 0.01.

To further confirm the role of *PPARγ* in *PLIN5*-regulated lipid accumulation, we synthesized si-*PPARγ* and examined its interference efficiency using RT-qPCR and WB experiments (Figure 7A). As expected, the silencing of *PPARγ* significantly reduced cellular lipid droplets (Figure 7B) and suppressed the expression of *FATP4*, *FASN*, *ACC*, *SCD1*, *DGAT2*, and *ATGL* and increased the expression of *ACSL1* and *ELOVL3*. However, the expression of *ACSS2*, *CD36*, *ELOVL3*, *DGAT1*, *HSL*, *PPARα*, *C/EBPα*, and *SREBP-1c* expression was not significantly affected (Figure 7C). The treatment of 15 μM for the PPARγ inhibitor (GW9662) (Figure 7D) significantly reduced lipid accumulation (Figure 7E). Following which, the inhibited PPARγ expression promoted cell proliferation and increased the expression of proliferation marker genes *CCDN*2 and *CDK*3 (Figure 7F,G) and the ratio of p-AKT/AKT (Figure 7H and Figure S5C). In summary, PLIN5 promotes lipid deposition and inhibits cell proliferation by regulating *PPARγ* for lipid shunt utilization in goat intramuscular preadipocytes.

## 4. Discussion

Intramuscular fat (IMF) content is an economic characteristic of goat meat, and enhancing IMF content from a molecular point of view is an important tool for goat breeding. IMF deposition is mainly related to the proliferation and differentiation of intramuscular precursor adipocytes [2,3]. The alteration of the proliferation rate of precursor fat cells, or the augmentation of the differentiation of fat cell precursor cells to increase the number of fat cells, has become a significant method of regulating IMF deposition [29,30]. The present study was innovative in that it presented the first systematic investigation of *PLIN5*’s regulatory role in intramuscular fat deposition of goats. Through the molecular cloning of the goat *PLIN5* gene and the construction of its spatiotemporal expression profile, we found that *PLIN5* exhibits sustained high expression levels during the early stages of adipocyte differentiation. Non-targeted lipidomics sequencing and our experimental results provide compelling evidence that *PLIN5* modulates the PPARγ and PI3K-AKT signaling pathways to alter precursor adipocyte proliferation. Higher expression of LPI (16:0) or LPI (18:0) may regulate lipid droplet accumulation by activating PPARγ. These findings establish *PLIN5* as a pivotal mediator directing fatty acid flux towards either lipid synthesis or cellular proliferation via the control of *PPARγ* which not only addresses a critical knowledge gap regarding *PLIN5*’s role in the regulation of intramuscular fat deposition in goat muscle but also establishes a substantial theoretical foundation for molecular-level optimization of goat meat quality.

PLIN5 (Perilipin 5), a key lipid droplet-associated protein regulating lipid metabolism, has remained a focal point in metabolic research since its concurrent identification by three independent groups (2006–2007) [8,9,10], and is specifically overexpressed in tissues with high levels of fatty acid oxidation and functions to promote lipid formation [10]. Moreover, *PLIN5* is also involved in mediating the contact site between lipid droplets and mitochondrial membranes to regulate lipid metabolism and energy metabolism, and is essential for cell proliferation, differentiation, and apoptosis [12,13]. In addition, *PLIN5* has been extensively studied in the field of disease research [31,32], especially in hepatocellular carcinoma [33]. In domestic animals, the research of *PLIN5* mainly focused on economic animals such as pigs [34,35] and cattle [16,36], but research involving goats remains largely unexplored. In the present study, the expression of *PLIN5* increased with the muscular development in goats during the first 9 months, and then turned down in 23 months (Figure 1A). This observation was then supported by the study during preadipocyte differentiation, which peaked at 2 days and reached the lowest point at 8 days (Figure 1B). This expression pattern contrasts with that observed in pigs, where *PLIN5* expression showed a decreasing and then increasing trend [35]. It is particularly noteworthy that, using cardiac muscle tissue as a control, we found that the expression level of *PLIN5* was particularly prominent in goat liver and the longest dorsal muscle (Figure S1C). Consistent with this data, *PLIN5* mRNA and protein are highly expressed in mouse tissues that have large capacities for fatty acid oxidation [9]. Collectively, these findings provide important clues for studying the role of PLIN5 in lipogenesis in goat intramuscular preadipocytes; it is reasonable to assume that *PLIN5* is a key gene in the regulation of IMF in goats.

Our findings indicate that *PLIN5* overexpression significantly increased triglyceride content and lipid accumulation in goat adipocytes, which is consistent with the general consensus that *PLIN5* plays an important role in lipid droplet accumulation. In detail, radioactive pulse tracing in C2C12 myoblasts showed that TAG content increased significantly after PLIN5 expression [12]. Similarly, the expression of PLIN5 promotes lipid droplet storage in the heart of mice [37], and mild overexpression of *PLIN5* via DNA electrotransfer into the glycolytic muscle of rats increased triacylglycerol accumulation [14]. Under the positive ion mode, our sequencing results showed that a large number of TGs and DGs were significantly upregulated, further supporting this finding (Figure S4B). It is well known that triglyceride production and fatty acid uptake are key to lipid deposition. SCD1 [38] and SCD5 [39] can catalyze the formation of monounsaturated fats, fatty acyl-CoA (18:1) and palmitoyl-CoA (16:1), which are the main substrates for TAG synthesis. SCD1 overexpression in dairy goats increases intracellular monounsaturated fatty acid (MUFA) content and lipid accumulation [40]. Glycerol diacylglycerol transferase (DGAT) 1 and 2 are two rate-limiting enzymes that catalyze the final step in the synthesis of triacylglycerol (TG) by condensation of diacylglycerol and fatty acyl coenzyme A to form TG [41,42]. Mice lacking DGAT1 have reduced TG stores, whereas DGAT2 knockout mice die shortly after birth with >90% reduction in TG [43]. Our study showed that PLIN5 significantly promoted the expression of SCD1, SCD5, DGAT1, and DGAT2 in intramuscular adipocytes (Figure 2C), which was likely to accelerate the de novo synthesis rate of triglycerides, resulting in an increase in TG content in goat preadipocytes.

On the other hand, we observed upregulation of PLIN5, which increased PPARγ expression at both the mRNA level and the protein level (Figure 2C, Figure 3C and Figure 6B). PPARγ serves as a master transcriptional regulator of adipogenesis, lipid homeostasis, and lipogenic pathways. Functioning as a ligand-activated nuclear receptor, PPARγ binds to both of its ligands and triggers changes in gene expression and the rate of adipogenesis; e.g., activation of PPARγ by thiazolidinediones leads to a decrease in free fatty acid (FFA) levels and an increase in lipid stores in adipose tissue, resulting in reduced muscle and liver lipotoxicity [44]. Non-targeted lipidomics sequencing identified 19 upregulated differential lipids (Figure 4D,E, mostly LPIs and Cers) in negative ion mode, which may have acted as PPARγ ligands, increasing PPARγ activity and leading to lipid deposition. Computer analysis revealed that LPI (16:0) and LPI (18:0) could activate PPARγ (Figure 6A). This is consistent with the finding of José María Moreno-Navarrete et al. that in human visceral and subcutaneous adipose tissue (VAT and SAT, respectively), LPI significantly increased peroxisome proliferator-activated receptor gamma (PPARγ) gene expression [28]. In human pulmonary arteries (hPAs), LPI activates PPARγ to regulate vasodilatory effects [45]. It has also been observed that LPI induced and promoted adipocyte differentiation by upregulation of PPARγ [46]. Similarly, in bovine mammary epithelial cells, palmitate (16:0) and stearate (18:0) are PPARγ ligands that alter lipogenic gene networks [24]. Furthermore, the LPI biological activities can be divided into non-receptor and receptor-mediated [47], the latter of which in mice activates G protein-coupled receptor 55 (GPR55) to induce the expression of fat deposition-related genes via the PI3K/Akt/SREBP-1c signaling pathway [48]. Under PLIN5 overexpression, LPI (16:0) and LPI (18:0) may be candidate activating ligands for PPARγ that can alter the adipogenic gene network in goat precursor adipocytes. Crucially, the effect of PPARγ knockdown on the reduced accumulation of lipid droplets was restored by overexpression of PLIN5 (Figure 6D), and the same result was obtained with the addition of GW9662, an inhibitor of PPARγ (Figure 6C), confirming PLIN5-PPARγ interplay functional in lipid. In addition, PPARγ significantly promoted lipid storage in goat mammary epithelial cells (GMEC) [49]. Taken together, PLIN5 can regulate lipid metabolism by accelerating TAG de novo synthesis and activating PPARγ, leading to lipid deposition. In addition, after interfering with PLIN5, the downregulation of *SCD1*, *SCD5*, *DGAT1*, *DGAT2* and *PPARγ* reduced lipid deposition, which also proved the reliability of the results.

IMF content is also related to the number of adipocytes, which is regulated by proliferation and apoptosis of precursor adipocytes [50]. In this study, PLIN5 regulates PPARγ to promote lipid deposition while also inhibiting cell proliferation and promoting apoptosis in precursor adipocytes. This is consistent with the function played by PLIN5 in non-alcoholic fatty liver disease (NAFLD) liver disease [51], specifically overexpression of PLIN5 in activated HSCs inhibition of cell proliferation, and a significant increase in cell apoptosis. Conversely, silencing PLIN5 in goat precursor adipocytes promoted cell proliferation, aligning with the findings of Xueqing Gan et al. in vascular smooth muscle cell (VSMC) [52]. In our study, untargeted lipidomics analysis revealed significant enrichment of the MAPK, mTOR, fatty acid elongation, and PI3K-AKT signaling pathways in the KEGG analysis of differentially expressed lipids. Among these signaling cascades, the PI3K-AKT and MAPK pathways emerge as pivotal intracellular signaling axes [53]. Screening of these two pathways revealed that PLIN5 is associated with the PI3K-AKT pathway (Figure 5A). Inhibition of PLIN5 increased the p-AKT/AKT ratio (Figure 5B) and promoted cell proliferation (Figure 3D). The PI3K-AKT pathway is closely related to cell proliferation and can control cell growth and survival [54,55]. We speculate that *PLIN5* may inhibit cell proliferation via the PI3K-AKT pathway. For this purpose, we used LY294002, which is a specific PI3K inhibitor that inhibits PI3K phosphorylation, which in turn affects the activation of the downstream AKT signaling pathway [56,57]. We inhibited the PI3K-AKT signaling pathway by adding a PI3K inhibitor (LY294002) to goat intramuscular preadipocytes and found that the cell proliferation capacity brought about by *PLIN5* deficiency was attenuated, suggesting that *PLIN5* regulates cell proliferation through inhibition of AKT signaling (Figure 5E). Apoptosis is another important factor affecting cell number, was consistent with the inhibition of intramuscular preadipocyte proliferation by PLIN5, and the cell cycle was blocked in S and G2/M phases (Figure 2E). Compared with the control group, overexpression of *PLIN5* resulted in an elevated apoptosis rate and a significant increase in the expression of the apoptosis marker gene *Caspase-3* (Figure 2G). Interestingly, silencing *PLIN5* promoted cell proliferation by activation of the PI3K-AKT pathway and significantly inhibited apoptosis. But the mRNA expression levels of *CCND2* and *PCNA* were downregulated. (Figure 3F). Reduced *CCND2* expression generally leads to decreased *Cyclin D-CDK4/6* complex activity, resulting in impaired cell cycle progression and suppressed proliferation [58], while expression of *CDK2* and *CDK4* remained unchanged in this research. Reduced *PCNA* levels impair DNA synthesis, resulting in compromised cell cycle progression [59]. However, silencing *PLIN5* cell cycle analysis revealed an increased S-phase population accompanied by a decreased G0/G1 fraction. These findings suggest that the PI3K-AKT pathway may be strongly activated, bypassing *CCND2*, potentially upregulating Cyclin D1 [60], it also can upregulate replication initiation factors such as *MCM* [61], partially compensating for the absence of *PCNA*, thereby triggering a compensatory response that enhances cellular proliferation. Therefore, the mechanism by which silencing *PLIN5* affects cell proliferation deserves further investigation.

In oncology research, *PTEN* is a well-known repressor gene involved in the regulation of the PI3K/AKT pathway that dephosphorylates PIP3 to PIP2 and inhibits the downstream activation of AKT and mTOR signaling [62]. Meanwhile, PTEN is an effector downstream of *PPARγ* [63], and the formation of a heterodimer by *PPARγ* and retinoid X receptor (RXR) directly activates PTEN, thereby inhibiting AKT [64]. In the PPARγ/PTEN/AKT signaling pathway, upregulation of PPARγ and PTEN proteins inhibits the AKT-activated cascade response, as demonstrated in both pancreatic cancer [65] and bladder cancer [66]. We hypothesized that *PPARγ* could maintain the dynamic balance between lipid deposition and cell proliferation in goat intramuscular precursor adipocytes by regulating lipid utilization. Our mechanistic investigations revealed that *PPARγ* knockdown suppressed intramuscular lipid accumulation and significantly altered the expression of lipid metabolism signature genes (Figure 7B,C). Notably, *PPARγ* silencing enhanced preadipocyte proliferation (Figure 7F) and elevated the activated P-AKT/AKT ratio (Figure S5C), establishing a functional linkage between PPARγ activity and PI3K/AKT pathway modulation in goat intramuscular adipogenesis. This hypothesis provides a potential molecular framework for understanding the role of *PLIN5* in lipid metabolism regulation.

Current evidence indicates that precursor adipocytes must differentiate into adipocytes to activate lipid synthesis [67]. Thus, precursor adipocytes must exit the cell cycle at some point during lipogenesis and initiate a differentiation program (such as PPARγ activation) to differentiate into mature adipocytes [2], thereby efficiently synthesizing and storing lipids. This process involves the synergistic interaction between cell cycle regulation and the differentiation transcription network [68]. *PLIN5* may play a role in this transition process. Our experimental data demonstrate that, in the absence of oleic acid, *PLIN5* overexpression reduces the proportion of cells in S phase and G2/M phase, increases the proportion in G0/G1 phase, significantly inhibits cell proliferation, accelerates apoptosis in preadipocytes. This most likely advances the timing of preadipocyte exit from the cell cycle. Then, oleic acid addition induced cellular differentiation, and overexpression of *PLIN5* leads to a significantly increased expression of *PPARγ* compared to controls. The expression of *PPARγ* is not only crucial for adipogenesis but also essential for maintaining a differentiated state, and is to some extent regarded as a genetic marker for adipogenic cells [69]. In line with our experimental observations, Yin, X. et al. demonstrated that overexpressing *PLIN5* in primary rat hepatic stellate cells (rHSC-primary) leads to the upregulation of *Bax*, as well as increased *PARP*, *caspase-3*, and *caspase-9* [51]. Based on these findings, we propose that PLIN5 may accelerate the maturation process of preadipocytes into adipocytes, thereby inducing apoptosis, inhibiting proliferation, and increasing lipid deposition in preadipocytes of goats.

While our findings provide novel insights into *PLIN5*’s role in goat IMF deposition, we acknowledge that the sample size (*n* = 3) presents certain limitations. Expanding future studies to include larger animal cohorts, incorporating diverse breeds and genders, will be crucial for validating these mechanisms and enhancing their generalizability within livestock IMF research.

## 5. Conclusions

**Figure 8:**
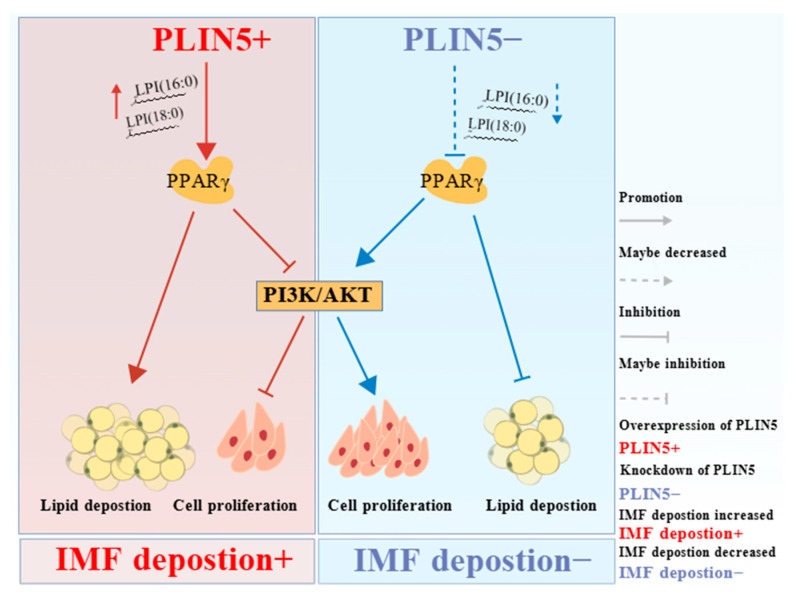
Schematic representation of PLIN5 regulation of IMF deposition. Increased LPI (16:0) and LPI (18:0) in cells were found to potentially activate PPARγ by PLIN5 overexpression non-targeted lipidomics sequencing, and KEGG pathway enrichment analysis revealed a significant enrichment of the PI3K-AKT signaling pathway. PLIN5 overexpression upregulated PPARγ expression to promote lipid deposition but inhibited the PI3K-AKT signaling pathway to suppress cell proliferation, leading to increased IMF deposition. PLIN5 knockdown activated the PI3K-AKT signaling pathway through downregulation of PPARγ, which altered intracellular lipid distribution, resulting in increased cell proliferation and decreased lipid droplets, ultimately leading to decreased IMF deposition. Figure 8 created with BioGDP.e.com.

In summary, *PLIN5* regulates intramuscular lipid deposition in goats by modulating *PPARγ* and regulates intramuscular cell proliferation by PI3K-AKT signaling pathways. LPI (18:0) and LPI (16:0) may be involved in the control of the activation of *PPARγ* by *PLIN5* expression variation. These findings greatly contribute to our understanding of the functional role of *PLIN5* in the regulation of lipid metabolism and provide mechanistic insights into its potential regulatory network in goat intramuscular lipid formation (Figure 8).

## Data Availability

The datasets generated for this study can be found in the NCBI Bio Project database. (https://www.ncbi.nlm.nih.gov/nuccore/PP823817.1, GenBank: PP823817.1, 29 May 2023). The raw files of the mass spectrometry assays are deposited in the CNCB database (https://ngdc.cncb.ac.cn/omix/select-edit/OMIX012544, BioProject: PRJCA049258, 1 November 2025).

## Supplementary Materials

The following supporting information can be downloaded at: https://www.mdpi.com/article/10.3390/biology14111547/s1, Figure S1: Cloned a full-length cDNA for goat *PLIN5*; Figure S2: Characterization of a full-length cDNA for goat *PLIN5*; Figure S3: Bodipy staining; Figure S4 Identification and analysis of differential lipids after *PLIN5* overexpression; Figure S5: *PLIN5* inhibits cell proliferation by suppressing the PI3K-AKT signaling pathway; Figure S6: Molecular docking analysis; Figure S7: Western Blot Original Images of Figure 1C, Figure 2A,G, Figure 3A, Figure 5A,B, Figure 6A,B and Figure 7A,H; Figure S8: Uncropped, full-length Western blot Images of Figure 1C, Figure 2A,G, Figure 3A, Figure 5A,B, Figure 6A,B and Figure 7A,H; Table S1: Primers for *PLIN5* cloning and subcloning; Table S2: Sequence analysis tools and corresponding analysis content; Table S3: Summary of genes, primers for RT-qPCR analysis; Table S4A–I: The lipidomic sequencing data of intramuscular preadipocytes transfected with a pcDNA3.1 and with a pcDNA3.1-*PLIN5* were analyzed.
