# Supplementary material for: PLIN5 Promotes Lipid Reconstitution in Goat Intramuscular Fat via the PPARγ Signaling Pathway
Source: Biology (Basel). 2025 Nov 4;14(11):1547. doi: 10.3390/biology14111547 (PMC12649955; doi:10.3390/biology14111547)
Supplement: Supplementary file 1 [file biology-14-01547-s001.zip › Supplementary figure S7.pdf]

Supplementary materials

Original Western Blot and Electrophoretic Images

The original, uncropped and unadjusted western blot images of each protein in our manuscript are displayed as follows. Owing to the multiple proteins needed to be detected in the experiments and saving antibody, we did not incubate the whole membranes.

Figure 1C- the expression of *PLIN5* in goat intramuscular preadipocytes during differentiation was detected

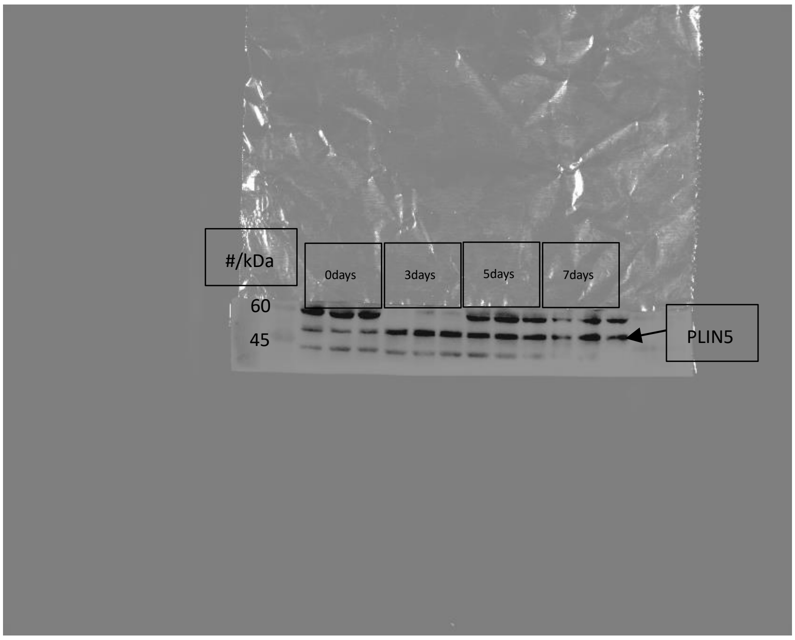

Figure 1C- Western blot membrane of PLIN5 (~55 kDa) protein detected with anti-PLIN5 (A25598; 1:1000; ABclonal, Wuhan, China) antibody. Gel-separated proteins were transferred to PVDF membranes (0.45 µm pore size; Millipore, Billerice, USA) by semidry electroblotting (1.3 A ,25V, 20 min). Membranes, incubated with a horseradish peroxidase-conjugated secondary antibody (BA1054; 1:5000–1:10000; Boster), were developed with Oriscience Supersensitive Kit (Oriscience Biotechnology). #Weight marker (molecular weight in kDa): Blue Plus IV Protein marker, 10 to 180 kDa; catalogue number: R10519. Blot images, prior to the densitometry readings, were converted to grayscale with ImageJ (ImageJ, National Institutes of Health, Maryland, USA) as follows: Image -> Type –> 8 bit, next: Image -> Adjust–> Brightness/Contrast –>Auto.(0days-PLIN5/β-actin):(3days-PLIN5/β-actin):(5days-PLIN5/β-actin):(7days-PLIN5/β-actin) = 1:2.75:2.31:1.42.

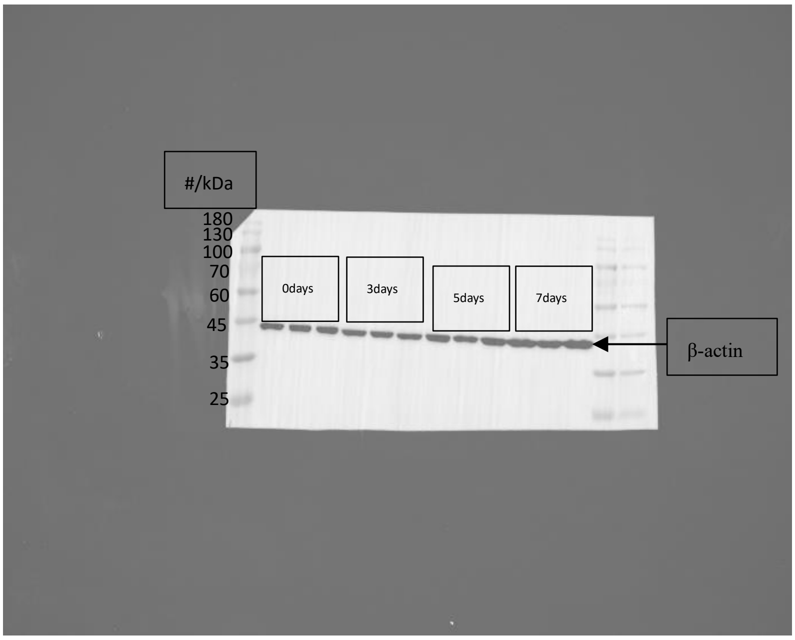

Figure 1C- Western blot membrane of β-actin (~42 kDa) protein detected with anti-β-actin (BM0627; 1:8000; Boster, Wuhan, China) antibody. Gel-separated proteins were transferred to PVDF membranes (0.45 μm pore size; Millipore, Billerice, USA) by semidry electroblotting (1.3 A ,25V, 20 min). Membranes, incubated with a horseradish peroxidase-conjugated secondary antibody (BA1050; 1:5000–1:10000; Boster), were developed with Oriscience Supersensitive Kit (Oriscience Biotechnology). #Weight marker (molecular weight in kDa): Blue Plus IV Protein marker, 10 to 180 kDa; catalogue number: R10519. Blot images, prior to the densitometry readings, were converted to grayscale with ImageJ (ImageJ, National Institutes of Health, Maryland, USA) as follows: Image -> Type –> 8 bit, next: Image -> Adjust–> Brightness/Contrast –> Auto.(0days-PLIN5/β-actin):(3days-PLIN5/β-actin):(5days-PLIN5/β-actin):(7days-PLIN5/β-actin) = 1:2.75:2.31:1.42.

Figure 2A- the expression level after overexpression of *PLIN5*

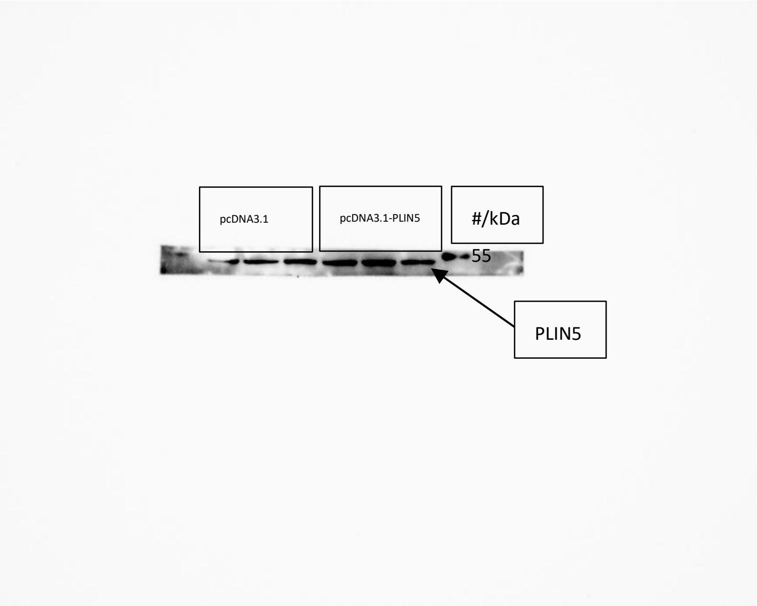

Figure 2A- Western blot membrane of PLIN5 (~55 kDa) protein detected with anti-PLIN5

(A25598; 1:1000; ABclonal, Wuhan, China) antibody. Gel-separated proteins were transferred to PVDF membranes (0.45 μm pore size; Millipore, Billerice, USA) by semidry electroblotting (1.3 A,25V 2, 15 min). Membranes, incubated with a horseradish peroxidase-conjugated secondary antibody (BA1054; 1:5000–1:10000; Boater), were developed with Oriscience Supersensitive Kit (Oriscience Biotechnology). #Weight marker (molecular weight in kDa): Blue Plus IV Protein marker, 10 to 180 kDa; catalogue number: R21223-V2. Blot images, prior to the densitometry readings, were converted to grayscale with ImageJ (ImageJ, National Institutes of Health, Maryland, USA) as follows: Image -> Type –> 8 bit, next: Image -> Adjust–> Brightness/Contrast –> Auto. (pcDNA3.1/β-actin): (pcDNA3.1-*PLIN5*/β-actin) = 1:1.58.

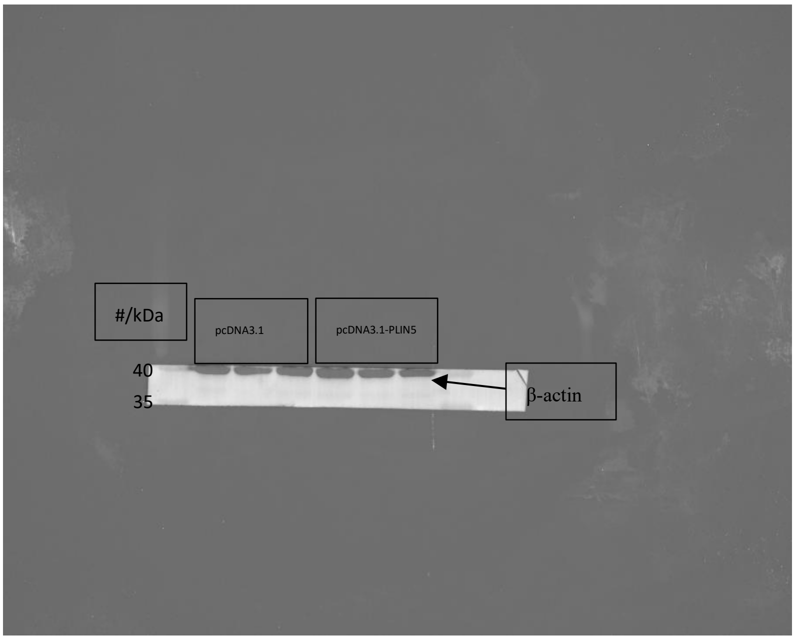

Figure 2A- Western blot membrane of β-actin (~42 kDa) protein detected with anti-β-actin (BM0627; 1:8000; Boster, Wuhan, China) antibody. Gel-separated proteins were transferred to PVDF membranes (0.45 μm pore size; Millipore, Billerice, USA) by semidry electroblotting (1.3 A,25V, 20 min). Membranes, incubated with a horseradish peroxidase-conjugated secondary antibody (BA1050; 1:5000–1:10000; Boster), were developed with Oriscience Supersensitive Kit (Oriscience Biotechnology). #Weight marker (molecular weight in kDa): Blue Plus IV Protein marker, 10 to 180 kDa; catalogue number: R21223-V2. Blot images, prior to the densitometry readings, were converted to grayscale with ImageJ (ImageJ , National Institutes of Health, Maryland, USA) as follows: Image -> Type –> 8 bit, next: Image -> Adjust–> Brightness/Contrast –> Auto. (pcDNA3.1/β-actin): (pcDNA3.1-*PLIN5*/β-actin) = 1:1.58.

Figure 2G -Caspase3 expression level after overexpression of *PLIN5*

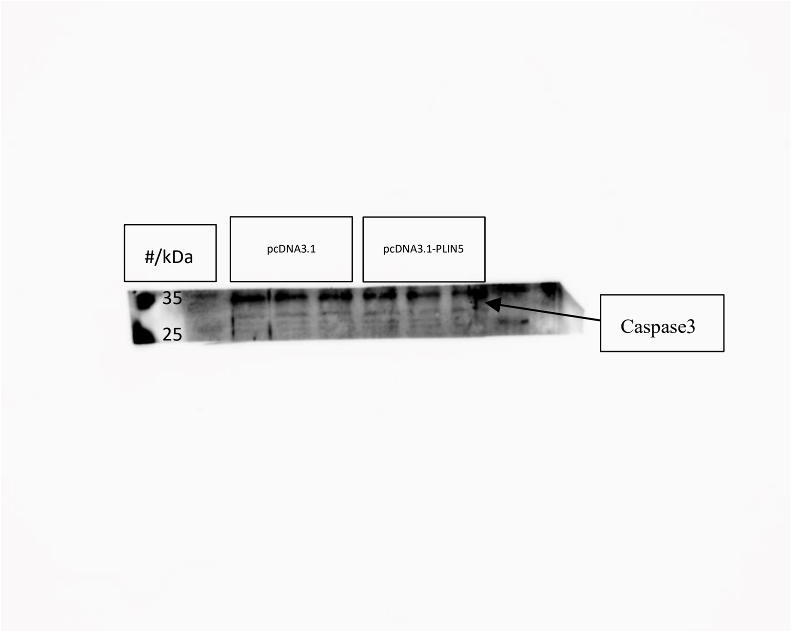

Figure 2G- Western blot membrane of Casepase3 (~32 kDa) protein detected with anti-Caspase3 (WL02117; 1:500; Wanleibio, Shenyang, China) antibody. Gel-separated proteins were transferred to PVDF membranes (0.45 µm pore size; Millipore, Billerice, USA) by semidry electroblotting (1.3 A, 25V , 10 min). Membranes, incubated with a horseradish peroxidase-conjugated secondary antibody (BA1054; 1:5000–1:10000; Boster), were developed with Oriscience Supersensitive Kit (Oriscience Biotechnology). #Weight marker (molecular weight in kDa): Blue Plus IV Protein marker, 10 to 180 kDa; catalogue number: R21223-V2. Blot images, prior to the densitometry readings, were converted to grayscale with ImageJ (ImageJ , National Institutes of Health, Maryland, USA) as follows: Image -> Type –> 8 bit, next: Image -> Adjust–> Brightness/Contrast –> Auto. pcDNA3.1(Casepase3/β-actin): pcDNA3.1-*PLIN5*(Casepase3/β-actin) = 1:1.60.

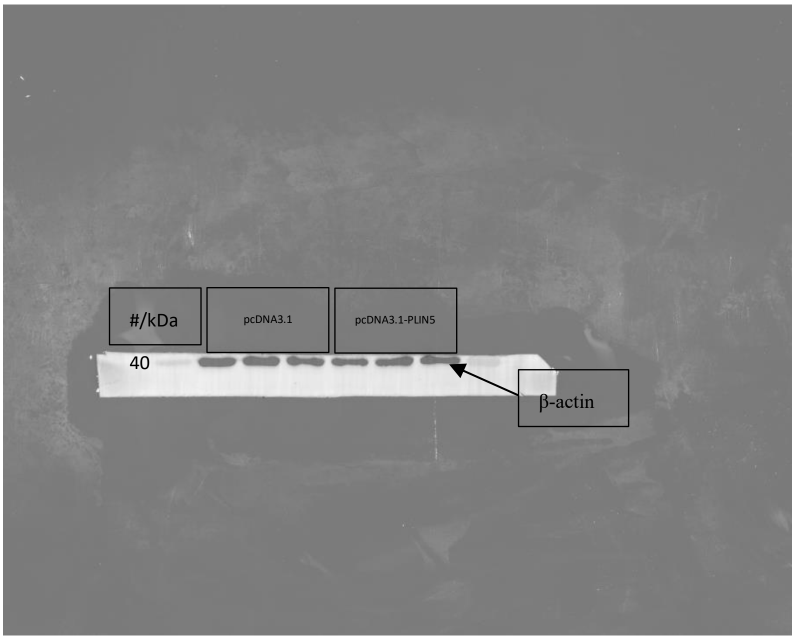

Figure 2G- Western blot membrane of β-actin (~42 kDa) protein detected with anti-β-actin (BM0627; 1:8000; Boster, Wuhan, China) antibody. Gel-separated proteins were transferred to PVDF membranes (0.45 μm pore size; Millipore, Billerice, USA) by semidry electroblotting (1.3 A ,25V, 20 min). Membranes, incubated with a horseradish peroxidase-conjugated secondary antibody (BA1050; 1:5000–1:10000; Boster), were developed with Oriscience Supersensitive Kit (Oriscience Biotechnology). #Weight marker (molecular weight in kDa): Blue Plus IV Protein marker, 10 to 180 kDa; catalogue number: R21223-V2. Blot images, prior to the densitometry readings, were converted to grayscale with ImageJ (ImageJ , National Institutes of Health, Maryland, USA) as follows: Image -> Type –> 8 bit, next: Image -> Adjust–> Brightness/Contrast –> Auto. pcDNA3.1(Casepase3/β-actin): pcDNA3.1-*PLIN5*(Casepase3/β-actin) = 1:1.60.

Figure 3A- the expression level after knockdown of *PLIN5*

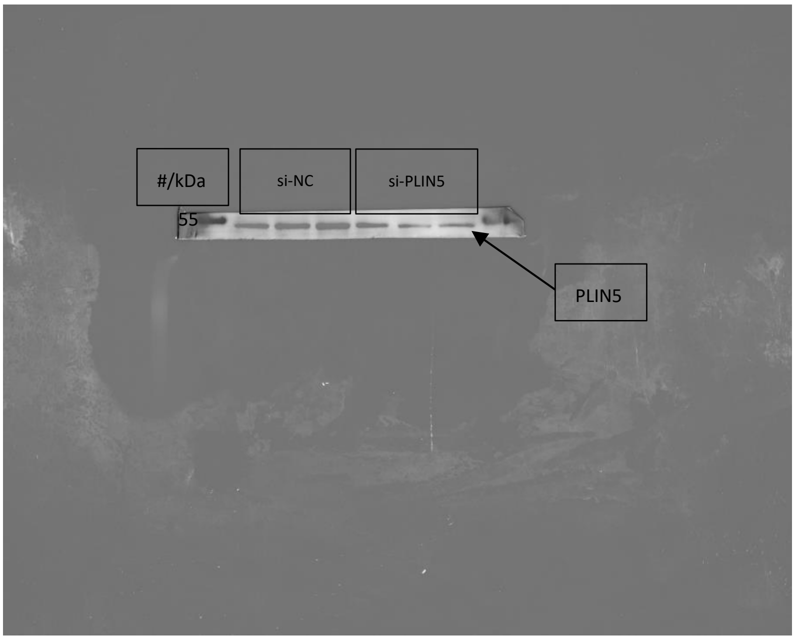

Figure 3A- Western blot membrane of PLIN5 (~55 kDa) protein detected with anti-PLIN5 (A25598; 1:1000; ABclonal, Wuhan, China) antibody. Gel-separated proteins were transferred to PVDF membranes (0.45 μm pore size; Millipore, Billerice, USA) by semidry electroblotting (1.3 A,25V 2, 15 min). Membranes, incubated with a horseradish peroxidase-conjugated secondary antibody (BA1054; 1:5000–1:10000; Boater), were developed with Oriscience Supersensitive Kit (Oriscience Biotechnology). #Weight marker (molecular weight in kDa): Blue Plus IV Protein marker, 10 to 180 kDa; catalogue number: R21223-V2. Blot images, prior to the densitometry readings, were converted to grayscale with ImageJ (ImageJ, National Institutes of Health, Maryland, USA) as follows: Image -> Type –> 8 bit, next: Image -> Adjust–> Brightness/Contrast –> Auto. (Si-*NC*/β-actin): (si-*PLIN5*/β-actin) = 1:0.72.

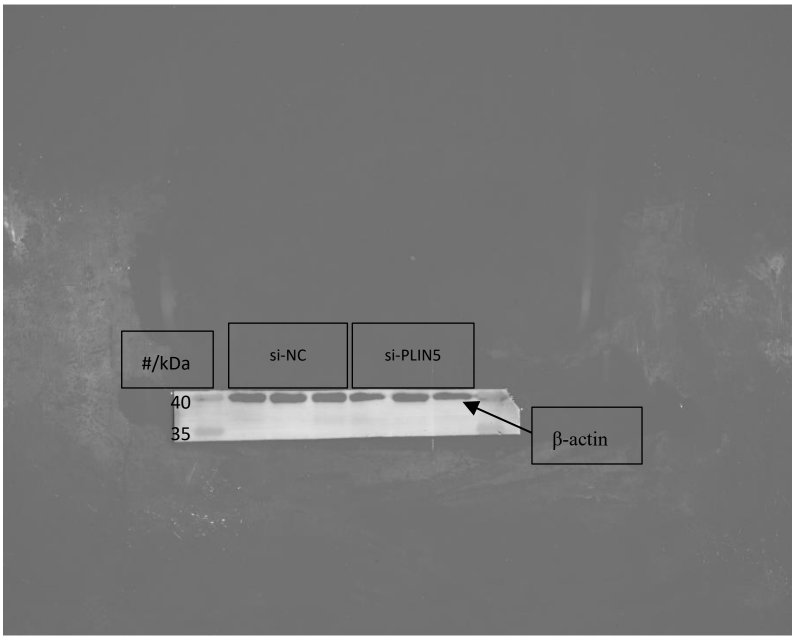

Figure 3A - Western blot membrane of β-actin (~42 kDa) protein detected with anti-β-actin (BM0627; 1:8000; Boster, Wuhan, China) antibody. Gel-separated proteins were transferred to PVDF membranes (0.45 μm pore size; Millipore, Billerice, USA) by semidry electroblotting (1.3 A ,25V, 20 min). Membranes, incubated with a horseradish peroxidase-conjugated secondary antibody (BA1050; 1:5000–1:10000; Boster), were developed with Oriscience Supersensitive Kit (Oriscience Biotechnology). #Weight marker (molecular weight in kDa): Blue Plus IV Protein marker, 10 to 180 kDa; catalogue number: R21223-V2. Blot images, prior to the densitometry readings, were converted to grayscale with ImageJ (ImageJ, National Institutes of Health, Maryland, USA) as follows: Image -> Type –> 8 bit, next: Image -> Adjust–> Brightness/Contrast –> Auto. (Si-*NC*/β-actin): (si-*PLIN5*/β-actin) = 1:0.72.

igure 3G- Caspase3 expression level after knockdown of *PLIN5*

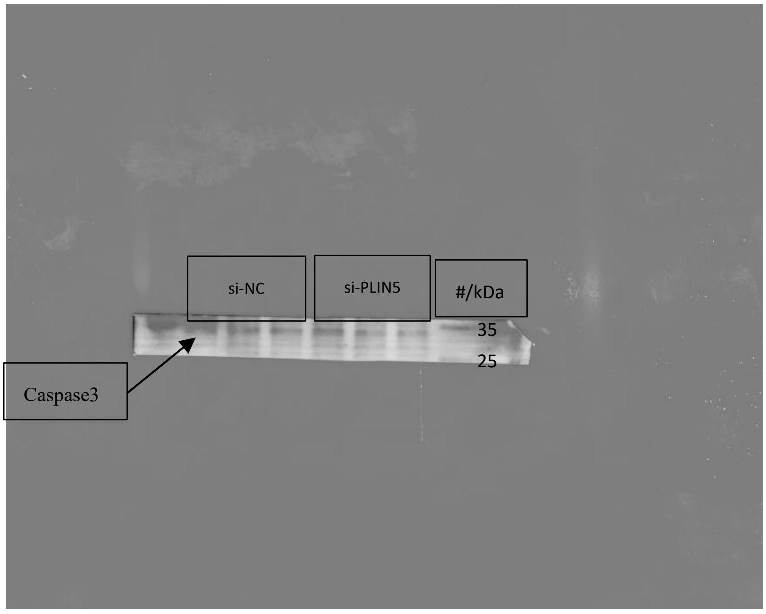

Figure 3G- Western blot membrane of Casepase3 (~32 kDa) protein detected with anti-Casepase3 (WL02117; 1:500; Wanleibio, Shenyang, China) antibody. Gel-separated proteins

were transferred to PVDF membranes (0.45 μm pore size; Millipore, Billerice, USA) by semidry electroblotting (1.3 A,25V 2, 10 min). Membranes, incubated with a horseradish peroxidase-conjugated secondary antibody (BA1054; 1:5000–1:10000; Boster), were developed with Oriscience Supersensitive Kit (Oriscience Biotechnology). #Weight marker (molecular weight in kDa): Blue Plus IV Protein marker, 10 to 180 kDa; catalogue number: R21223-V2. Blot images, prior to the densitometry readings, were converted to grayscale with ImageJ (ImageJ, National Institutes of Health, Maryland, USA) as follows: Image -> Type –> 8 bit, next: Image -> Adjust–> Brightness/Contrast –> Auto. Si-*NC*(Casepase3/β-actin): Si-*PLIN5*(Casepase3/β-actin) = 1:0.60.

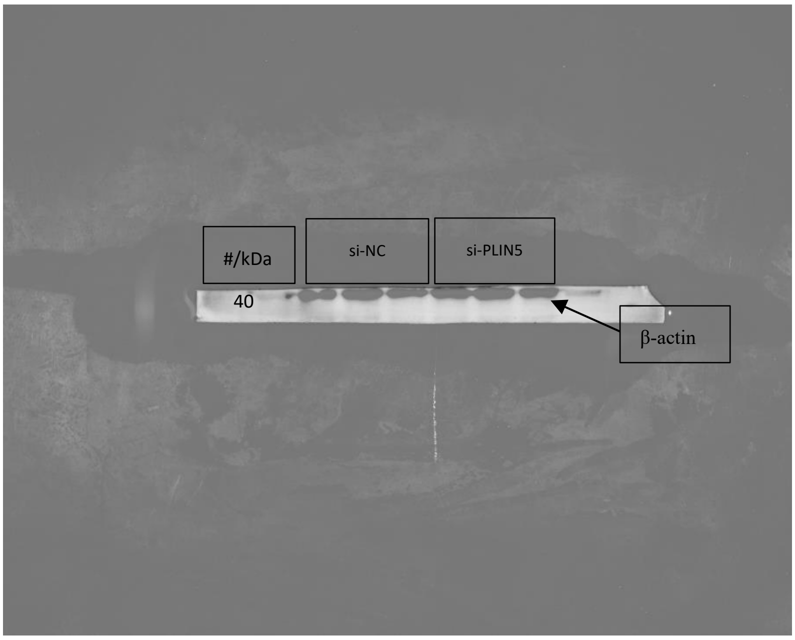

Figure 3G - Western blot membrane of β-actin (~42 kDa) protein detected with anti-β-actin (BM0627; 1:8000; Boster, Wuhan, China) antibody. Gel-separated proteins were transferred to PVDF membranes (0.45 μm pore size; Millipore, Billerice, USA) by semidry electroblotting (1.3 A ,25V, 20 min). Membranes, incubated with a horseradish peroxidase-conjugated secondary antibody (BA1050; 1:5000–1:10000; Boster), were developed with Oriscience Supersensitive Kit (Oriscience Biotechnology). #Weight marker (molecular weight in kDa): Blue Plus IV Protein marker, 10 to 180 kDa; catalogue number: R21223-V2. Blot images, prior to the densitometry readings, were converted to grayscale with ImageJ (ImageJ, National Institutes of Health, Maryland, USA) as follows: Image -> Type –> 8 bit, next: Image -> Adjust–> Brightness/Contrast –> Auto. Si-*NC*(Casepase3/β-actin): Si-*PLIN5*(Casepase3/β-actin) = 1:0.60.

Figure 5A- p-AKT/AKT expression level after overexpression of *PLIN5*

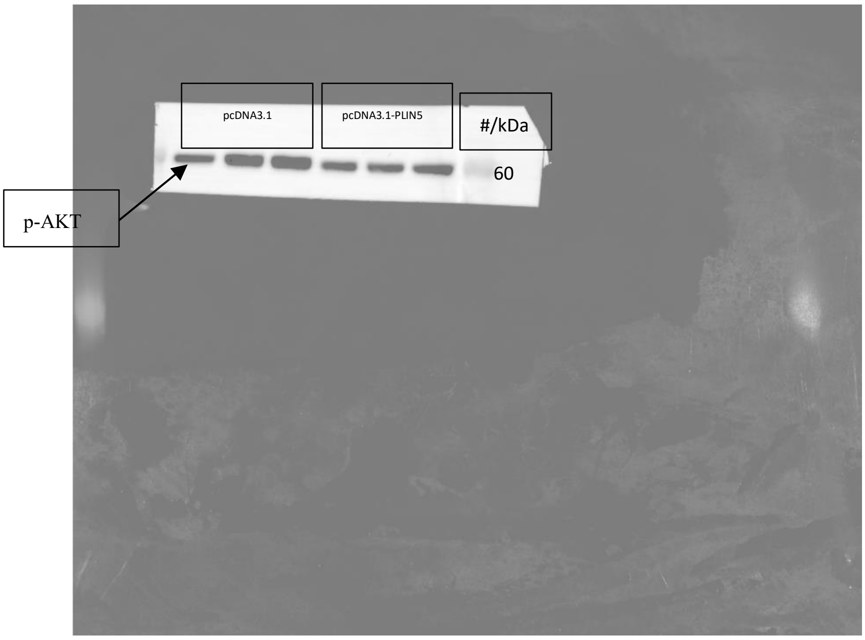

Figure 5A- Western blot membrane of p-AKT (~60 kDa) protein detected with anti-p-AKT1-Ser473 (4060; 1:2000; Cell Signaling Technology, Massachusetts, USA) antibody. Gel-separated proteins were transferred to PVDF membranes (0.45 µm pore size; Millipore, Billerice, USA) by semidry electroblotting (1.3 A,25V, 15 min). Membranes, incubated with a horseradish peroxidase-conjugated secondary antibody (BA1054; 1:5000–1:10000; Boster), were developed with Oriscience Supersensitive Kit (Oriscience Biotechnology). #Weight marker (molecular weight in kDa): Blue Plus IV Protein marker, 10 to 180 kDa; catalogue number: R10519. Blot images, prior to the densitometry readings, were converted to grayscale with ImageJ (ImageJ , National Institutes of Health, Maryland, USA) as follows: Image -> Type –> 8 bit, next: Image -> Adjust–> Brightness/Contrast –> Auto. (p-AKT /β-actin): (AKT/β-actin) = 1:0.61.

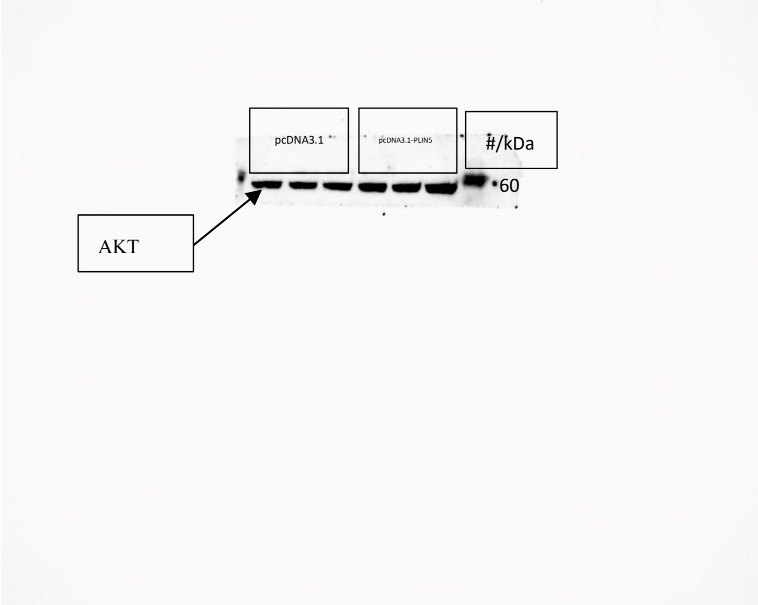

Figure 5A- Western blot membrane of AKT1 (~60 kDa) protein detected with anti-AKT1 (ab32505; 1:1000; ab32505, Cambridge, US) antibody. Gel-separated proteins were transferred

to PVDF membranes (0.45 µm pore size; Millipore, Billerice, USA) by semidry electroblotting (1.3 A,25V, 15 min). Membranes, incubated with a horseradish peroxidase-conjugated secondary antibody (BA1054; 1:5000–1:10000; Boster), were developed with Oriscience Supersensitive Kit (Oriscience Biotechnology). #Weight marker (molecular weight in kDa): Blue Plus IV Protein marker, 10 to 180 kDa; catalogue number: R10519. Blot images, prior to the densitometry readings, were converted to grayscale with ImageJ (ImageJ, National Institutes of Health, Maryland, USA) as follows: Image -> Type –> 8 bit, next: Image -> Adjust–> Brightness/Contrast –> Auto. (p-AKT /β-actin): (AKT/β-actin) = 1:0.61.

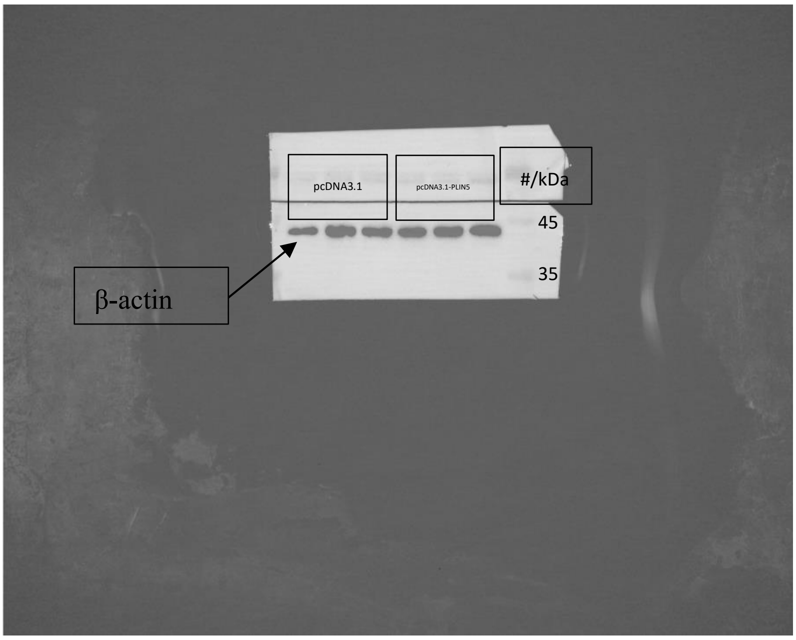

Figure 5A - Western blot membrane of β-actin (~42 kDa) protein detected with anti-β-actin (BM0627; 1:8000; Boster, Wuhan, China) antibody. Gel-separated proteins were transferred to PVDF membranes (0.45 µm pore size; Millipore, Billerice, USA) by semidry electroblotting (1.3 A ,25V, 20 min). Membranes, incubated with a horseradish peroxidase-conjugated secondary antibody (BA1050; 1:5000–1:10000; Boster), were developed with Oriscience Supersensitive Kit (Oriscience Biotechnology). #Weight marker (molecular weight in kDa): Blue Plus IV Protein marker, 10 to 180 kDa; catalogue number: R10519. Blot images, prior to the densitometry readings, were converted to grayscale with ImageJ (ImageJ, National Institutes of Health, Maryland, USA) as follows: Image -> Type –> 8 bit, next: Image -> Adjust–> Brightness/Contrast –> Auto. (p-AKT /β-actin): (AKT/β-actin) = 1:0.61.

Figure 5B- p-AKT/AKT expression level after knockdown of *PLIN5*

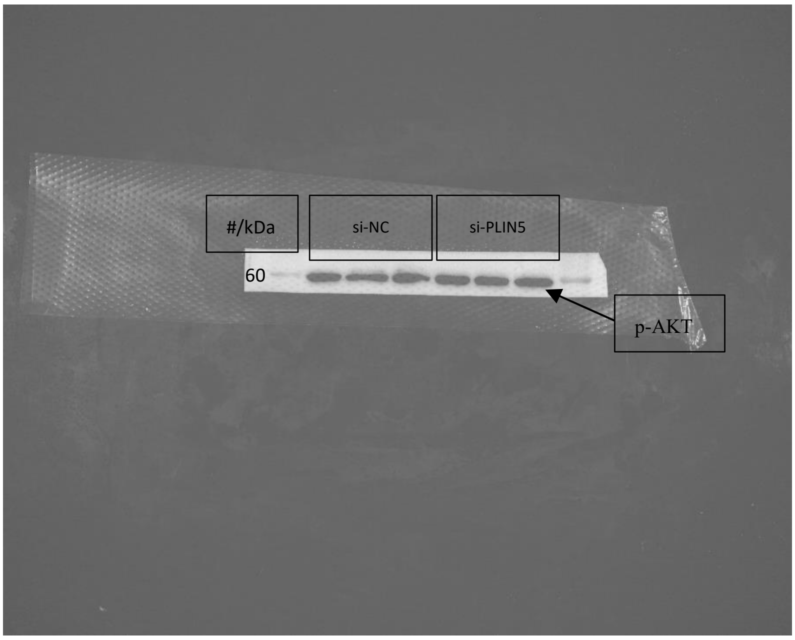

Figure 5B- Western blot membrane of p-AKT (~60 kDa) protein detected with anti-p-AKT1-Ser473 (4060; 1:2000; Cell Signaling Technology, Massachusetts, USA) antibody. Gel-separated proteins were transferred to PVDF membranes (0.45 µm pore size; Millipore, Billerice, USA) by semidry electroblotting (1.3 A,25V, 15 min). Membranes, incubated with a horseradish peroxidase-conjugated secondary antibody (BA1054; 1:5000–1:10000; Boster), were developed with Oriscience Supersensitive Kit (Oriscience Biotechnology). #Weight marker (molecular weight in kDa): Blue Plus IV Protein marker, 10 to 180 kDa; catalogue number: R10519. Blot images, prior to the densitometry readings, were converted to grayscale with ImageJ (ImageJ , National Institutes of Health, Maryland, USA) as follows: Image -> Type –> 8 bit, next: Image -> Adjust–> Brightness/Contrast –> Auto. (p-AKT /β-actin): (AKT/β-actin) = 1:1.36.

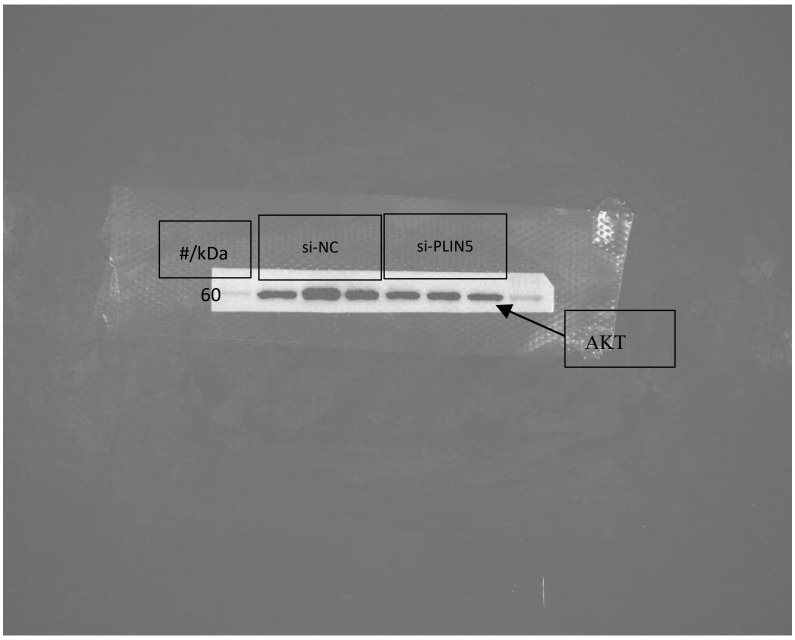

Figure 5B- Western blot membrane of AKT1 (~60 kDa) protein detected with anti-AKT1

(ab32505; 1:1000; ab32505, Cambridge, US) antibody. Gel-separated proteins were transferred to PVDF membranes (0.45 μm pore size; Millipore, Billerice, USA) by semidry electroblotting (1.3 A,2.5V, 15 min). Membranes, incubated with a horseradish peroxidase-conjugated secondary antibody (BA1054; 1:5000–1:10000; Boster), were developed with Oriscience Supersensitive Kit (Oriscience Biotechnology). #Weight marker (molecular weight in kDa): Blue Plus IV Protein marker, 10 to 180 kDa; catalogue number: R10519. Blot images, prior to the densitometry readings, were converted to grayscale with ImageJ (ImageJ, National Institutes of Health, Maryland, USA) as follows: Image -> Type –> 8 bit, next: Image -> Adjust–> Brightness/Contrast –> Auto. (p-AKT /β-actin): (AKT/β-actin) = 1:0.61.

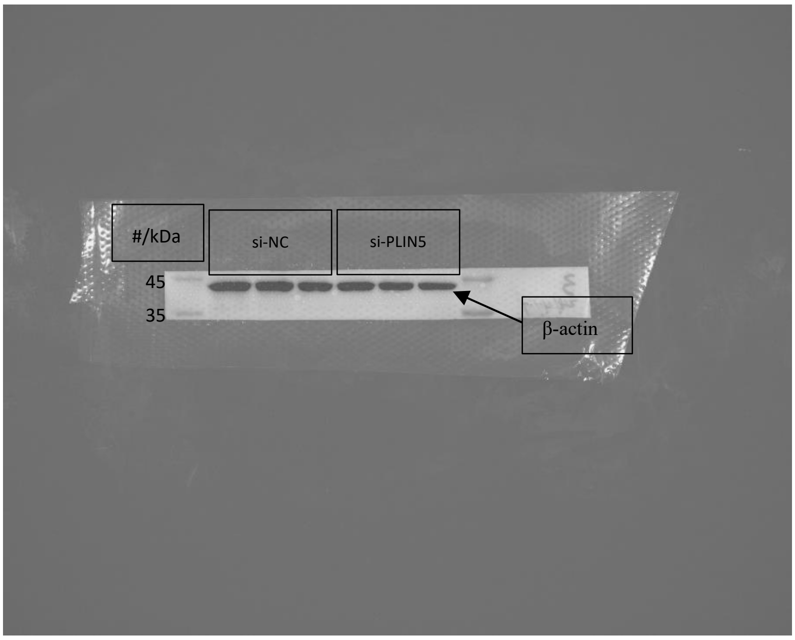

Figure 5B - Western blot membrane of β-actin (~42 kDa) protein detected with anti-β-actin (BM0627; 1:8000; Boster, Wuhan, China) antibody. Gel-separated proteins were transferred to PVDF membranes (0.45 μm pore size; Millipore, Billerice, USA) by semidry electroblotting (1.3 A ,2.5V, 20 min). Membranes, incubated with a horseradish peroxidase-conjugated secondary antibody (BA1050; 1:5000–1:10000; Boster), were developed with Oriscience Supersensitive Kit (Oriscience Biotechnology). #Weight marker (molecular weight in kDa): Blue Plus IV Protein marker, 10 to 180 kDa; catalogue number: R10519. Blot images, prior to the densitometry readings, were converted to grayscale with ImageJ (ImageJ, National Institutes of Health, Maryland, USA) as follows: Image -> Type –> 8 bit, next: Image -> Adjust–> Brightness/Contrast –> Auto. (p-AKT /β-actin): (AKT/β-actin) = 1:0.61.

Figure 5C -p-AKT/AKT expression level after adding LY294002

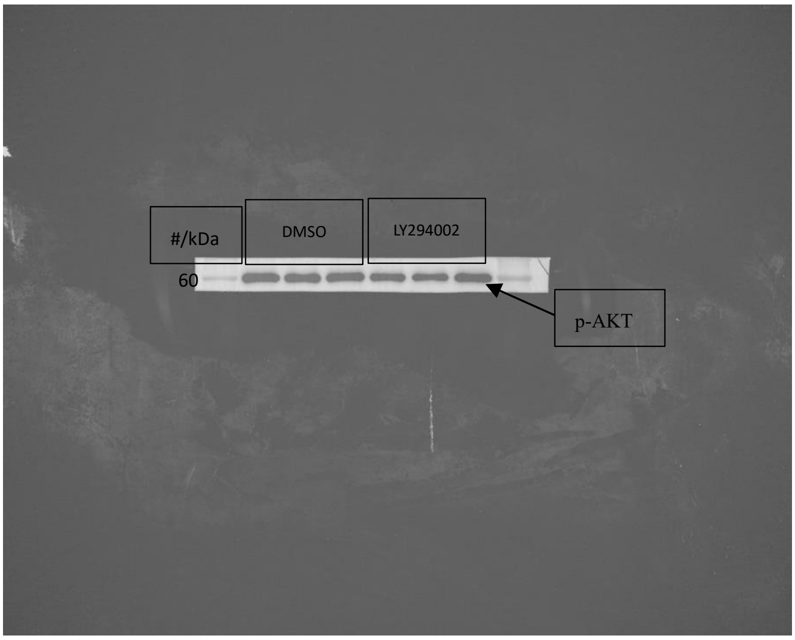

Figure 5C- Western blot membrane of p-AKT (~60 kDa) protein detected with anti-p-AKT1-Ser473 (4060; 1:2000; Cell Signaling Technology, Massachusetts, USA) antibody. Gel-separated proteins were transferred to PVDF membranes (0.45 µm pore size; Millipore, Billerice, USA) by semidry electroblotting (1.3 A,2.5V, 15 min). Membranes, incubated with a horseradish peroxidase-conjugated secondary antibody (BA1054; 1:5000–1:10000; Boster), were developed with Oriscience Supersensitive Kit (Oriscience Biotechnology). #Weight marker (molecular weight in kDa): Blue Plus IV Protein marker, 10 to 180 kDa; catalogue number: R10519. Blot images, prior to the densitometry readings, were converted to grayscale with ImageJ (ImageJ, National Institutes of Health, Maryland, USA) as follows: Image -> Type –> 8 bit, next: Image -> Adjust–> Brightness/Contrast –> Auto. (p-AKT /β-actin): (AKT/β-actin) = 1:0.75.

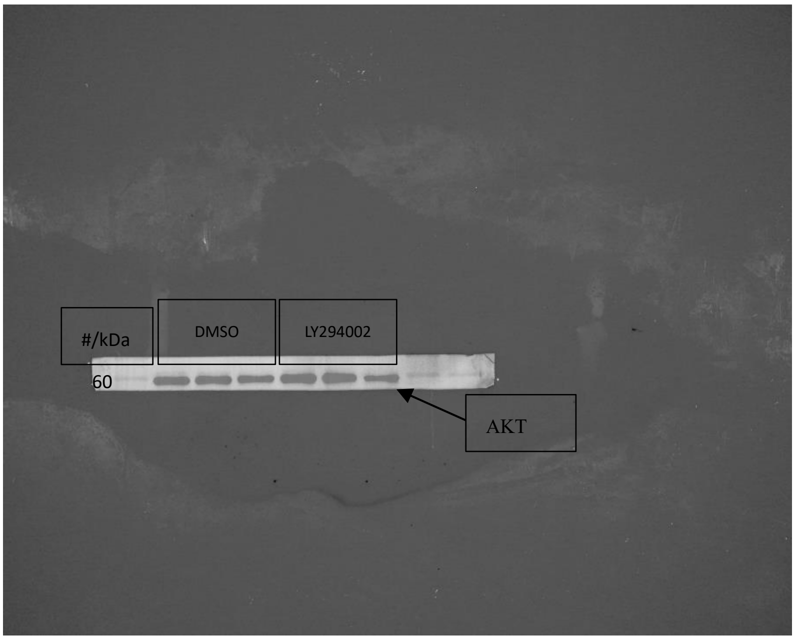

Figure 5C- Western blot membrane of AKT1 (~60 kDa) protein detected with anti-AKT1 (ab32505; 1:1000; ab32505, Cambridge, US) antibody. Gel-separated proteins were transferred to PVDF membranes (0.45 µm pore size; Millipore, Billerice, USA) by semidry electroblotting (1.3 A,2.5V, 15 min). Membranes, incubated with a horseradish peroxidase-conjugated secondary antibody (BA1054; 1:5000–1:10000; Boster), were developed with Oriscience Supersensitive Kit (Oriscience Biotechnology). #Weight marker (molecular weight in kDa): Blue Plus IV Protein marker, 10 to 180 kDa; catalogue number: R10519. Blot images, prior to the densitometry readings, were converted to grayscale with ImageJ (ImageJ, National Institutes of Health, Maryland, USA) as follows: Image -> Type –> 8 bit, next: Image -> Adjust–> Brightness/Contrast –> Auto. (p-AKT /β-actin): (AKT/β-actin) = 1:0.75.

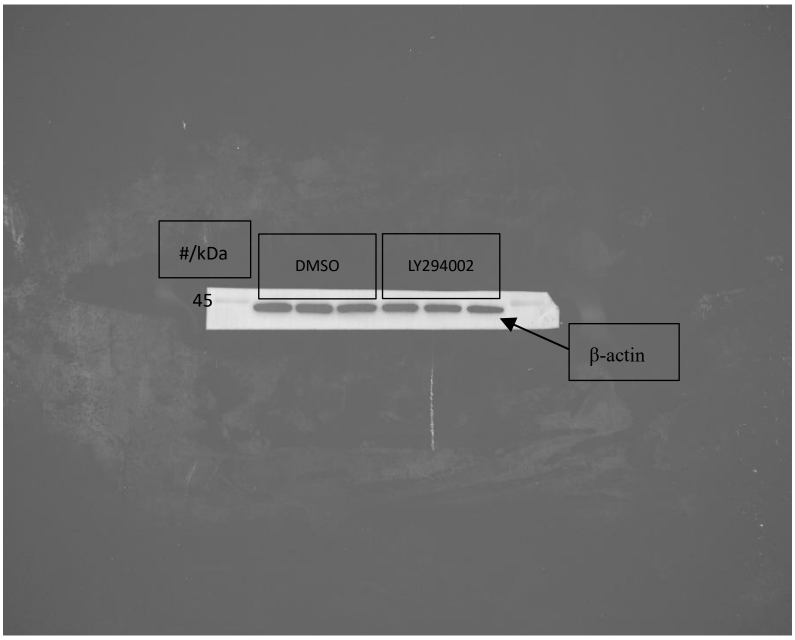

Figure 5C - Western blot membrane of β-actin (~42 kDa) protein detected with anti-β-actin (BM0627; 1:8000; Boster, Wuhan, China) antibody. Gel-separated proteins were transferred to PVDF membranes (0.45 µm pore size; Millipore, Billerice, USA) by semidry electroblotting (1.3 A ,2.5V, 20 min). Membranes, incubated with a horseradish peroxidase-conjugated secondary antibody (BA1050; 1:5000–1:10000; Boster), were developed with Oriscience Supersensitive Kit (Oriscience Biotechnology). #Weight marker (molecular weight in kDa): Blue Plus IV Protein marker, 10 to 180 kDa; catalogue number: R10519. Blot images, prior to the densitometry readings, were converted to grayscale with ImageJ (ImageJ, National Institutes of Health, Maryland, USA) as follows: Image -> Type –> 8 bit, next: Image -> Adjust–> Brightness/Contrast –> Auto. (p-AKT /β-actin): (AKT/β-actin) = 1:0.75.

Figure 6B- *PPARγ* expression level after overexpression of *PLIN5*

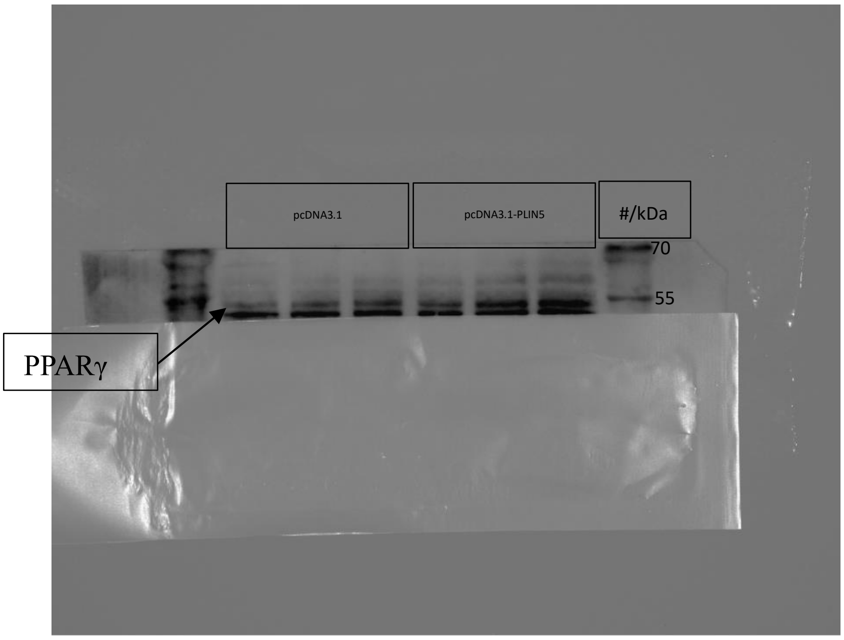

Figure 6B- Western blot membrane of PPARγ (~54 kDa) protein detected with anti- PPARγ (16643-1-Ap; 1:8000; Proteintech, Wuhan, China) antibody. Gel-separated proteins were transferred to PVDF membranes (0.45 µm pore size; Millipore, Billerice, USA) by semidry electroblotting (1.3 A,2.5V, 15 min). Membranes, incubated with a horseradish peroxidase-conjugated secondary antibody (BA1054; 1:5000–1:10000; Boater), were developed with Oriscience Supersensitive Kit (Oriscience Biotechnology). #Weight marker (molecular weight in kDa): Blue Plus IV Protein marker, 10 to 180 kDa; catalogue number: R21223-V2. Blot images, prior to the densitometry readings, were converted to grayscale with ImageJ (ImageJ, National Institutes of Health, Maryland, USA) as follows: Image -> Type –> 8 bit, next: Image -> Adjust–> Brightness/Contrast –> Auto. pcDNA3.1(PPARγ/β-actin): pcDNA3.1-*PLIN5* (PPARγ/β-actin) = 1:1.60.

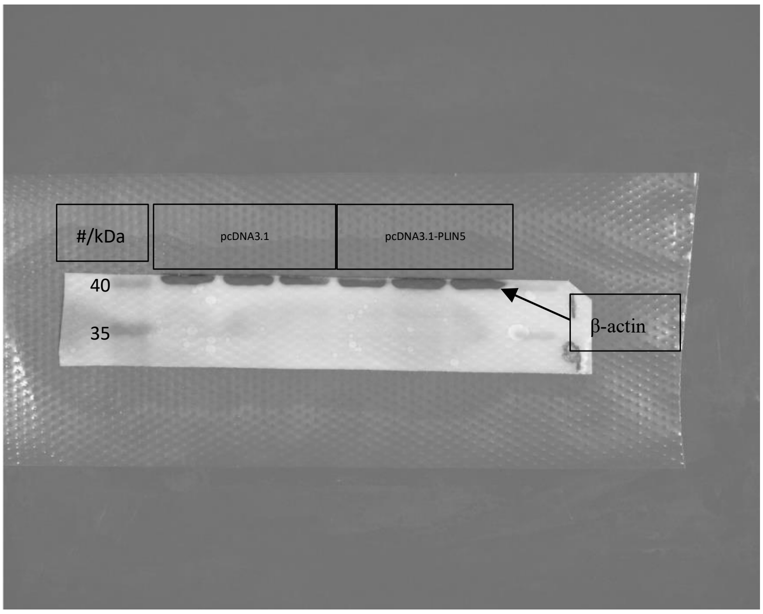

Figure 6B - Western blot membrane of β-actin (~42 kDa) protein detected with anti-β-actin

(BM0627; 1:8000; Boster, Wuhan, China) antibody. Gel-separated proteins were transferred to PVDF membranes (0.45 µm pore size; Millipore, Billerice, USA) by semidry electroblotting (1.3 A ,2.5V, 20 min). Membranes, incubated with a horseradish peroxidase-conjugated secondary antibody (BA1050; 1:5000–1:10000; Boster), were developed with Oriscience Supersensitive Kit (Oriscience Biotechnology). #Weight marker (molecular weight in kDa): Blue Plus IV Protein marker, 10 to 180 kDa; catalogue number: R21223-V2. Blot images, prior to the densitometry readings, were converted to grayscale with ImageJ (ImageJ, National Institutes of Health, Maryland, USA) as follows: Image -> Type –> 8 bit, next: Image -> Adjust–> Brightness/Contrast –> Auto. pcDNA3.1(PPARγ/β-actin): pcDNA3.1-*PLIN5* (PPARγ/β-actin) = 1:1.60.

Figure 6B- *PPARγ* expression level after knockdown of *PLIN5*

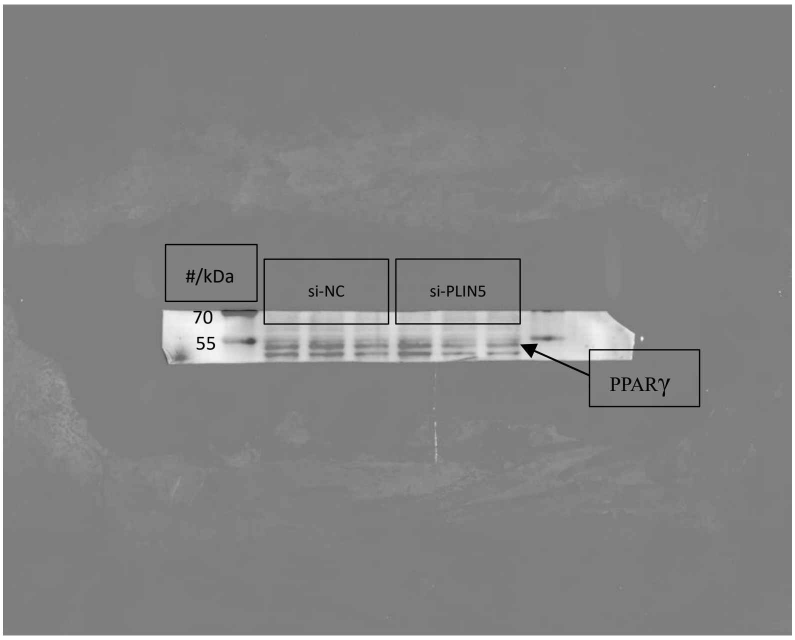

Figure 6B- Western blot membrane of PPARγ (~54 kDa) protein detected with anti- PPARγ (16643-1-Ap; 1:8000; Proteintech, Wuhan, China) antibody. Gel-separated proteins were transferred to PVDF membranes (0.45 µm pore size; Millipore, Billerice, USA) by semidry electroblotting (1.3 A,2.5V, 15 min). Membranes, incubated with a horseradish peroxidase-conjugated secondary antibody (BA1054; 1:5000–1:10000; Boater), were developed with Oriscience Supersensitive Kit (Oriscience Biotechnology). #Weight marker (molecular weight in kDa): Blue Plus IV Protein marker, 10 to 180 kDa; catalogue number: R21223-V2. Blot images, prior to the densitometry readings, were converted to grayscale with ImageJ (ImageJ, National Institutes of Health, Maryland, USA) as follows: Image -> Type –> 8 bit, next: Image -> Adjust–> Brightness/Contrast –> Auto. Si-*NC*(PPARγ/β-actin): si-*PLIN5* (PPARγ/β-actin) = 1:0.68.

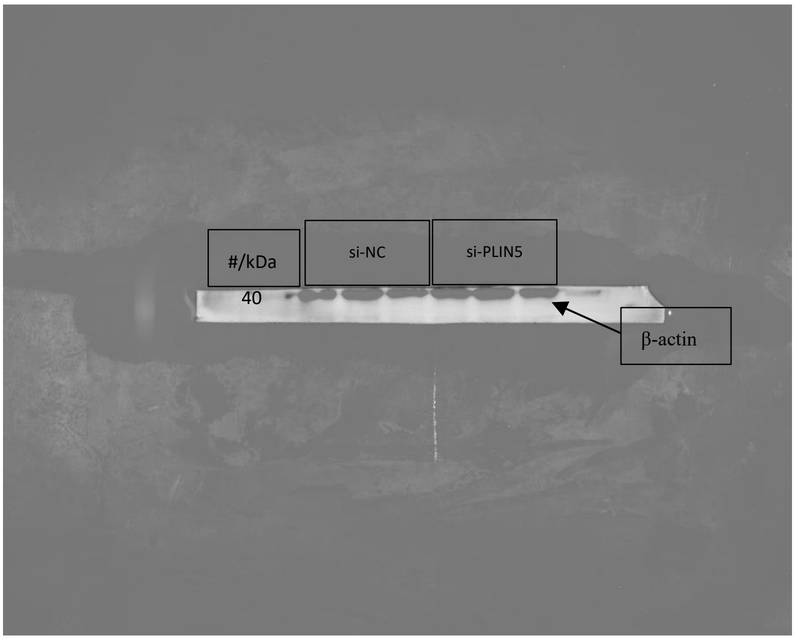

Figure 6B - Western blot membrane of β-actin (~42 kDa) protein detected with anti-β-actin (BM0627; 1:8000; Boster, Wuhan, China) antibody. Gel-separated proteins were transferred to PVDF membranes (0.45 µm pore size; Millipore, Billerice, USA) by semidry electroblotting (1.3 A ,2.5V, 20 min). Membranes, incubated with a horseradish peroxidase-conjugated secondary antibody (BA1050; 1:5000–1:10000; Boster), were developed with Oriscience Supersensitive Kit (Oriscience Biotechnology). #Weight marker (molecular weight in kDa): Blue Plus IV Protein marker, 10 to 180 kDa; catalogue number: R21223-V2. Blot images, prior to the densitometry readings, were converted to grayscale with ImageJ (ImageJ, National Institutes of Health, Maryland, USA) as follows: Image -> Type –> 8 bit, next: Image -> Adjust–> Brightness/Contrast –> Auto. Si-*NC*(PPARγ/β-actin): si-*PLIN5* (PPARγ/β-actin) = 1:0.68

Figure 7A- *PPARγ* expression level after knockdown of *PPARγ*

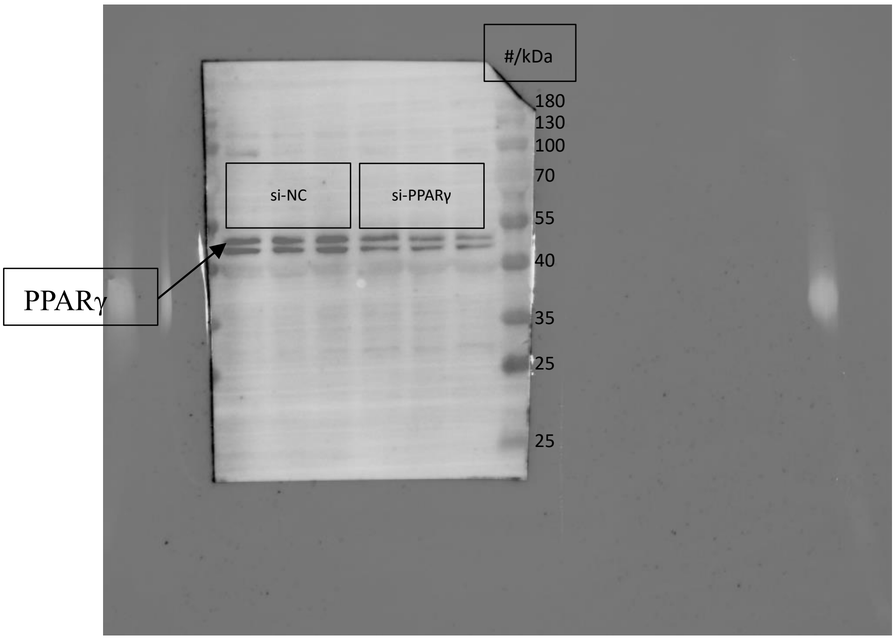

Figure 7A- Western blot membrane of PPARγ (~54 kDa) protein detected with anti- PPARγ

(16643-1-Ap; 1:8000; Proteintech, Wuhan, China) antibody. Gel-separated proteins were transferred to PVDF membranes (0.45 μm pore size; Millipore, Billerice, USA) by semidry electroblotting (1.3 A,2.5V, 15 min). Membranes, incubated with a horseradish peroxidase-conjugated secondary antibody (BA1054; 1:5000–1:10000; Boater), were developed with Oriscience Supersensitive Kit (Oriscience Biotechnology). #Weight marker (molecular weight in kDa): Blue Plus IV Protein marker, 10 to 180 kDa; catalogue number: R21223-V2. Blot images, prior to the densitometry readings, were converted to grayscale with ImageJ (ImageJ, National Institutes of Health, Maryland, USA) as follows: Image -> Type –> 8 bit, next: Image -> Adjust–> Brightness/Contrast –> Auto. (Si-*NC*/β-actin): (si- *PPARγ*/β-actin) = 1:0.66.

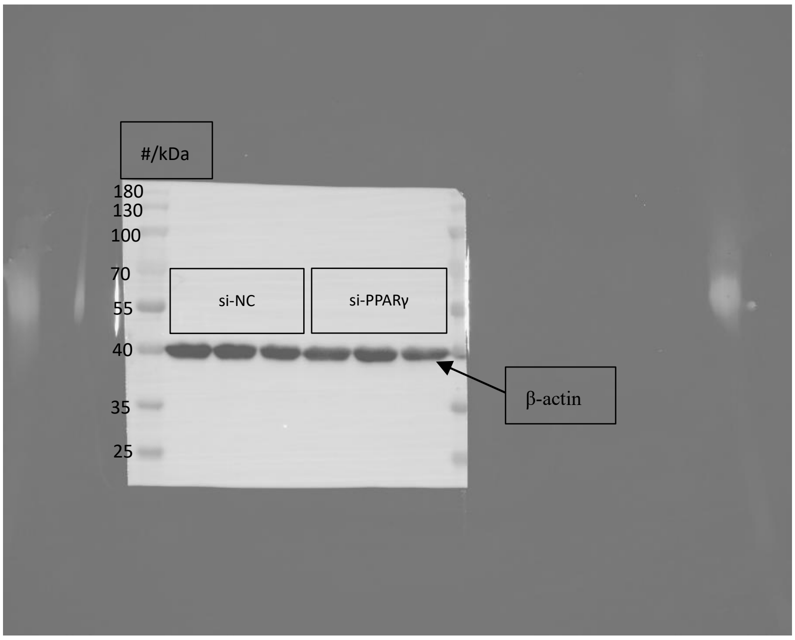

Figure 7A - Western blot membrane of β-actin (~42 kDa) protein detected with anti-β-actin (BM0627; 1:8000; Boster, Wuhan, China) antibody. Gel-separated proteins were transferred to PVDF membranes (0.45 μm pore size; Millipore, Billerice, USA) by semidry electroblotting (1.3 A ,2.5V, 20 min). Membranes, incubated with a horseradish peroxidase-conjugated secondary antibody (BA1050; 1:5000–1:10000; Boster), were developed with Oriscience Supersensitive Kit (Oriscience Biotechnology). #Weight marker (molecular weight in kDa): Blue Plus IV Protein marker, 10 to 180 kDa; catalogue number: R21223-V2. Blot images, prior to the densitometry readings, were converted to grayscale with ImageJ (ImageJ, National Institutes of Health, Maryland, USA) as follows: Image -> Type –> 8 bit, next: Image -> Adjust–> Brightness/Contrast –> Auto. (Si-*NC*/β-actin): (si- *PPARγ*/β-actin) = 1:0.66.

Figure 7H- p-AKT/AKT expression level after adding GW9662

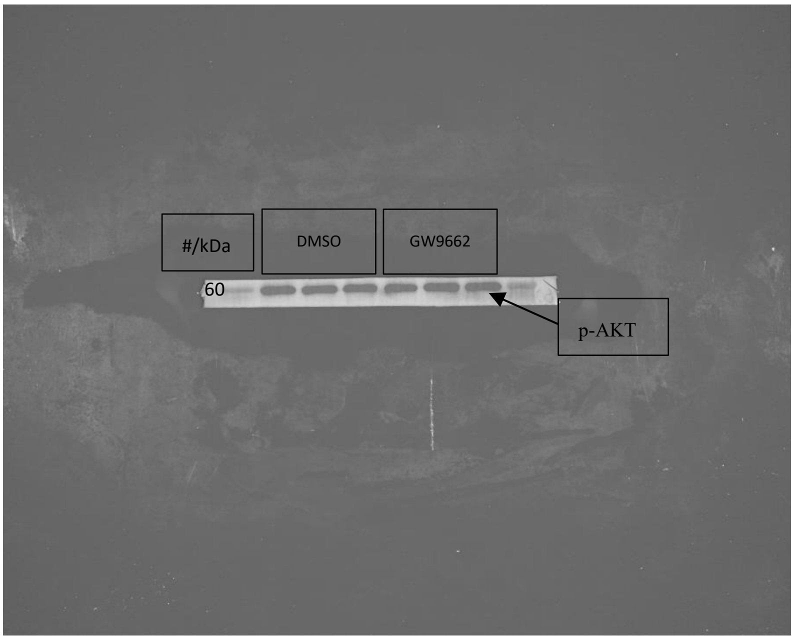

Figure 7H- Western blot membrane of p-AKT (~60 kDa) protein detected with anti-p-AKT1-Ser473 (4060; 1:2000; Cell Signaling Technology, Massachusetts, USA) antibody. Gel-separated proteins were transferred to PVDF membranes (0.45 µm pore size; Millipore, Billerice, USA) by semidry electroblotting (1.3 A,2.5V, 15 min). Membranes, incubated with a horseradish peroxidase-conjugated secondary antibody (BA1054; 1:5000–1:10000; Boster), were developed with Oriscience Supersensitive Kit (Oriscience Biotechnology). #Weight marker (molecular weight in kDa): Blue Plus IV Protein marker, 10 to 180 kDa; catalogue number: R10519. Blot images, prior to the densitometry readings, were converted to grayscale with ImageJ (ImageJ, National Institutes of Health, Maryland, USA) as follows: Image -> Type –> 8 bit, next: Image -> Adjust–> Brightness/Contrast –> Auto. DMSO (p-AKT /β-actin): GW9662(p-AKT/β-actin) = 1:1.06.

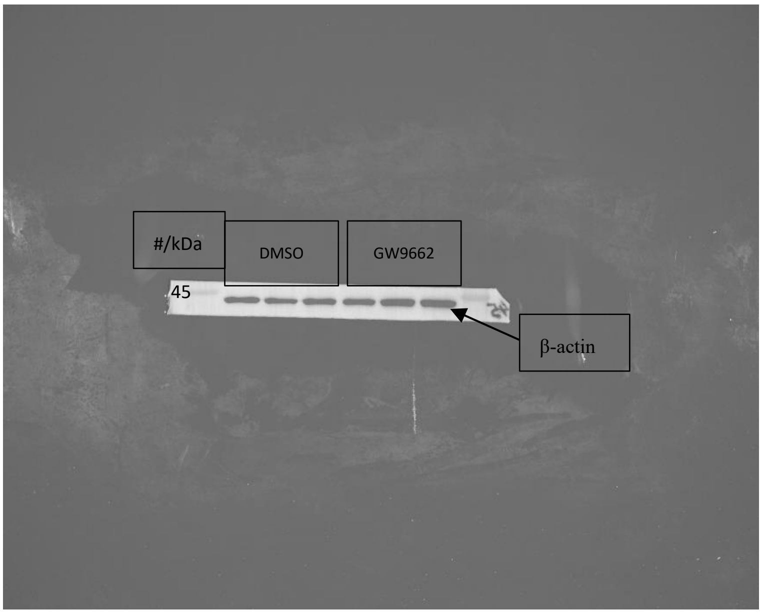

Figure 7H - Western blot membrane of β-actin (~42 kDa) protein detected with anti-β-actin

(BM0627; 1:8000; Boster, Wuhan, China) antibody. Gel-separated proteins were transferred to PVDF membranes (0.45 μm pore size; Millipore, Billerice, USA) by semidry electroblotting (1.3 A ,2.5V, 20 min). Membranes, incubated with a horseradish peroxidase-conjugated secondary antibody (BA1050; 1:5000–1:10000; Boster), were developed with Oriscience Supersensitive Kit (Oriscience Biotechnology). #Weight marker (molecular weight in kDa): Blue Plus IV Protein marker, 10 to 180 kDa; catalogue number: R10519. Blot images, prior to the densitometry readings, were converted to grayscale with ImageJ (ImageJ, National Institutes of Health, Maryland, USA) as follows: Image -> Type –> 8 bit, next: Image -> Adjust–> Brightness/Contrast –> Auto. DMSO (p-AKT /β-actin): GW9662(p-AKT/β-actin) = 1:1.06.

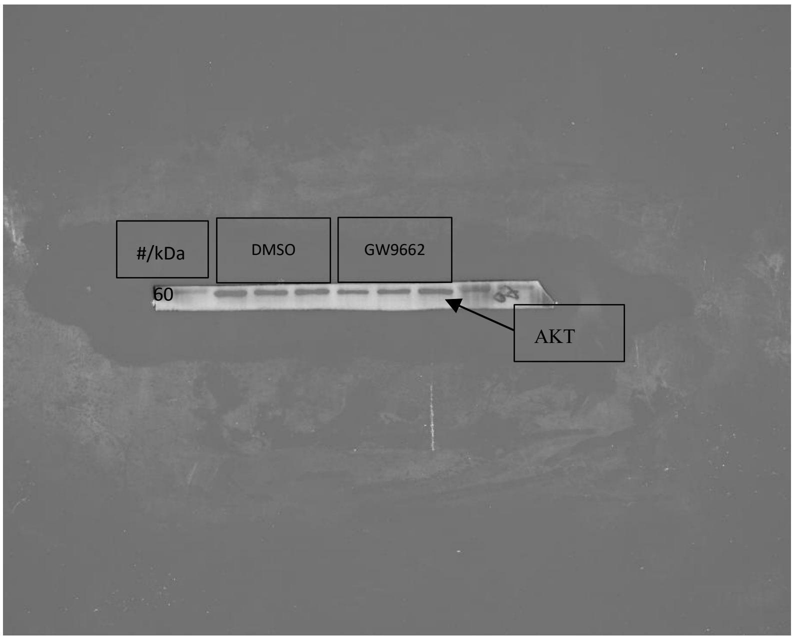

Figure 7H- Western blot membrane of AKT1 (~60 kDa) protein detected with anti-AKT1 (ab32505; 1:1000; ab32505, Cambridge, US) antibody. Gel-separated proteins were transferred to PVDF membranes (0.45 μm pore size; Millipore, Billerice, USA) by semidry electroblotting (1.3 A,2.5V, 15 min). Membranes, incubated with a horseradish peroxidase-conjugated secondary antibody (BA1054; 1:5000–1:10000; Boster), were developed with Oriscience Supersensitive Kit (Oriscience Biotechnology). #Weight marker (molecular weight in kDa): Blue Plus IV Protein marker, 10 to 180 kDa; catalogue number: R10519. Blot images, prior to the densitometry readings, were converted to grayscale with ImageJ (ImageJ, National Institutes of Health, Maryland, USA) as follows: Image -> Type –> 8 bit, next: Image -> Adjust–> Brightness/Contrast –> Auto. DMSO (AKT /β-actin): GW9662(AKT/β-actin) = 1:0.71.

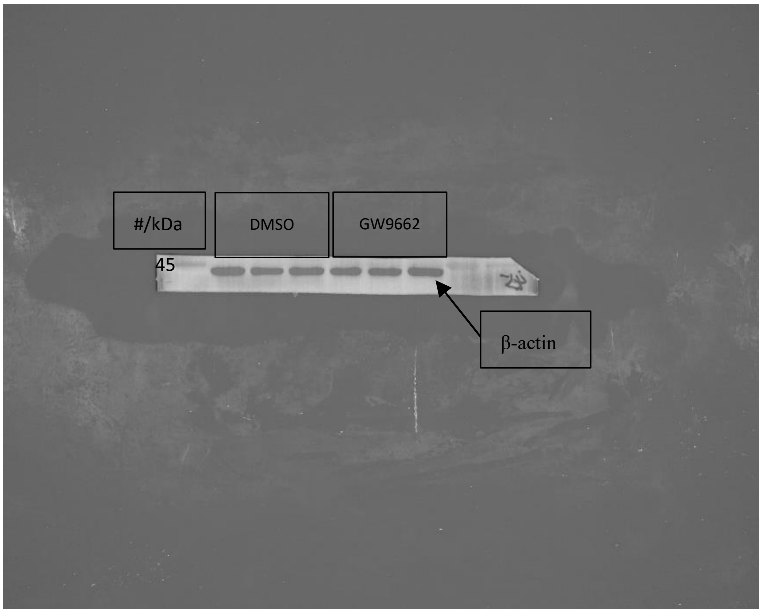

Figure 7H - Western blot membrane of β-actin (~42 kDa) protein detected with anti-β-actin (BM0627; 1:8000; Boster, Wuhan, China) antibody. Gel-separated proteins were transferred to PVDF membranes (0.45 μm pore size; Millipore, Billerice, USA) by semidry electroblotting (1.3 A ,2.5V, 20 min). Membranes, incubated with a horseradish peroxidase-conjugated secondary antibody (BA1050; 1:5000–1:10000; Boster), were developed with Oriscience Supersensitive Kit (Oriscience Biotechnology). #Weight marker (molecular weight in kDa): Blue Plus IV Protein marker, 10 to 180 kDa; catalogue number: R10519. Blot images, prior to the densitometry readings, were converted to grayscale with ImageJ (ImageJ, National Institutes of Health, Maryland, USA) as follows: Image -> Type –> 8 bit, next: Image -> Adjust–> Brightness/Contrast –> Auto. DMSO (AKT /β-actin): GW9662(AKT/β-actin) = 1:0.71.

Figure S3C-p-p38/p38 expression level after overexpression of *PLIN5*

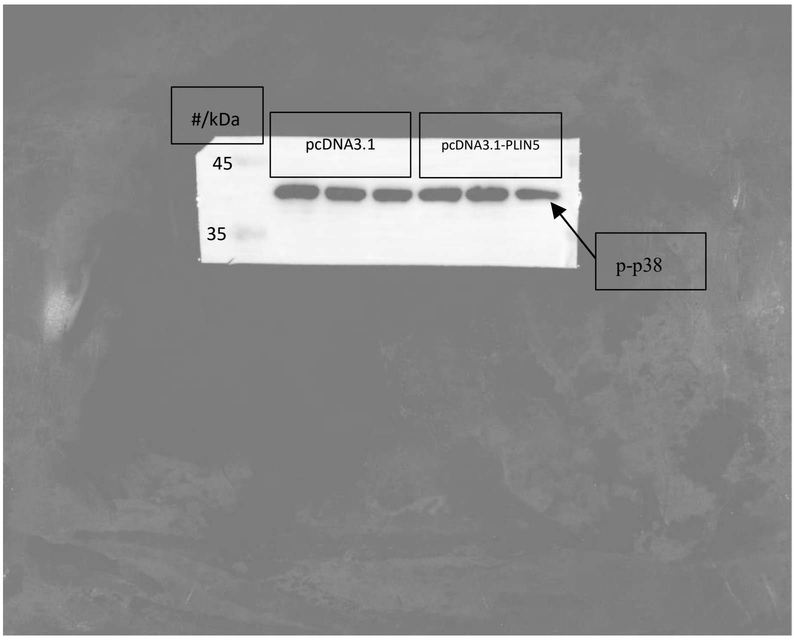

Figure S3C - Western blot membrane of p-p38 (~43 kDa) protein detected with anti- p-p38-MAPK (3285S; 1:1000; Cell Signaling Technology, Massachusetts, USA) antibody. Gel-

separated proteins were transferred to PVDF membranes (0.45 μm pore size; Millipore, Billerice, USA) by semidry electroblotting (1.3 A ,2.5V, 15 min). Membranes, incubated with a horseradish peroxidase-conjugated secondary antibody (BA1050; 1:5000–1:10000; Boster), were developed with Oriscience Supersensitive Kit (Oriscience Biotechnology). #Weight marker (molecular weight in kDa): Blue Plus IV Protein marker, 10 to 180 kDa; catalogue number: R10519. Blot images, prior to the densitometry readings, were converted to grayscale with ImageJ (ImageJ, National Institutes of Health, Maryland, USA) as follows: Image -> Type –> 8 bit, next: Image -> Adjust–> Brightness/Contrast –> Auto. (p-p38 /β-actin): (p38/β-actin) = 1:0.89.

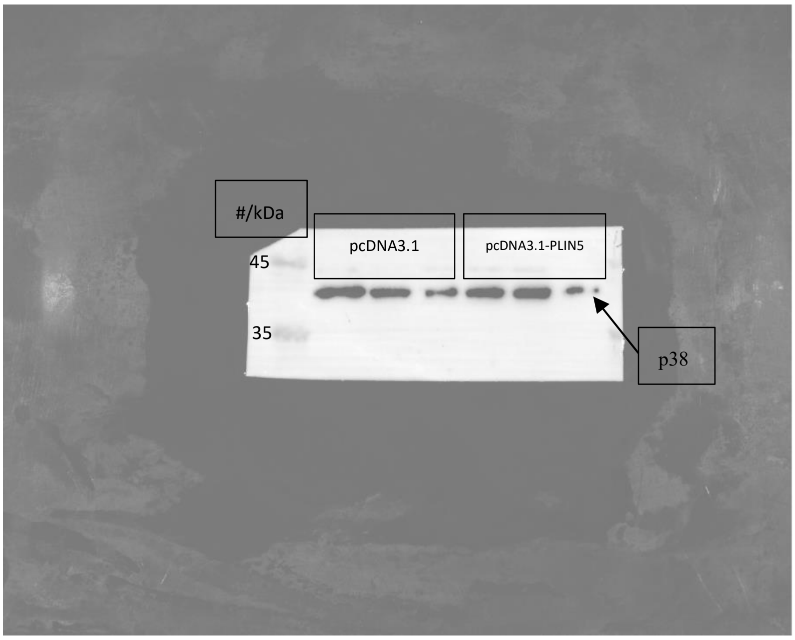

Figure S3C - Western blot membrane of p38 (~40 kDa) protein detected with anti- p38-MAPK (4; 1:1000; Cell Signaling Technology, Massachusetts, USA) antibody. Gel-separated proteins were transferred to PVDF membranes (0.45 μm pore size; Millipore, Billerice, USA) by semidry electroblotting (1.3 A ,2.5V, 15 min). Membranes, incubated with a horseradish peroxidase-conjugated secondary antibody (BA1050; 1:5000–1:10000; Boster), were developed with Oriscience Supersensitive Kit (Oriscience Biotechnology). #Weight marker (molecular weight in kDa): Blue Plus IV Protein marker, 10 to 180 kDa; catalogue number: R10519. Blot images, prior to the densitometry readings, were converted to grayscale with ImageJ (ImageJ, National Institutes of Health, Maryland, USA) as follows: Image -> Type –> 8 bit, next: Image -> Adjust–> Brightness/Contrast –> Auto. (p-p38 /β-actin): (p-38/β-actin) = 1:0.89.

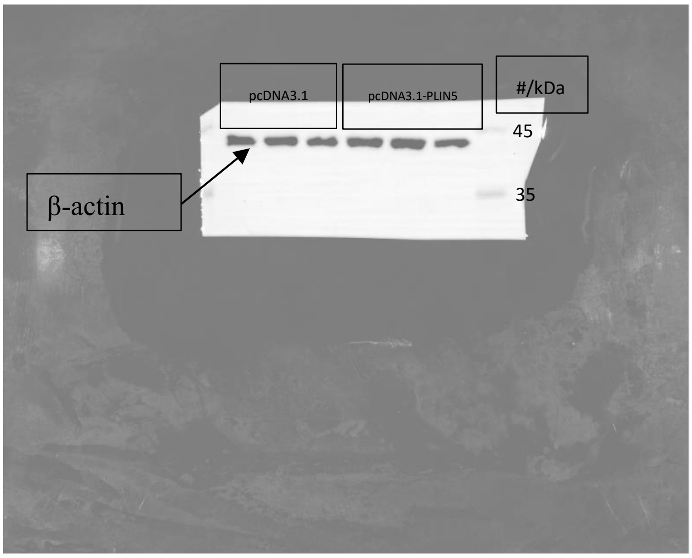

Figure S3C - Western blot membrane of β-actin (~42 kDa) protein detected with anti-β-actin (BM0627; 1:8000; Boster, Wuhan, China) antibody. Gel-separated proteins were transferred to PVDF membranes (0.45 µm pore size; Millipore, Billerice, USA) by semidry electroblotting (1.3 A ,2.5V, 20 min). Membranes, incubated with a horseradish peroxidase-conjugated secondary antibody (BA1050; 1:5000–1:10000; Boster), were developed with Oriscience Supersensitive Kit (Oriscience Biotechnology). #Weight marker (molecular weight in kDa): Blue Plus IV Protein marker, 10 to 180 kDa; catalogue number: R10519. Blot images, prior to the densitometry readings, were converted to grayscale with ImageJ (ImageJ, National Institutes of Health, Maryland, USA) as follows: Image -> Type –> 8 bit, next: Image -> Adjust–> Brightness/Contrast –> Auto. (p-p38 /β-actin): (p-38/β-actin) = 1:0.89.

Figure S5C- p-AKT/AKT expression level after knockdown of *PPARγ*

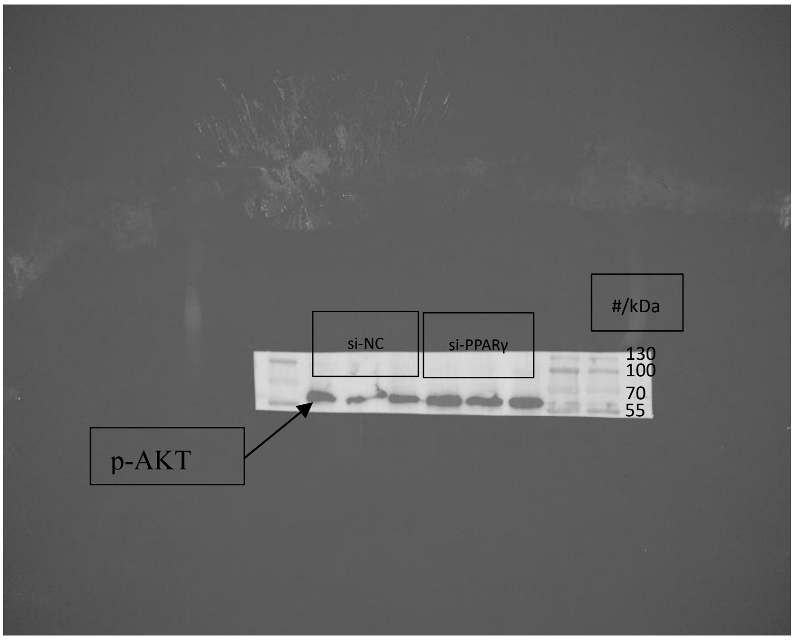

Figure S5C - Western blot membrane of p-AKT (~60 kDa) protein detected with anti-p-AKT1-Ser473 (4060; 1:2000; Cell Signaling Technology, Massachusetts, USA) antibody. Gel-separated proteins were transferred to PVDF membranes (0.45 µm pore size; Millipore, Billerice, USA) by

semidry electroblotting (1.3 A,2.5V, 15 min). Membranes, incubated with a horseradish peroxidase-conjugated secondary antibody (BA1054; 1:5000–1:10000; Boater), were developed with Oriscience Supersensitive Kit (Oriscience Biotechnology). #Weight marker (molecular weight in kDa): Blue Plus IV Protein marker, 10 to 180 kDa; catalogue number: R21223-V2. Blot images, prior to the densitometry readings, were converted to grayscale with ImageJ (ImageJ, National Institutes of Health, Maryland, USA) as follows: Image -> Type –> 8 bit, next: Image -> Adjust–> Brightness/Contrast –> Auto. （p-AKT/β-actin): (AKT/β-actin) = 1:1.54.

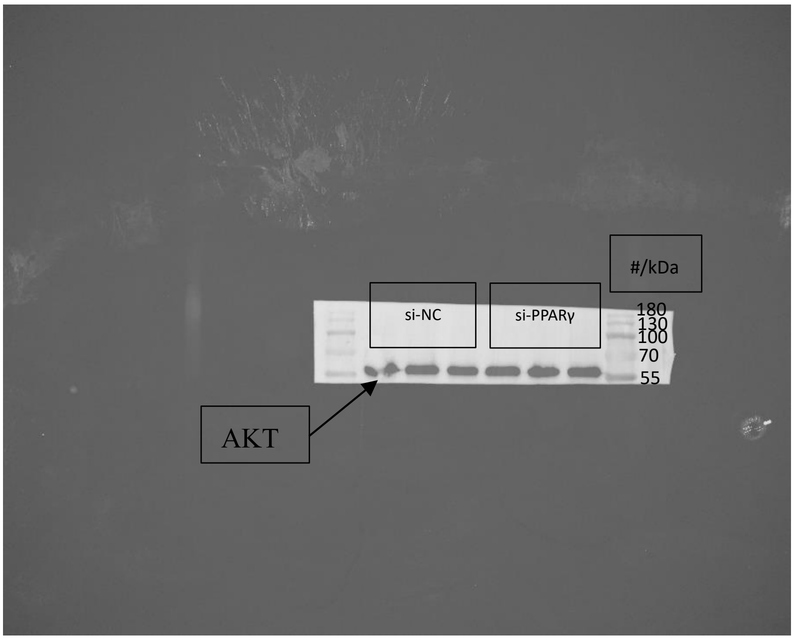

Figure S5C - Western blot membrane of AKT1 (~60 kDa) protein detected with anti-AKT1 (ab32505; 1:1000; ab32505, Cambridge, US) antibody. Gel-separated proteins were transferred to PVDF membranes (0.45 µm pore size; Millipore, Billerice, USA) by semidry electroblotting (1.3 A,2.5V 2, 15 min). Membranes, incubated with a horseradish peroxidase-conjugated secondary antibody (BA1054; 1:5000–1:10000; Boster), were developed with Oriscience Supersensitive Kit (Oriscience Biotechnology). #Weight marker (molecular weight in kDa): Blue Plus IV Protein marker, 10 to 180 kDa; catalogue number: R21223-V2. Blot images, prior to the densitometry readings, were converted to grayscale with ImageJ (ImageJ, National Institutes of Health, Maryland, USA) as follows: Image -> Type –> 8 bit, next: Image -> Adjust–> Brightness/Contrast –> Auto. （p-AKT/β-actin): (AKT/β-actin) = 1:1.54.

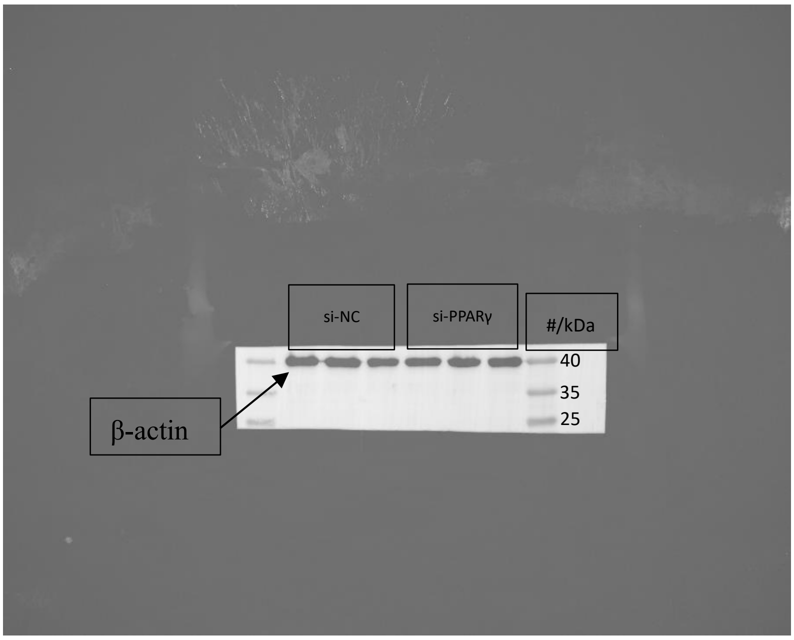

Figure S3C - Western blot membrane of β-actin (~42 kDa) protein detected with anti-β-actin (BM0627; 1:8000; Boster, Wuhan, China) antibody. Gel-separated proteins were transferred to PVDF membranes (0.45 µm pore size; Millipore, Billerice, USA) by semidry electroblotting (1.3 A ,2.5V, 20 min). Membranes, incubated with a horseradish peroxidase-conjugated secondary antibody (BA1050; 1:5000–1:10000; Boster), were developed with Oriscience Supersensitive Kit (Oriscience Biotechnology). #Weight marker (molecular weight in kDa): Blue Plus IV Protein marker, 10 to 180 kDa; catalogue number: R21223-V2. Blot images, prior to the densitometry readings, were converted to grayscale with ImageJ (ImageJ , National Institutes of Health, Maryland, USA) as follows: Image -> Type –> 8 bit, next: Image -> Adjust–> Brightness/Contrast –> Auto.（p-AKT/β-actin): (AKT/β-actin) = 1:1.54.

Figure S1A-

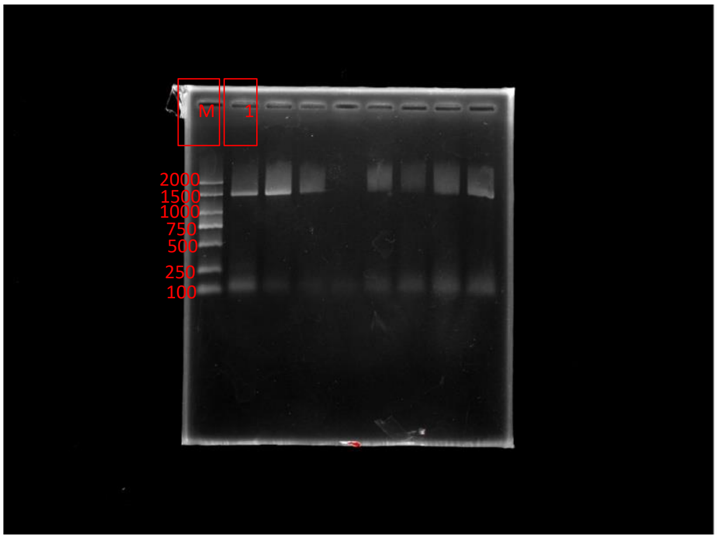

Electrophoretic analysis of the cloned goat *PLIN5* gene. Lane M: DL 2000 plus DNA Marker(MD101-02-AA,Vazyme,NanJing,China); Lane 1: PCR product of the *PLIN5* gene(~1517bp).
