# Supplementary material for: PLIN5 Promotes Lipid Reconstitution in Goat Intramuscular Fat via the PPARγ Signaling Pathway
Source: Biology (Basel). 2025 Nov 4;14(11):1547. doi: 10.3390/biology14111547 (PMC12649955; doi:10.3390/biology14111547)

The PPARγ signaling pathway is essential for PLIN5regulating lipid reconstitution in goat intramuscular fat
Manuscript Number: biology-3865461

Uncropped, full-length Western blot Images

The original, uncropped and unadjusted western blot images of protein in our manuscript are displayed as follows.

In our study, we needed to detect 8 proteins (PLIN5, PPARγ, Caspase3, p-AKT, AKT, p-P38, P38 and β-actin). We have also showed the positions of the bands and the molecular weight markers.

1、**Figure-1C**-Expression pattern of PLIN5 during differentiation of goat preadipocytes

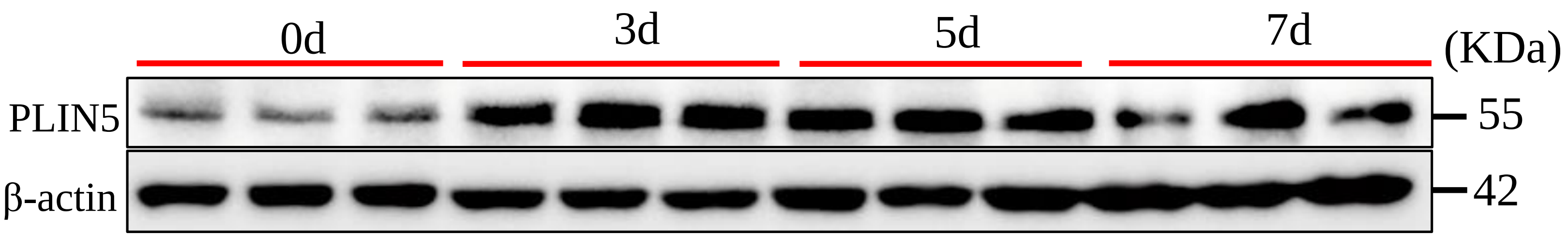

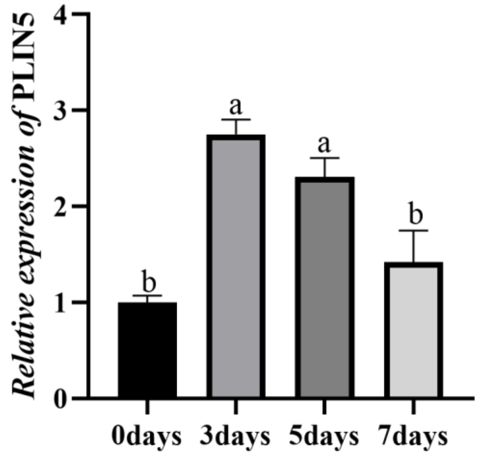

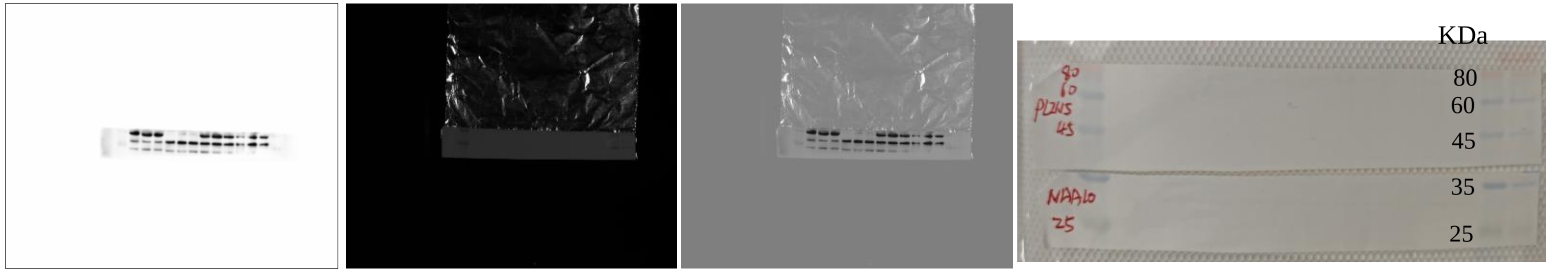

Western blot membrane of **PLIN5 (~55 kDa)** protein detected with anti-PLIN5 (A25598; 1:1000; ABclonal, Wuhan, China) antibody. Gel-separated proteins were transferred to PVDF membranes (0.45 μm pore size; Millipore, Billerice, USA) by semidry electroblotting (1.3 A,2.5V, 15 min). Membranes, incubated with a horseradish peroxidase-conjugated secondary antibody (BA1054; 1:5000–1:10000; Boater), were developed with Oriscience Supersensitive Kit (Oriscience Biotechnology). #Weight marker (molecular weight in kDa): Blue Plus IV Protein marker, 10 to 180 kDa; catalogue number: **R21223-V2**. Blot images, prior to the densitometry readings, were converted to grayscale with ImageJ (ImageJ, National Institutes of Health, Maryland, USA).

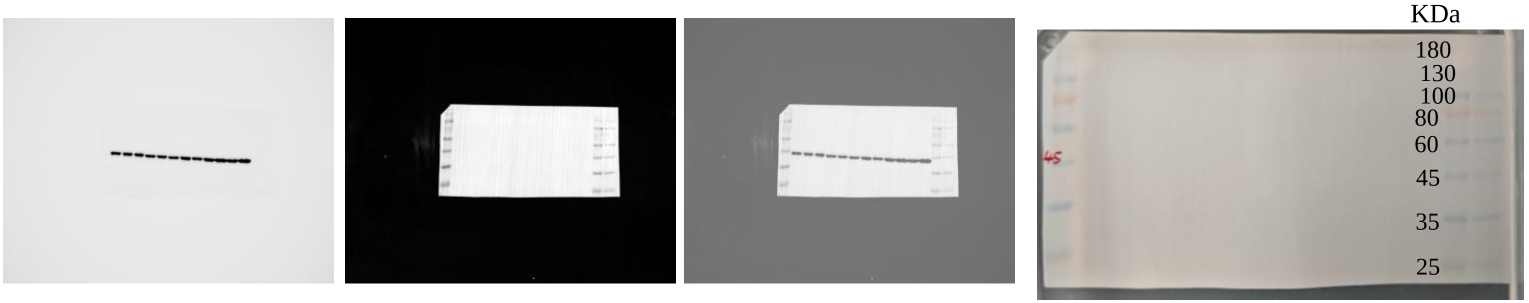

Western blot membrane of **β-actin (~42 kDa)** protein detected with anti-β-actin (BM0627; 1:8000; Boster, Wuhan, China) antibody. Gel-separated proteins were transferred to PVDF membranes (0.45 μm pore size; Millipore, Billerice, USA) by semidry electroblotting (1.3 A ,2.5V, 20 min). Membranes, incubated with a horseradish peroxidase-conjugated secondary antibody (BA1050; 1:5000–1:10000; Boster), were developed with Oriscience Supersensitive Kit (Oriscience Biotechnology). #Weight marker (molecular weight in kDa): Blue Plus IV Protein marker, 10 to 180 kDa; catalogue number: **R10519**. Blot images, prior to the densitometry readings, were converted to grayscale with ImageJ (ImageJ, National Institutes of Health, Maryland, USA).

2、PLIN5(OE-PLIN5） **figure2A**

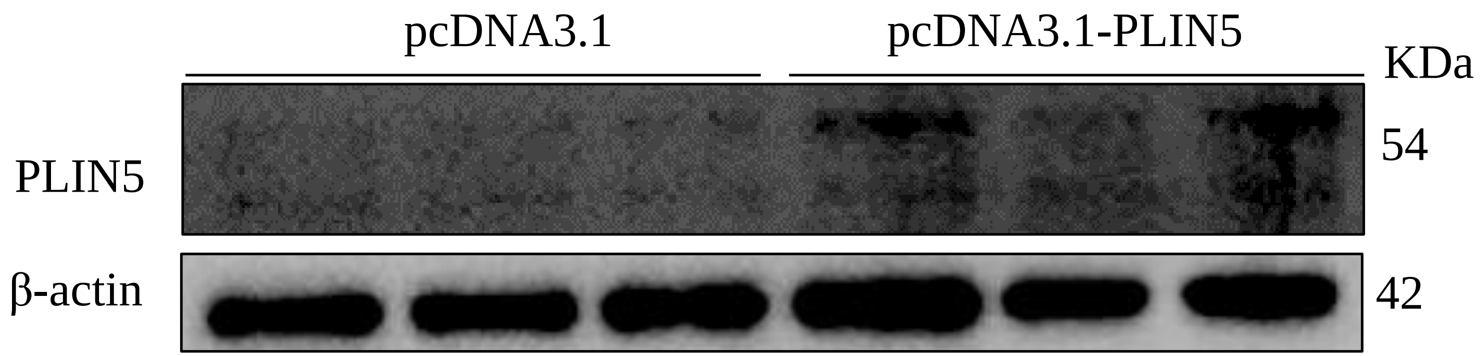

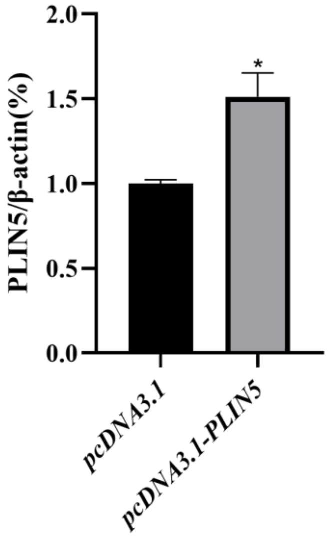

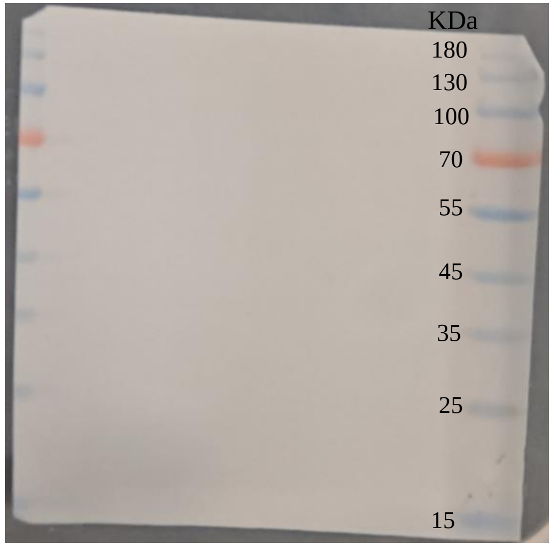

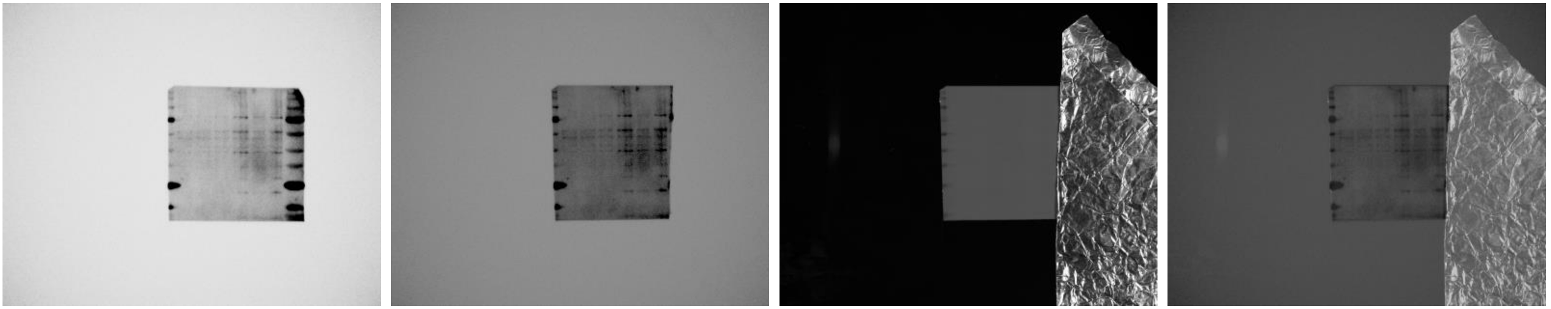

Western blot membrane of **PLIN5 (~55 kDa)** protein detected with anti-PLIN5 (A25598; 1:1000; ABclonal, Wuhan, China) antibody. Gel-separated proteins were transferred to PVDF membranes (0.45 μm pore size; Millipore, Billerice, USA) by semidry electroblotting (1.3 A,2.5V, 15 min). Membranes, incubated with a horseradish peroxidase-conjugated secondary antibody (BA1054; 1:5000–1:10000; Boater), were developed with Oriscience Supersensitive Kit (Oriscience Biotechnology). #Weight marker (molecular weight in kDa): Blue Plus IV Protein marker, 10 to 180 kDa; catalogue number: **R21223-V2**. Blot images, prior to the densitometry readings, were converted to grayscale with ImageJ (ImageJ, National Institutes of Health, Maryland, USA).

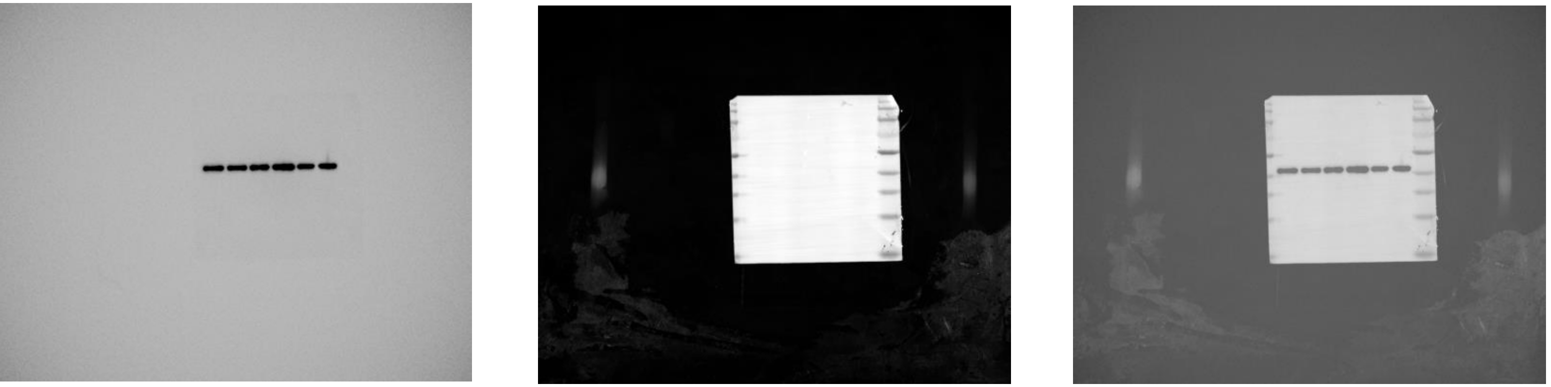

Western blot membrane of **β-actin (~42 kDa)** protein detected with anti-β-actin (BM0627; 1:8000; Boster, Wuhan, China) antibody. Gel-separated proteins were transferred to PVDF membranes (0.45 μm pore size; Millipore, Billerice, USA) by semidry electroblotting (1.3 A ,2.5V, 20 min). Membranes, incubated with a horseradish peroxidase-conjugated secondary antibody (BA1050; 1:5000–1:10000; Boster), were developed with Oriscience Supersensitive Kit (Oriscience Biotechnology). #Weight marker (molecular weight in kDa): Blue Plus IV Protein marker, 10 to 180 kDa; catalogue number: **R21223-V2**. Blot images, prior to the densitometry readings, were converted to grayscale with ImageJ (ImageJ, National Institutes of Health, Maryland, USA).

3、Caspase3(OE-PLIN5） **figure2G**

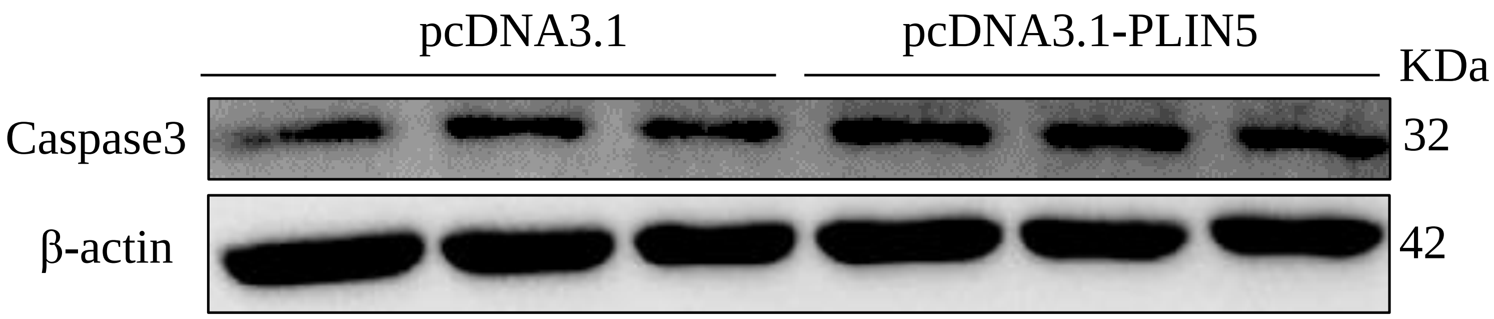

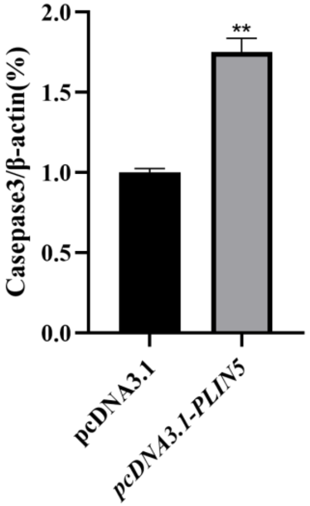

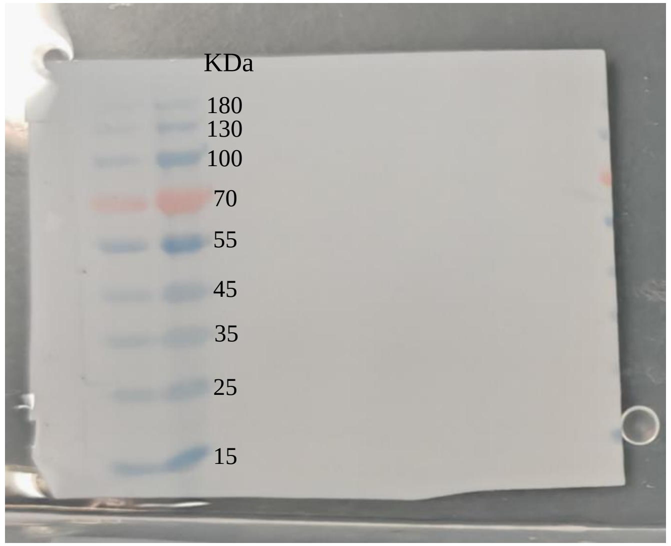

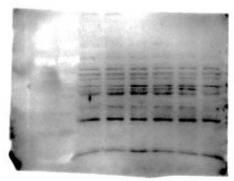

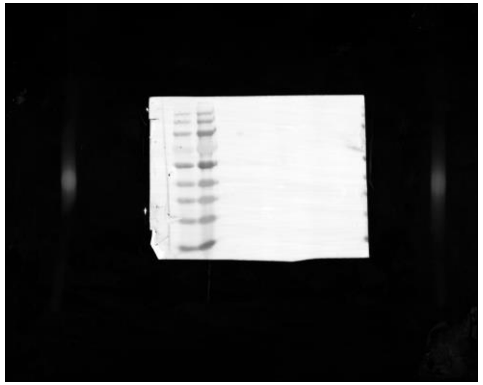

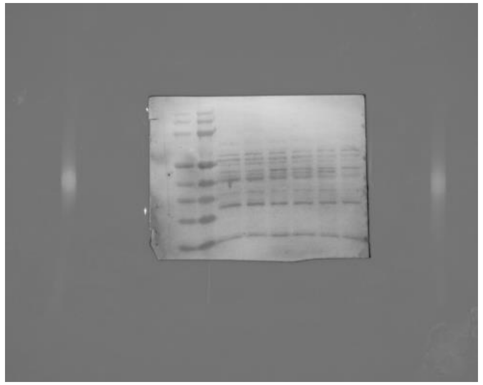

Western blot membrane of **Casepase3 (~32 kDa)** protein detected with anti-Caspase3 (WL02117; 1:500; Wanleibio, Shenyang, China) antibody. Gel-separated proteins were transferred to PVDF membranes (0.45 μm pore size; Millipore, Billerice, USA) by semidry electroblotting (1.3 A,2.5V, 15 min). Membranes, incubated with a horseradish peroxidase-conjugated secondary antibody (BA1054; 1:5000–1:10000; Boater), were developed with Oriscience Supersensitive Kit (Oriscience Biotechnology). #Weight marker (molecular weight in kDa): Blue Plus IV Protein marker, 10 to 180 kDa; catalogue number: **R21223-V2**. Blot images, prior to the densitometry readings, were converted to grayscale with ImageJ (ImageJ, National Institutes of Health, Maryland, USA).

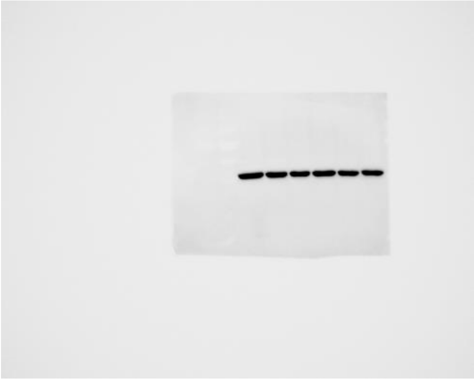

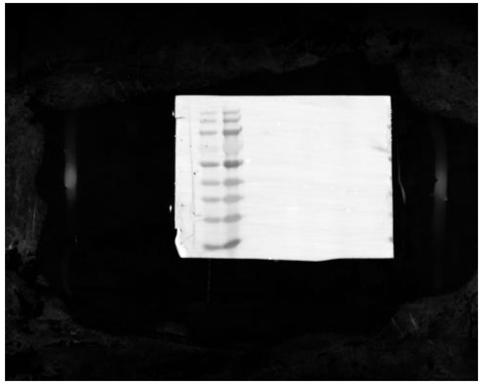

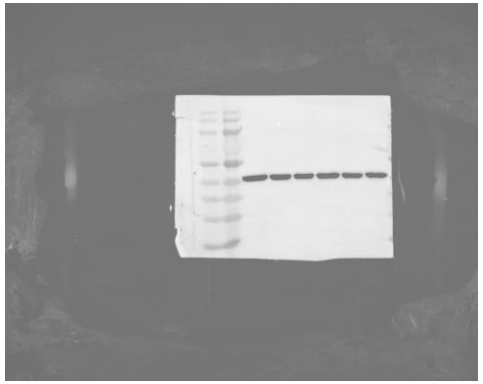

Western blot membrane of **β-actin (~42 kDa)** protein detected with anti-β-actin (BM0627; 1:8000; Boster, Wuhan, China) antibody. Gel-separated proteins were transferred to PVDF membranes (0.45 μm pore size; Millipore, Billerice, USA) by semidry electroblotting (1.3 A ,2.5V, 20 min). Membranes, incubated with a horseradish peroxidase-conjugated secondary antibody (BA1050; 1:5000–1:10000; Boster), were developed with Oriscience Supersensitive Kit (Oriscience Biotechnology). #Weight marker (molecular weight in kDa): Blue Plus IV Protein marker, 10 to 180 kDa; catalogue number: **R21223-V2**. Blot images, prior to the densitometry readings, were converted to grayscale with ImageJ (ImageJ, National Institutes of Health, Maryland, USA).

4、PLIN5(si-PLIN5） **figure3A**

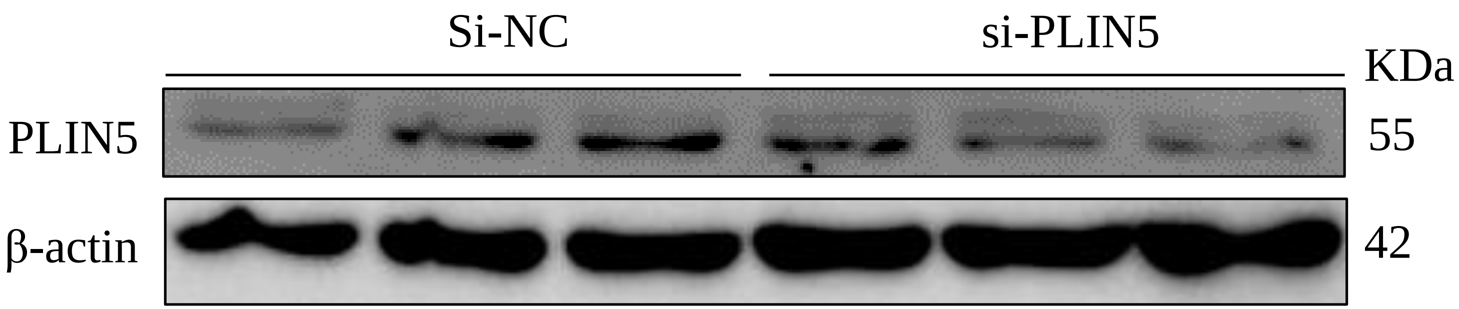

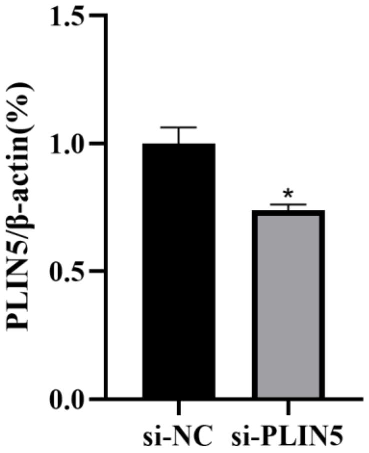

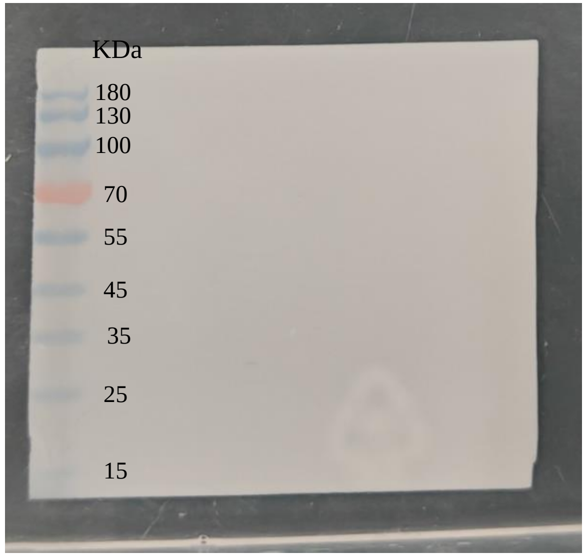

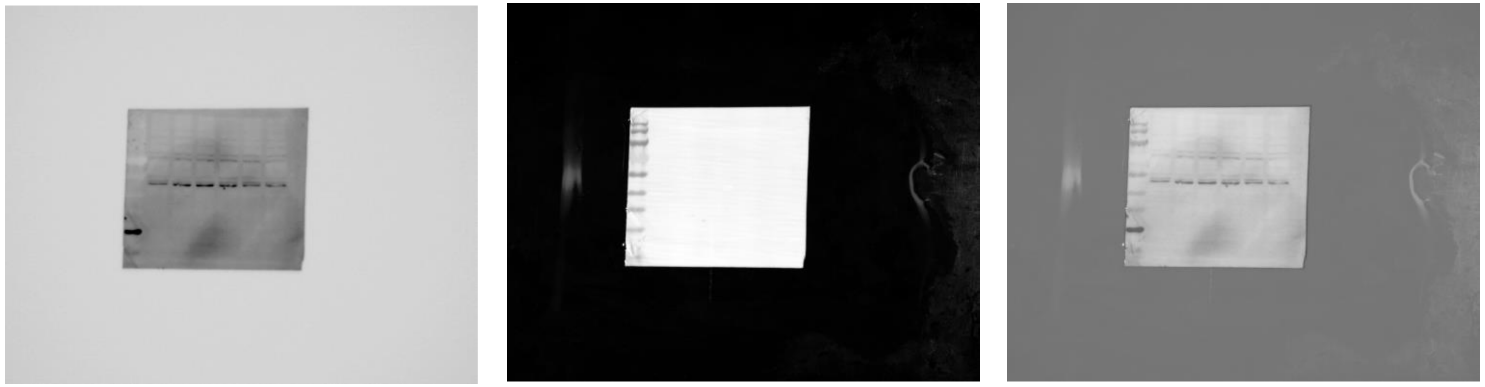

Western blot membrane of **PLIN5** (~55 kDa) protein detected with anti-PLIN5 (A25598; 1:1000; ABclonal, Wuhan, China) antibody. Gel-separated proteins were transferred to PVDF membranes (0.45 µm pore size; Millipore, Billerice, USA) by semidry electroblotting (1.3 A,2.5V, 15 min). Membranes, incubated with a horseradish peroxidase-conjugated secondary antibody (BA1054; 1:5000–1:10000; Boater), were developed with Oriscience Supersensitive Kit (Oriscience Biotechnology). #Weight marker (molecular weight in kDa): Blue Plus IV Protein marker, 10 to 180 kDa; catalogue number: **R21223-V2**. Blot images, prior to the densitometry readings, were converted to grayscale with ImageJ (ImageJ, National Institutes of Health, Maryland, USA).

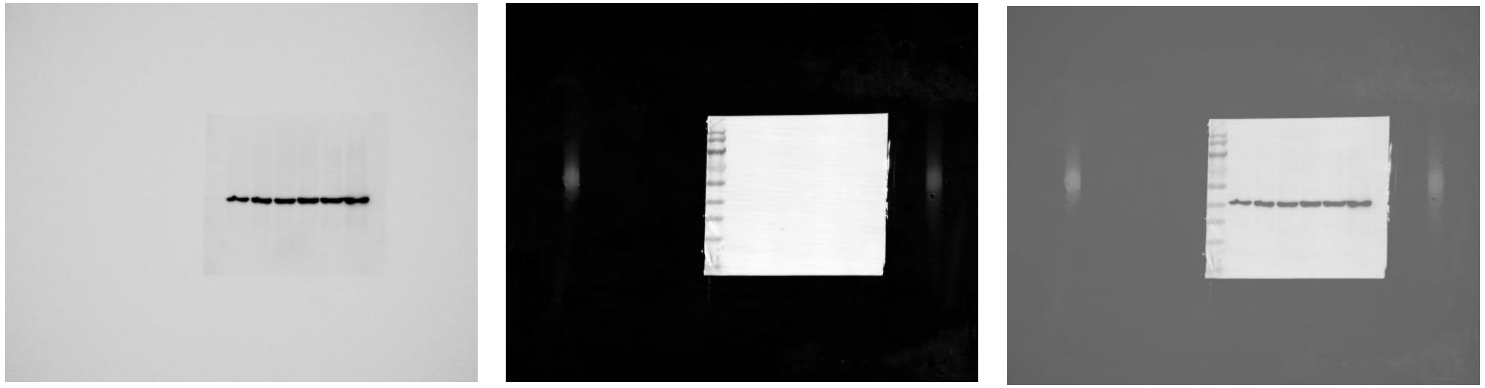

Western blot membrane of **β-actin** (~42 kDa) protein detected with anti-β-actin (BM0627; 1:8000; Boster, Wuhan, China) antibody. Gel-separated proteins were transferred to PVDF membranes (0.45 µm pore size; Millipore, Billerice, USA) by semidry electroblotting (1.3 A ,2.5V, 20 min). Membranes, incubated with a horseradish peroxidase-conjugated secondary antibody (BA1050; 1:5000–1:10000; Boster), were developed with Oriscience Supersensitive Kit (Oriscience Biotechnology). #Weight marker (molecular weight in kDa): Blue Plus IV Protein marker, 10 to 180 kDa; catalogue number: **R21223-V2**. Blot images, prior to the densitometry readings, were converted to grayscale with ImageJ (ImageJ, National Institutes of Health, Maryland, USA).

5、Caspase3(si-PLIN5） **figure3G**

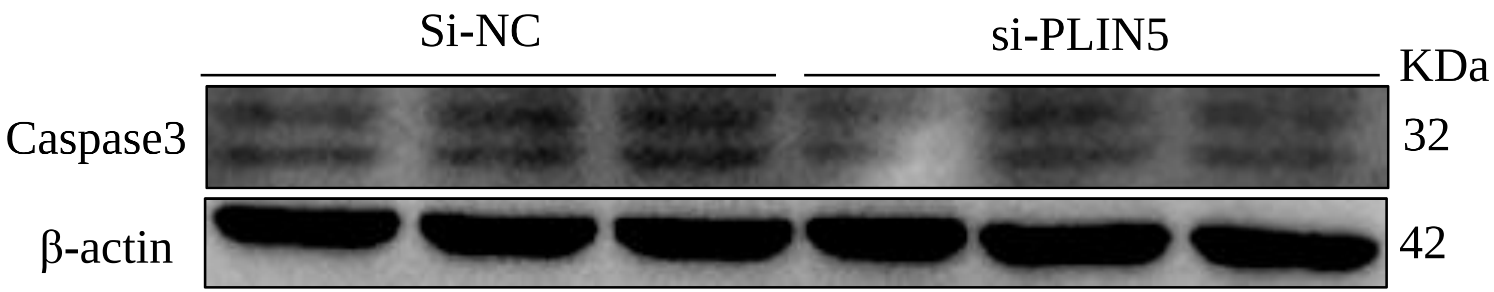

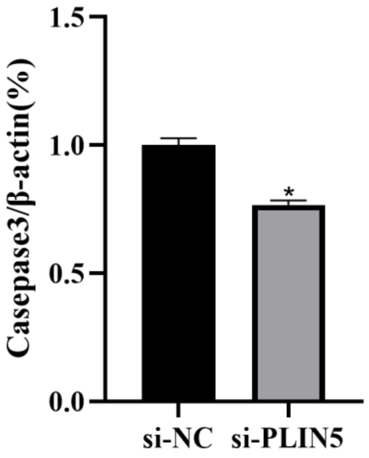

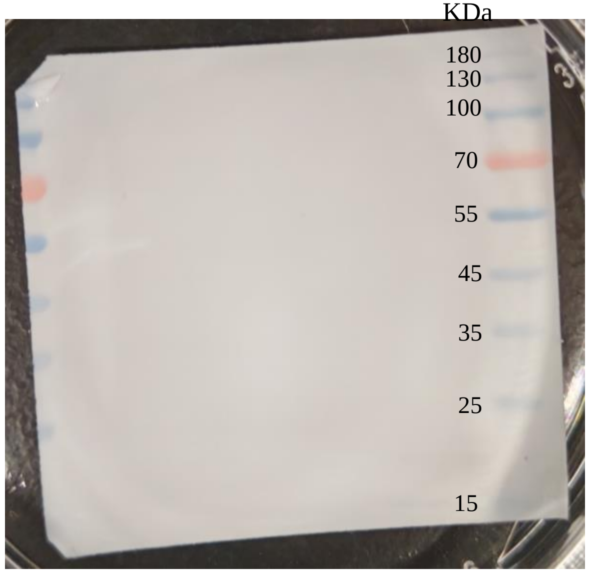

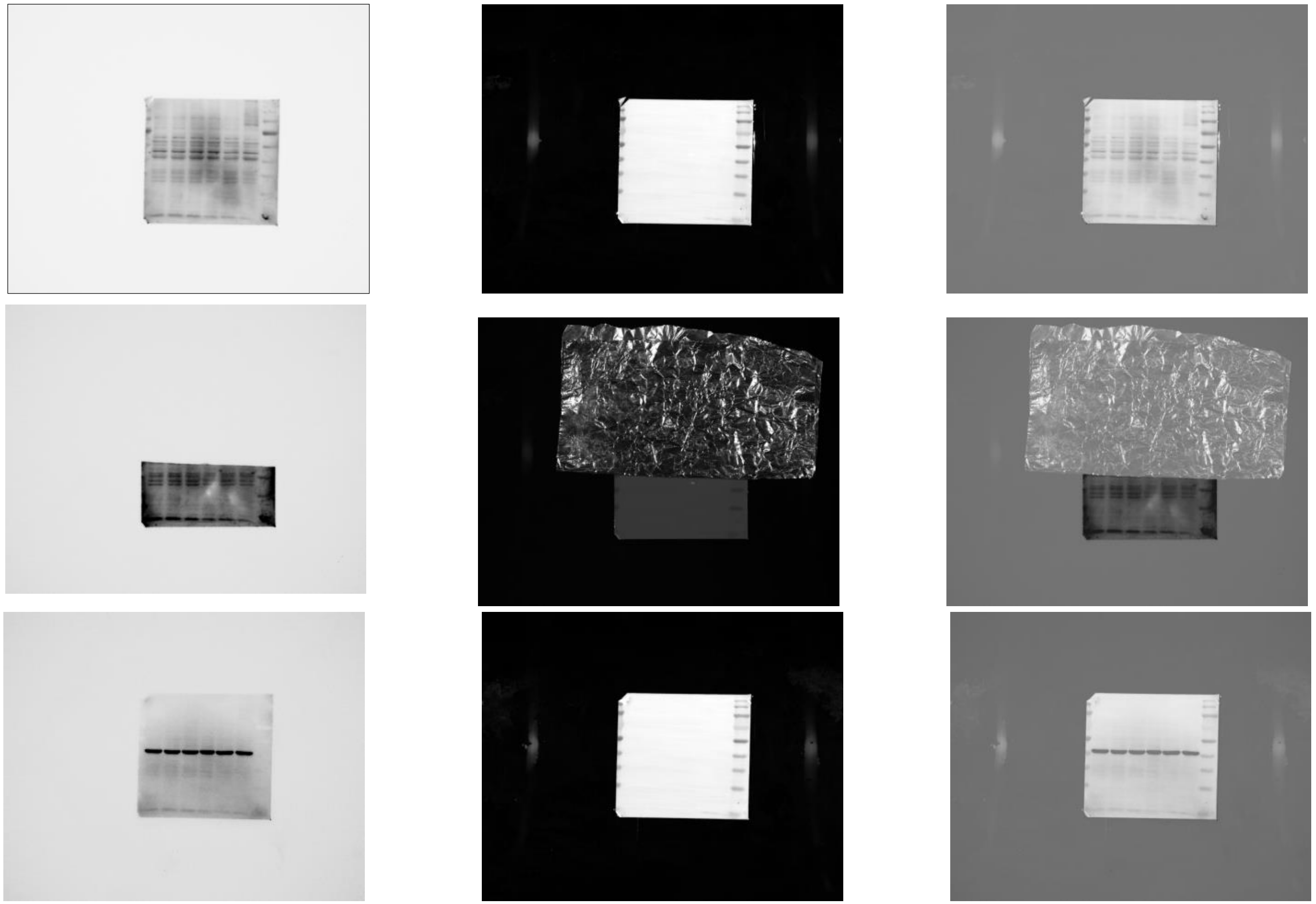

Western blot membrane of **Casepase3 (~32 kDa)** protein detected with anti-Caspase3 (WL02117; 1:500; Wanleibio, Shenyang, China) antibody. Gel-separated proteins were transferred to PVDF membranes (0.45 µm pore size; Millipore, Billerice, USA) by semidry electroblotting (1.3 A,2.5V, 15 min). Membranes, incubated with a horseradish peroxidase-conjugated secondary antibody (BA1054; 1:5000–1:10000; Boater), were developed with Oriscience Supersensitive Kit (Oriscience Biotechnology). #Weight marker (molecular weight in kDa): Blue Plus IV Protein marker, 10 to 180 kDa; catalogue number**: R21223-V2**. Blot images, prior to the densitometry readings, were converted to grayscale with ImageJ (ImageJ, National Institutes of Health, Maryland, USA).

Western blot membrane of **β-actin (~42 kDa**) protein detected with anti-β-actin (BM0627; 1:8000; Boster, Wuhan, China) antibody. Gel-separated proteins were transferred to PVDF membranes (0.45 µm pore size; Millipore, Billerice, USA) by semidry electroblotting (1.3 A ,2.5V, 20 min). Membranes, incubated with a horseradish peroxidase-conjugated secondary antibody (BA1050; 1:5000–1:10000; Boster), were developed with Oriscience Supersensitive Kit (Oriscience Biotechnology). #Weight marker (molecular weight in kDa): Blue Plus IV Protein marker, 10 to 180 kDa; catalogue number: **R21223-V2**. Blot images, prior to the densitometry readings, were converted to grayscale with ImageJ (ImageJ, National Institutes of Health, Maryland, USA).

6-1、P-AKT(OE-PLIN5）**figure5A**

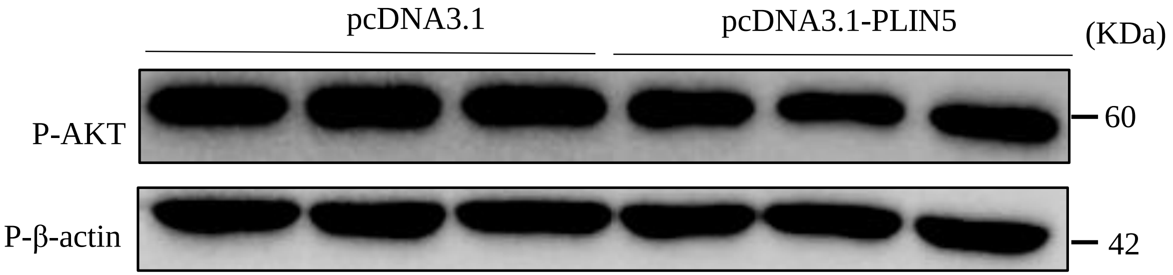

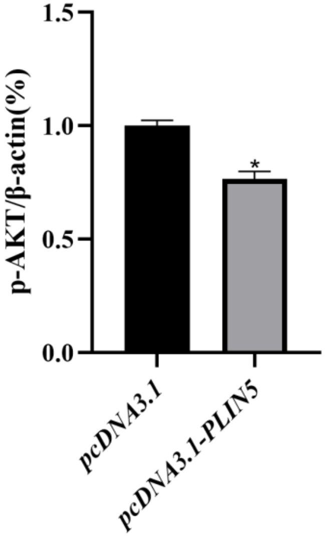

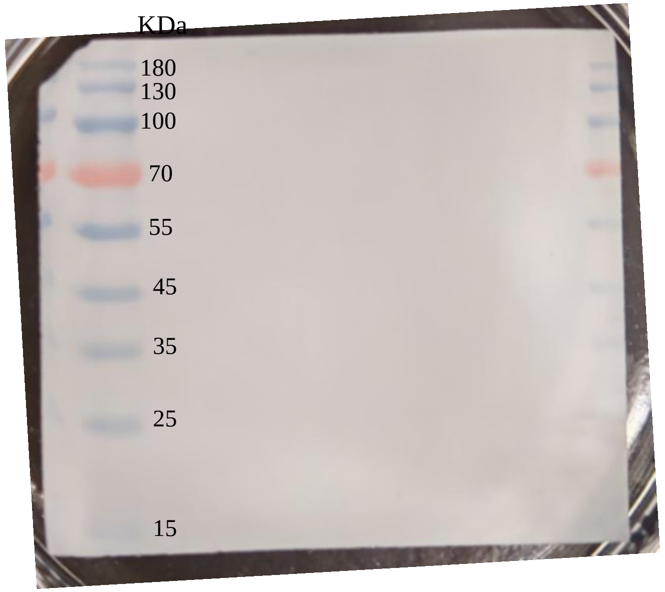

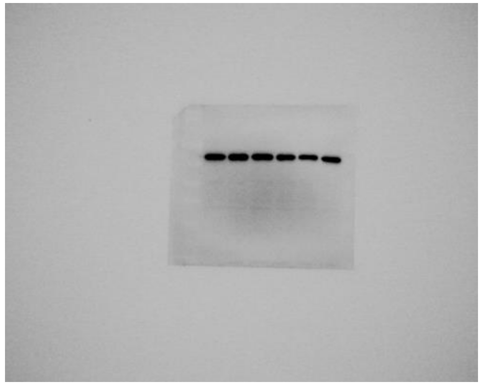
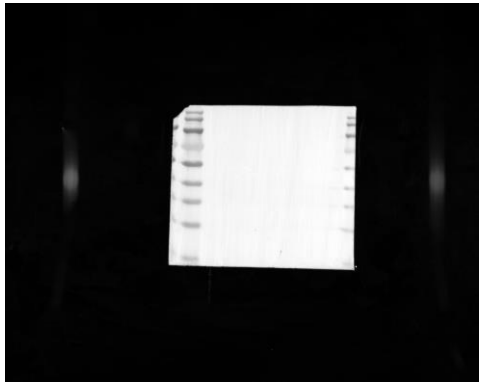
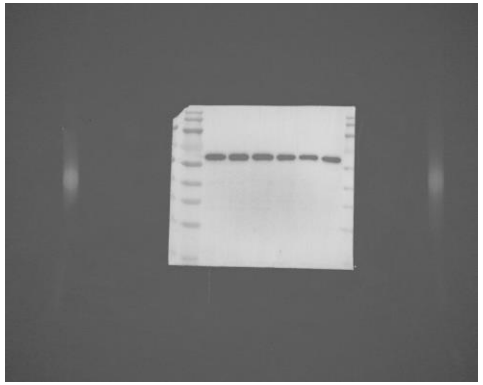

Western blot membrane of **p-AKT (~60 kDa)** protein detected with anti-p-AKT1-Ser473 (4060; 1:2000; Cell Signaling Technology, Massachusetts, USA) antibody. Gel-separated proteins were transferred to PVDF membranes (0.45 μm pore size; Millipore, Billerice, USA) by semidry electroblotting (1.3 A,2.5V, 15 min). Membranes, incubated with a horseradish peroxidase-conjugated secondary antibody (BA1054; 1:5000–1:10000; Boater), were developed with Oriscience Supersensitive Kit (Oriscience Biotechnology). #Weight marker (molecular weight in kDa): Blue Plus IV Protein marker, 10 to 180 kDa; catalogue number: **R10519**. Blot images, prior to the densitometry readings, were converted to grayscale with ImageJ (ImageJ, National Institutes of Health, Maryland, USA).

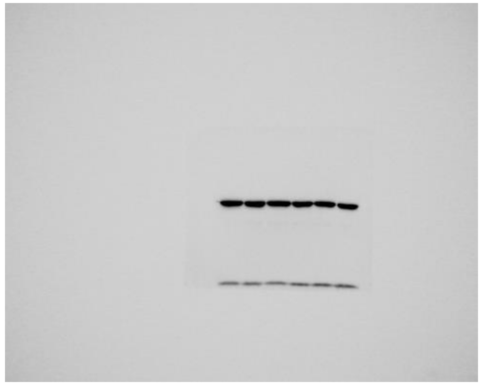
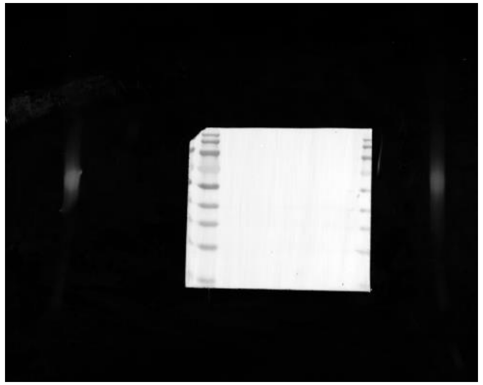
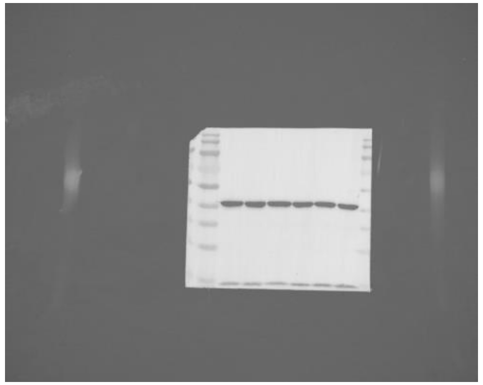

Western blot membrane of **β-actin** (~42 kDa) protein detected with anti-β-actin (BM0627; 1:8000; Boster, Wuhan, China) antibody. Gel-separated proteins were transferred to PVDF membranes (0.45 μm pore size; Millipore, Billerice, USA) by semidry electroblotting (1.3 A ,2.5V, 20 min). Membranes, incubated with a horseradish peroxidase-conjugated secondary antibody (BA1050; 1:5000–1:10000; Boster), were developed with Oriscience Supersensitive Kit (Oriscience Biotechnology). #Weight marker (molecular weight in kDa): Blue Plus IV Protein marker, 10 to 180 kDa; catalogue number: **R10519**. Blot images, prior to the densitometry readings, were converted to grayscale with ImageJ (ImageJ, National Institutes of Health, Maryland, USA).

6-2、AKT(OE-PLIN5） **figure5A**

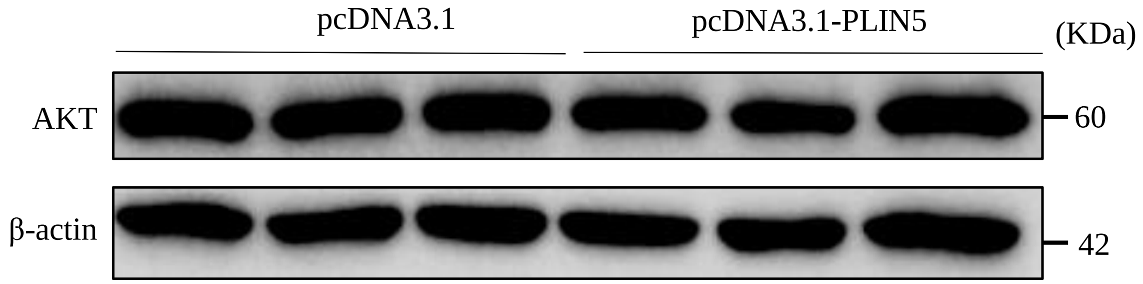

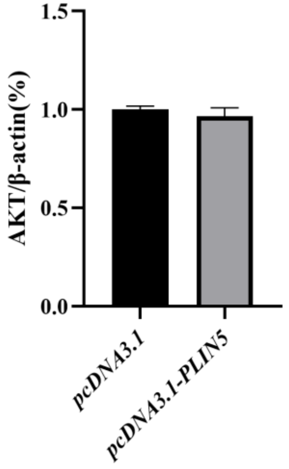

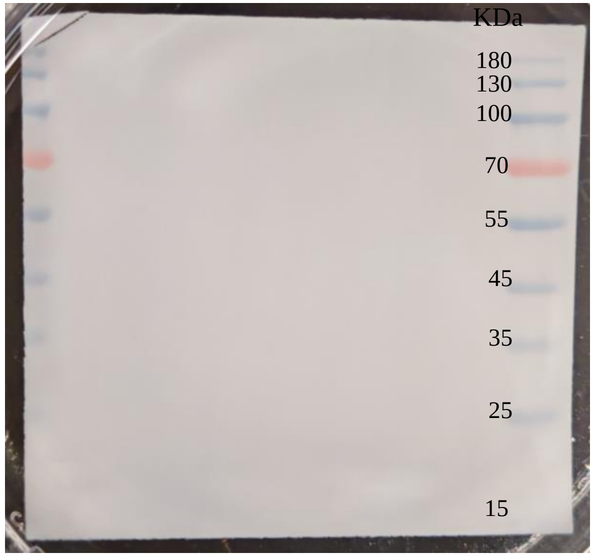

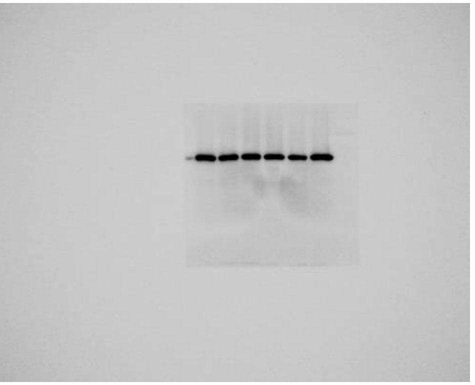

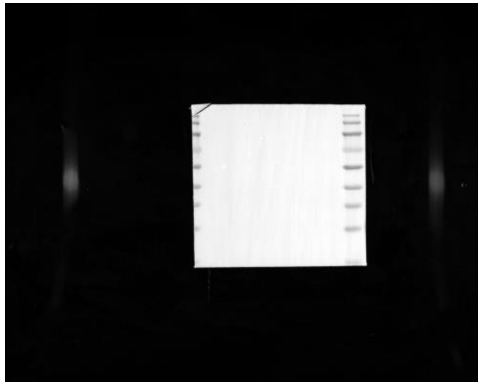

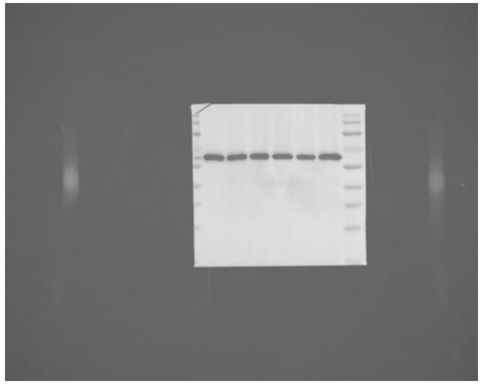

Western blot membrane of **AKT1 (~60 kDa)** protein detected with anti-AKT1 (ab32505; 1:1000; ab32505, Cambridge, US) antibody. Gel-separated proteins were transferred to PVDF membranes (0.45 μm pore size; Millipore, Billerice, USA) by semidry electroblotting (1.3 A,2.5V, 15 min). Membranes, incubated with a horseradish peroxidase-conjugated secondary antibody (BA1054; 1:5000–1:10000; Boater), were developed with Oriscience Supersensitive Kit (Oriscience Biotechnology). #Weight marker (molecular weight in kDa): Blue Plus IV Protein marker, 10 to 180 kDa; catalogue number: **R21223-V2**. Blot images, prior to the densitometry readings, were converted to grayscale with ImageJ (ImageJ, National Institutes of Health, Maryland, USA).

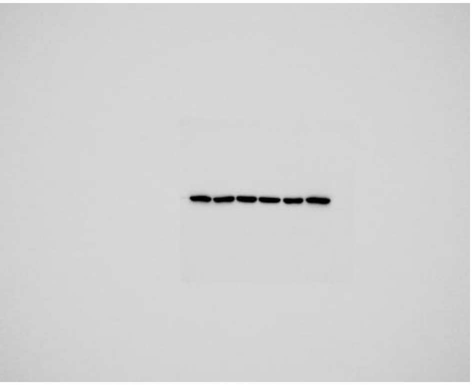

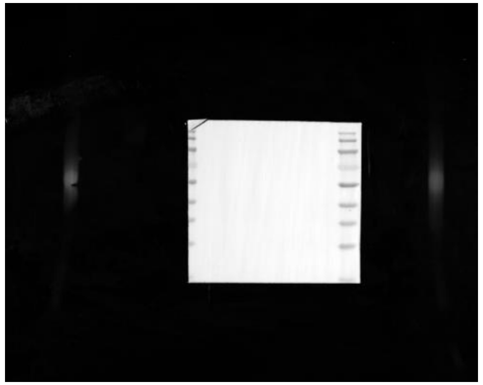

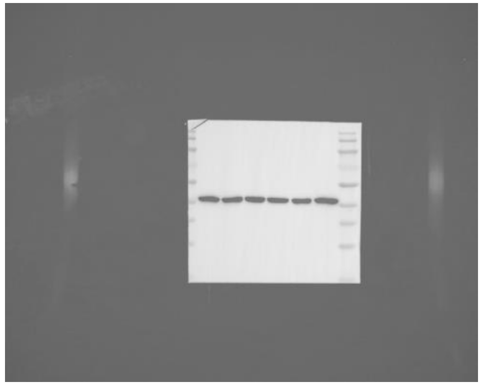

Western blot membrane of **β-actin (~42 kDa)** protein detected with anti-β-actin (BM0627; 1:8000; Boster, Wuhan, China) antibody. Gel-separated proteins were transferred to PVDF membranes (0.45 μm pore size; Millipore, Billerice, USA) by semidry electroblotting (1.3 A ,2.5V, 20 min). Membranes, incubated with a horseradish peroxidase-conjugated secondary antibody (BA1050; 1:5000–1:10000; Boster), were developed with Oriscience Supersensitive Kit (Oriscience Biotechnology). #Weight marker (molecular weight in kDa): Blue Plus IV Protein marker, 10 to 180 kDa; catalogue number: **R21223-V2**. Blot images, prior to the densitometry readings, were converted to grayscale with ImageJ (ImageJ, National Institutes of Health, Maryland, USA).

6-3、p-AKT/AKT (OE-PLIN5） **figure5A**

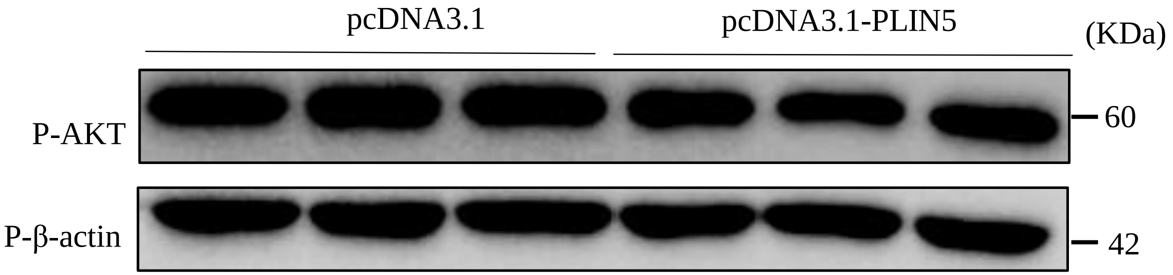

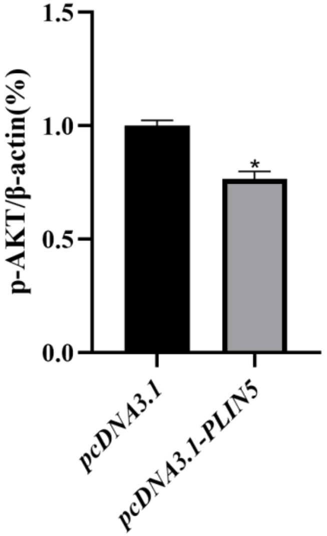

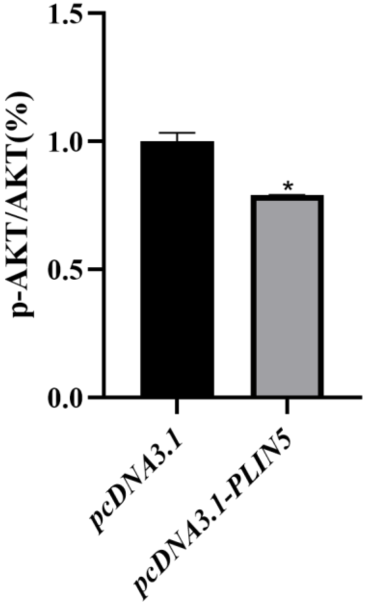

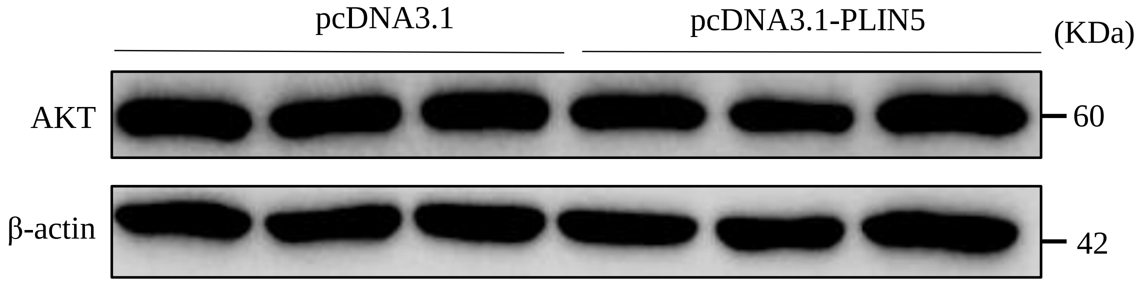

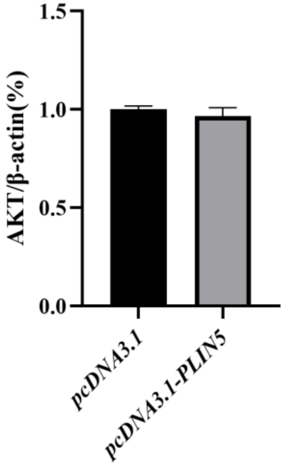

7-1、P-AKT(si-PLIN5） **figure5B**

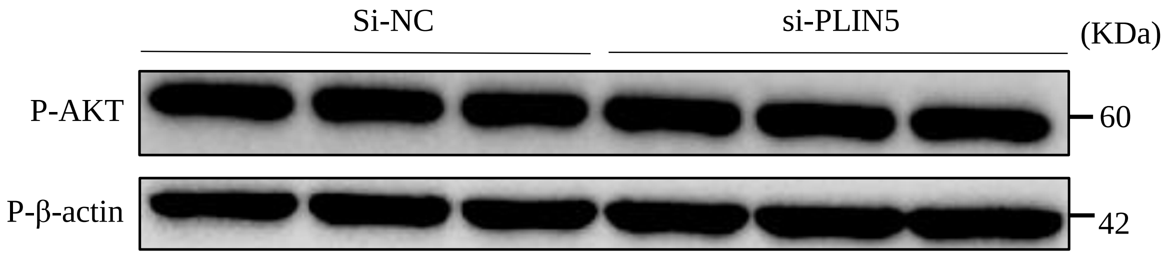

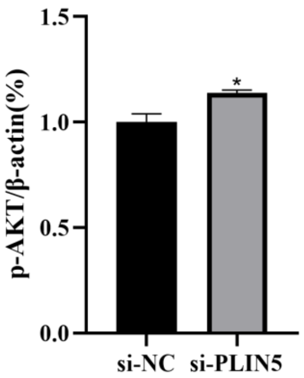

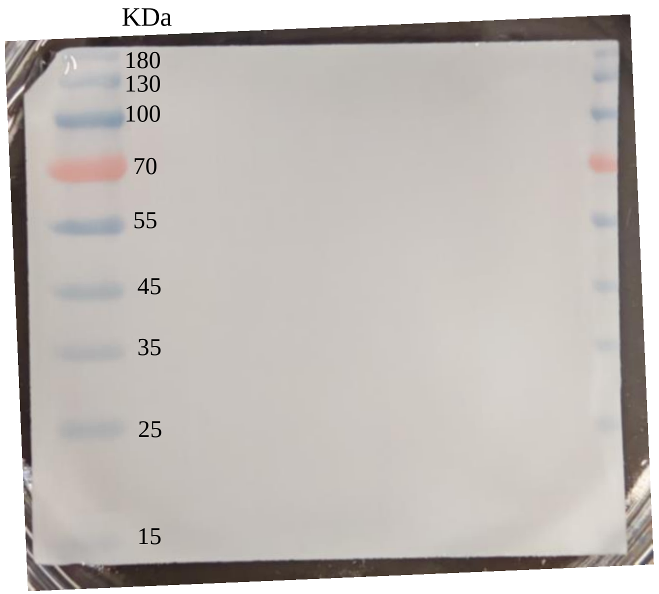

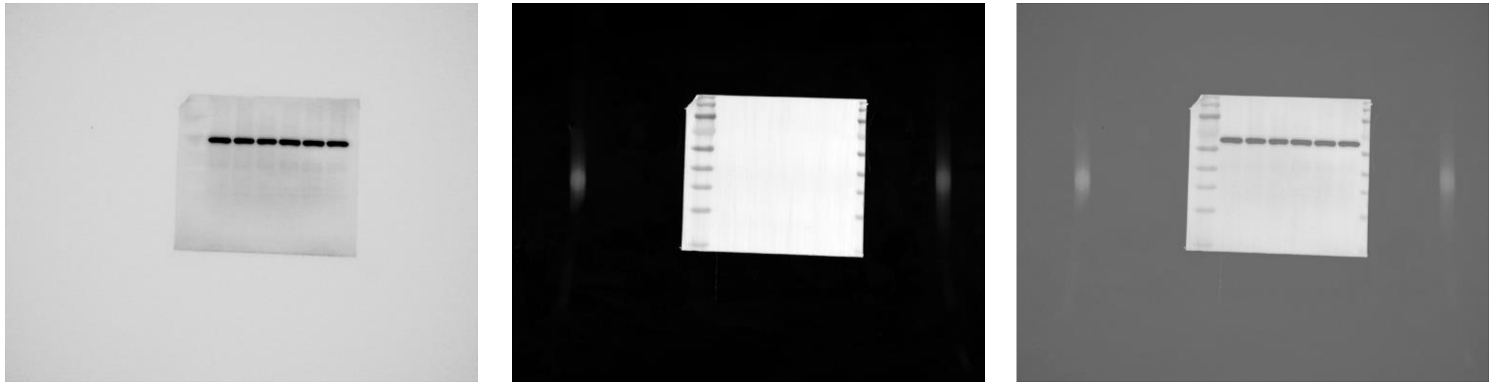

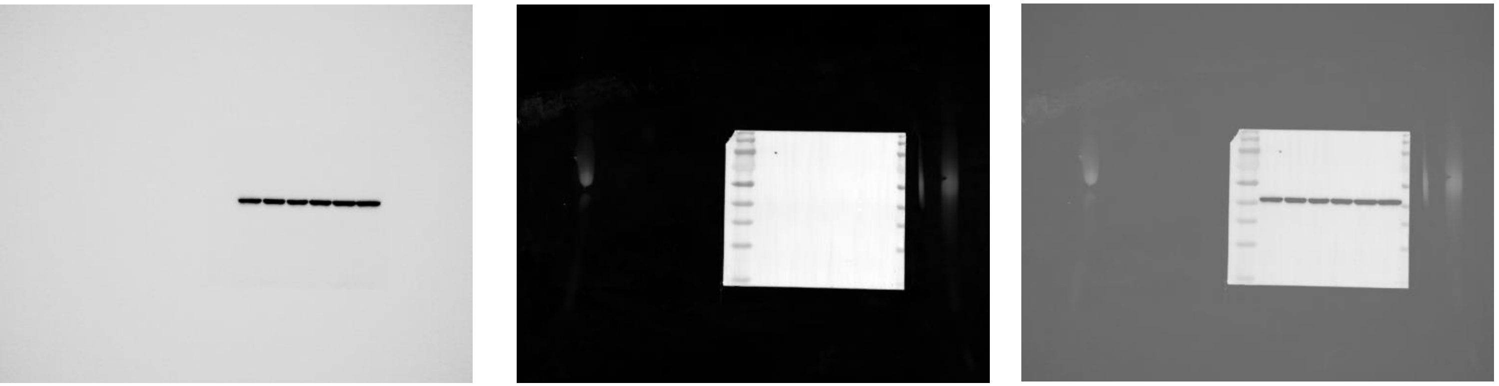

Western blot membrane of **p-AKT (~60 kDa)** protein detected with anti-p-AKT1-Ser473 (4060; 1:2000; Cell Signaling Technology, Massachusetts, USA) antibody. Gel-separated proteins were transferred to PVDF membranes (0.45 μm pore size; Millipore, Billerice, USA) by semidry electroblotting (1.3 A,2.5V, 15 min). Membranes, incubated with a horseradish peroxidase-conjugated secondary antibody (BA1054; 1:5000–1:10000; Boater), were developed with Oriscience Supersensitive Kit (Oriscience Biotechnology). #Weight marker (molecular weight in kDa): Blue Plus IV Protein marker, 10 to 180 kDa; catalogue number: **R21223-V2**. Blot images, prior to the densitometry readings, were converted to grayscale with ImageJ (ImageJ, National Institutes of Health, Maryland, USA).

Western blot membrane of **β-actin** (~42 kDa) protein detected with anti-β-actin (BM0627; 1:8000; Boster, Wuhan, China) antibody. Gel-separated proteins were transferred to PVDF membranes (0.45 μm pore size; Millipore, Billerice, USA) by semidry electroblotting (1.3 A ,2.5V, 20 min). Membranes, incubated with a horseradish peroxidase-conjugated secondary antibody (BA1050; 1:5000–1:10000; Boster), were developed with Oriscience Supersensitive Kit (Oriscience Biotechnology). #Weight marker (molecular weight in kDa): Blue Plus IV Protein marker, 10 to 180 kDa; catalogue number: **R21223-V2**. Blot images, prior to the densitometry readings, were converted to grayscale with ImageJ (ImageJ, National Institutes of Health, Maryland, USA).

7-2、AKT(si-PLIN5） **figure5B**

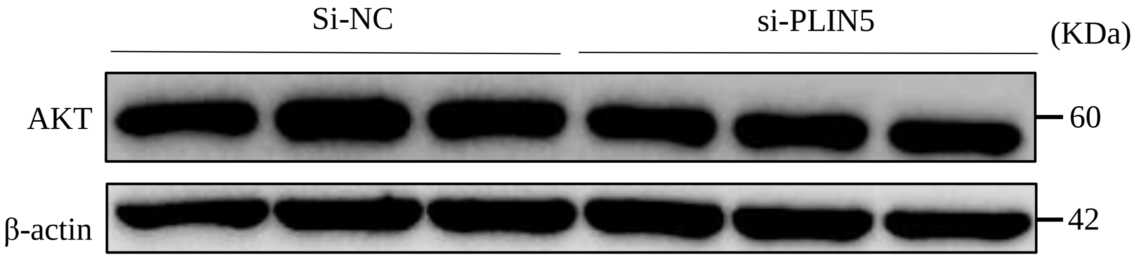

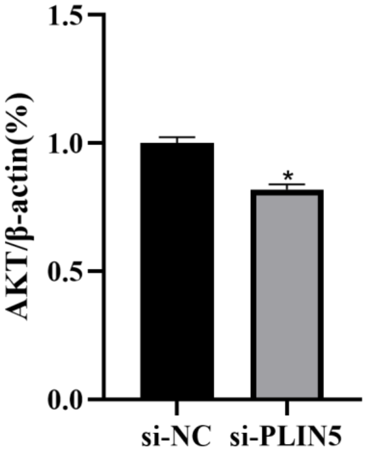

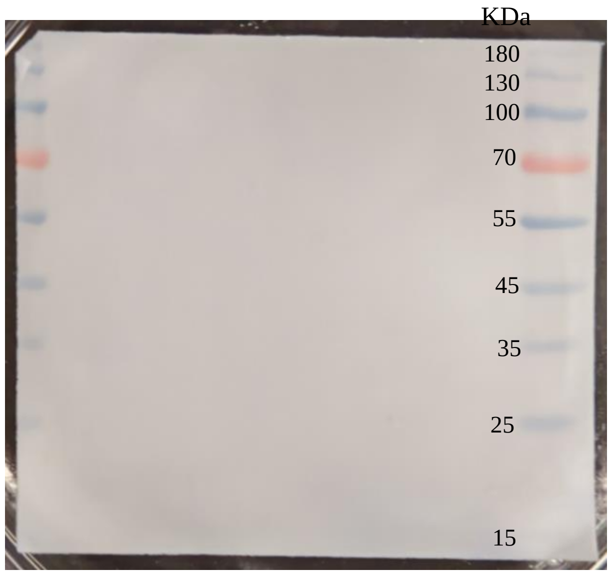

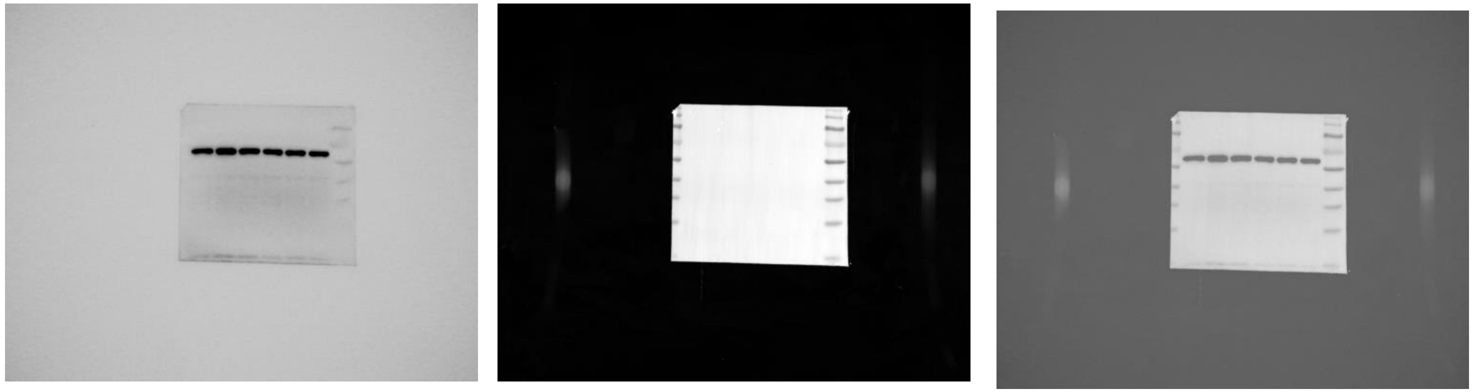

Western blot membrane of **AKT1 (~60 kDa)** protein detected with anti-AKT1 (ab32505; 1:1000; ab32505, Cambridge, US) antibody. Gel-separated proteins were transferred to PVDF membranes (0.45 μm pore size; Millipore, Billerice, USA) by semidry electroblotting (1.3 A,2.5V, 15 min). Membranes, incubated with a horseradish peroxidase-conjugated secondary antibody (BA1054; 1:5000–1:10000; Boater), were developed with Oriscience Supersensitive Kit (Oriscience Biotechnology). #Weight marker (molecular weight in kDa): Blue Plus IV Protein marker, 10 to 180 kDa; catalogue number: **R21223-V2**. Blot images, prior to the densitometry readings, were converted to grayscale with ImageJ (ImageJ, National Institutes of Health, Maryland, USA).

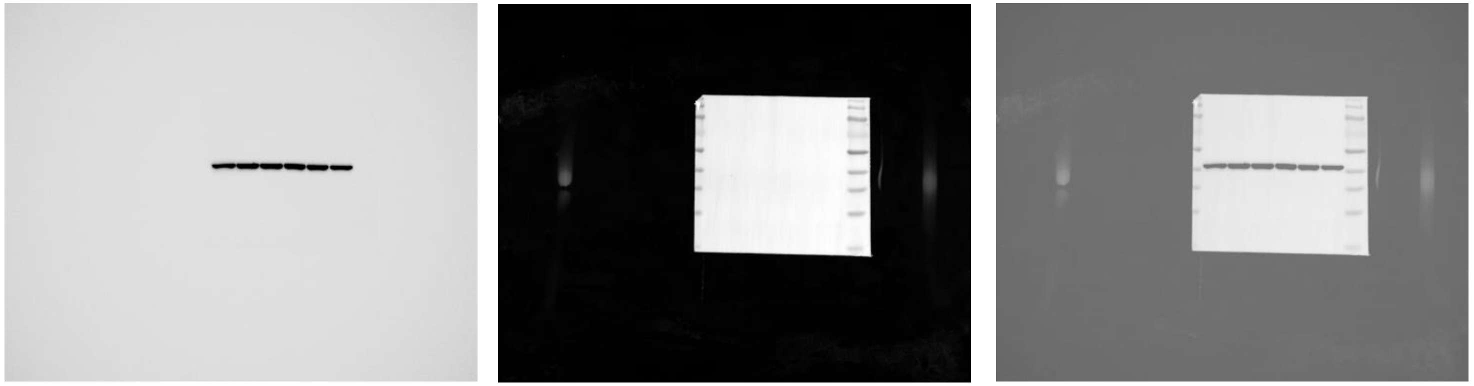

Western blot membrane of **β-actin** (~42 kDa) protein detected with anti-β-actin (BM0627; 1:8000; Boster, Wuhan, China) antibody. Gel-separated proteins were transferred to PVDF membranes (0.45 μm pore size; Millipore, Billerice, USA) by semidry electroblotting (1.3 A ,2.5V, 20 min). Membranes, incubated with a horseradish peroxidase-conjugated secondary antibody (BA1050; 1:5000–1:10000; Boster), were developed with Oriscience Supersensitive Kit (Oriscience Biotechnology). #Weight marker (molecular weight in kDa): Blue Plus IV Protein marker, 10 to 180 kDa; catalogue number: **R21223-V2**. Blot images, prior to the densitometry readings, were converted to grayscale with ImageJ (ImageJ, National Institutes of Health, Maryland, USA).

7-3、p-AKT/AKT (si-PLIN5）**figure5B**

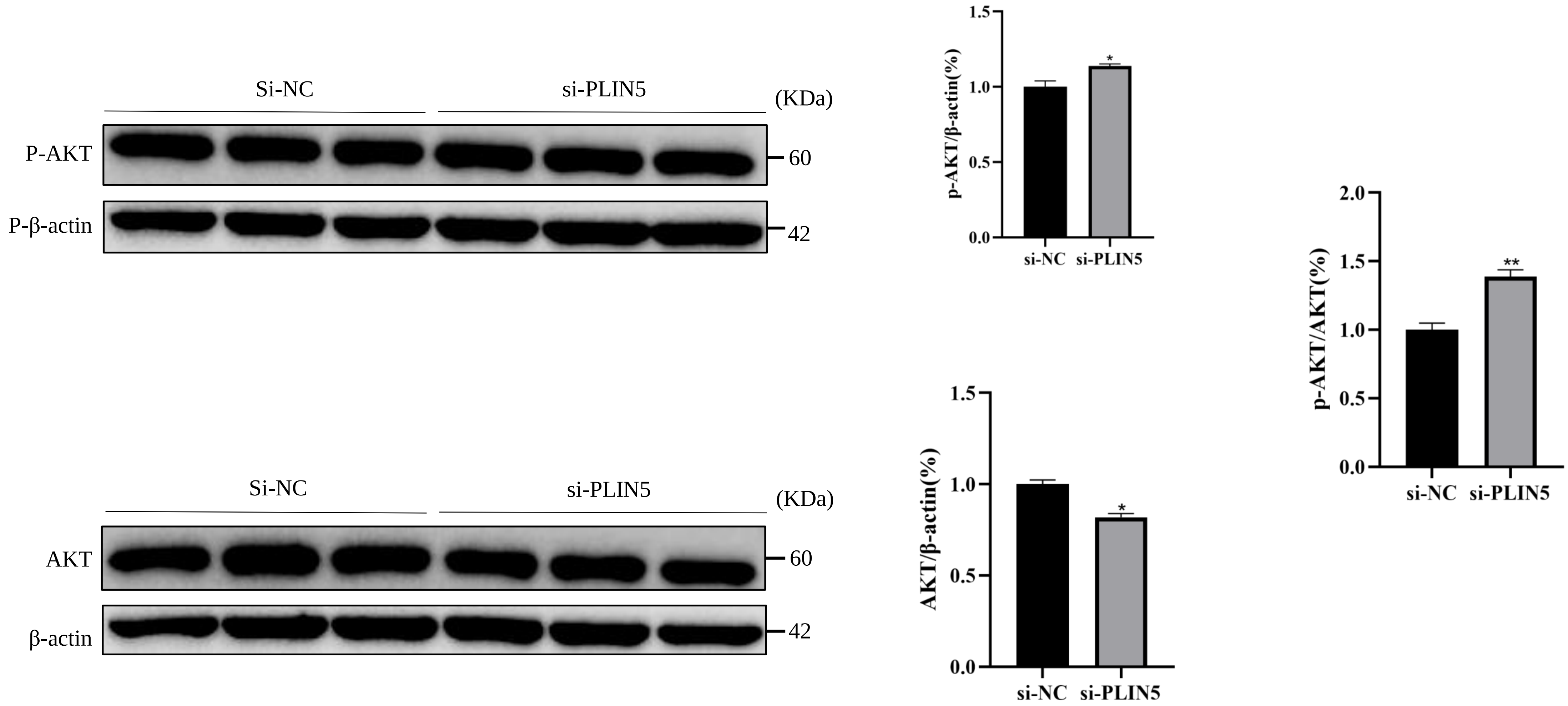

8-1、P-AKT(+LY294002）**figure5C**

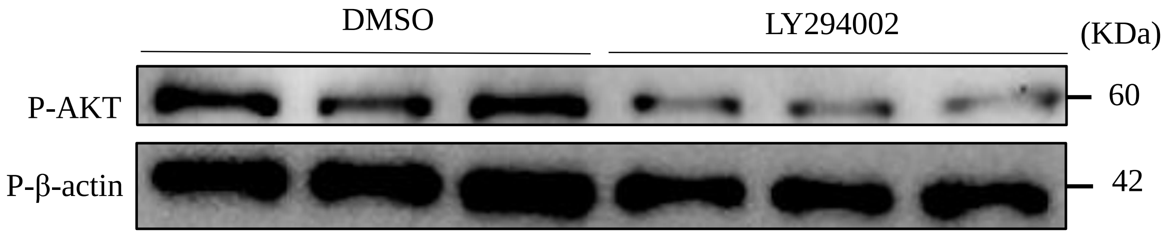

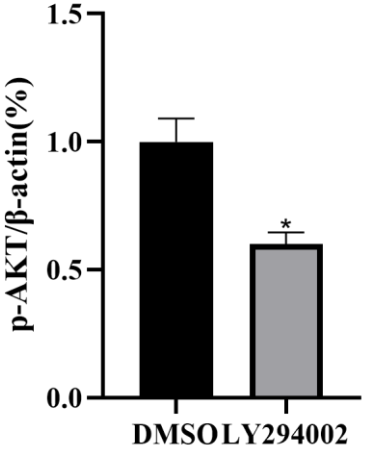

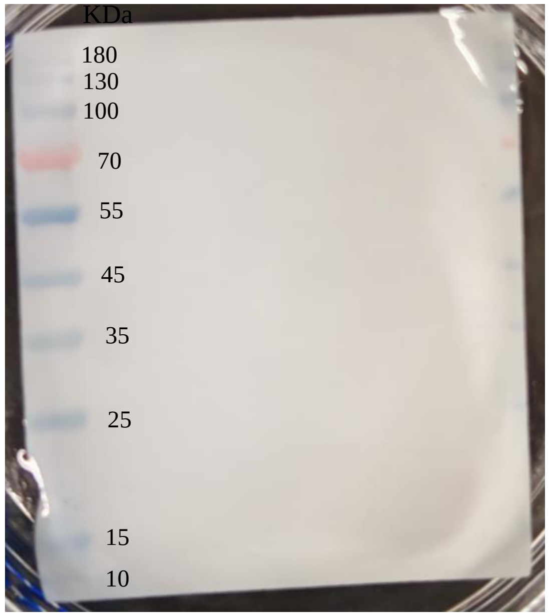

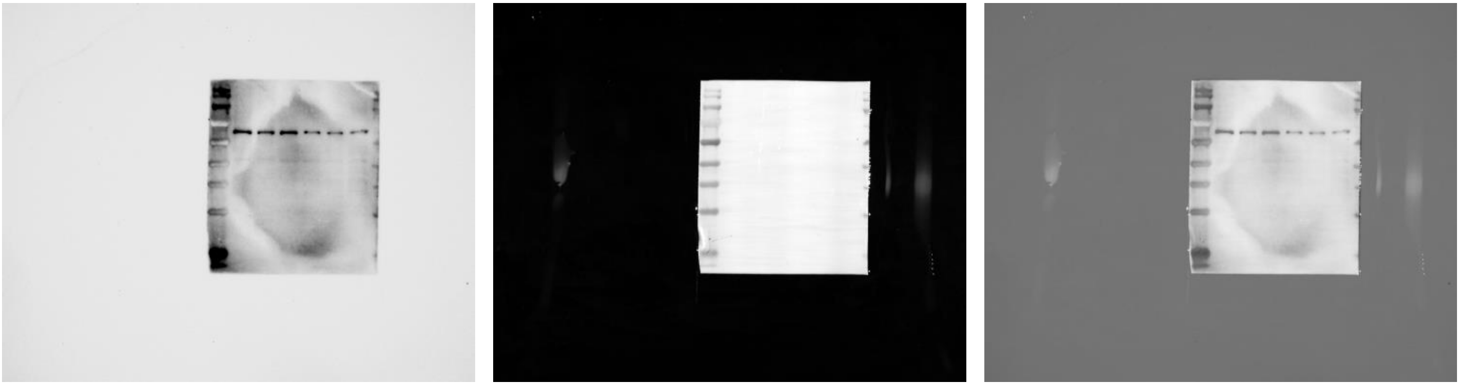

Western blot membrane of **p-AKT (~60 kDa)** protein detected with anti-p-AKT1-Ser473 (4060; 1:2000; Cell Signaling Technology, Massachusetts, USA) antibody. Gel-separated proteins were transferred to PVDF membranes (0.45 μm pore size; Millipore, Billerice, USA) by semidry electroblotting (1.3 A,2.5V, 15 min). Membranes, incubated with a horseradish peroxidase-conjugated secondary antibody (BA1054; 1:5000–1:10000; Boater), were developed with Oriscience Supersensitive Kit (Oriscience Biotechnology). #Weight marker (molecular weight in kDa): Blue Plus IV Protein marker, 10 to 180 kDa; catalogue number: **R21223-V2**. Blot images, prior to the densitometry readings, were converted to grayscale with ImageJ (ImageJ, National Institutes of Health, Maryland, USA).

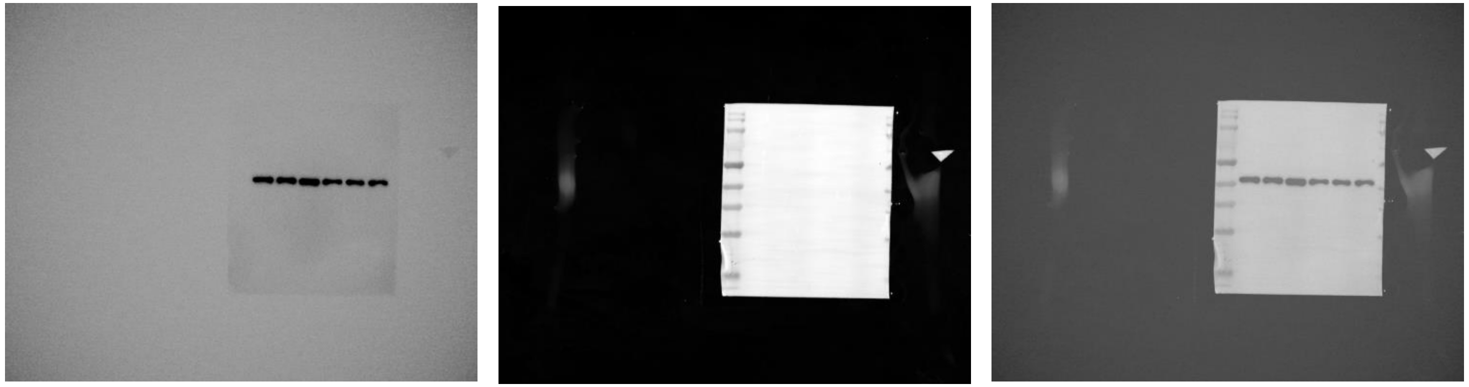

Western blot membrane of **β-actin** (~42 kDa) protein detected with anti-β-actin (BM0627; 1:8000; Boster, Wuhan, China) antibody. Gel-separated proteins were transferred to PVDF membranes (0.45 μm pore size; Millipore, Billerice, USA) by semidry electroblotting (1.3 A ,2.5V, 20 min). Membranes, incubated with a horseradish peroxidase-conjugated secondary antibody (BA1050; 1:5000–1:10000; Boster), were developed with Oriscience Supersensitive Kit (Oriscience Biotechnology). #Weight marker (molecular weight in kDa): Blue Plus IV Protein marker, 10 to 180 kDa; catalogue number: **R21223-V2**. Blot images, prior to the densitometry readings, were converted to grayscale with ImageJ (ImageJ, National Institutes of Health, Maryland, USA).

8-2、AKT (+LY294002） figure5C

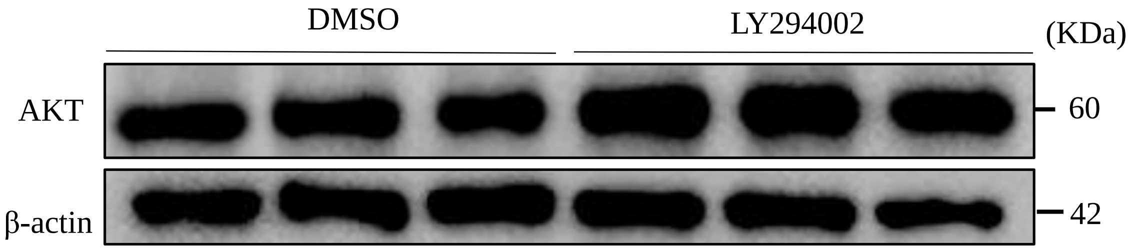

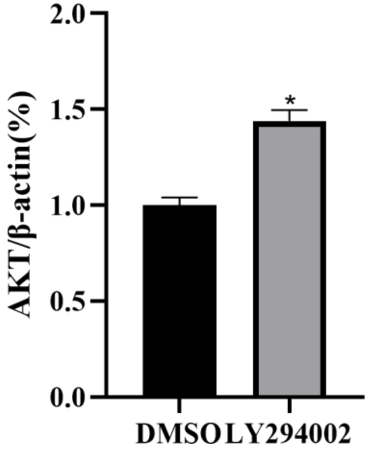

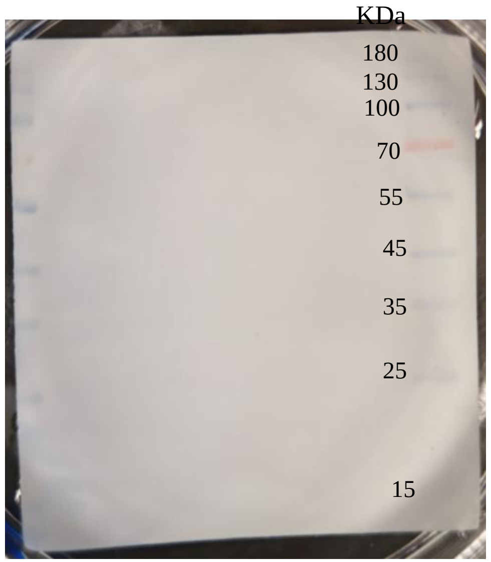

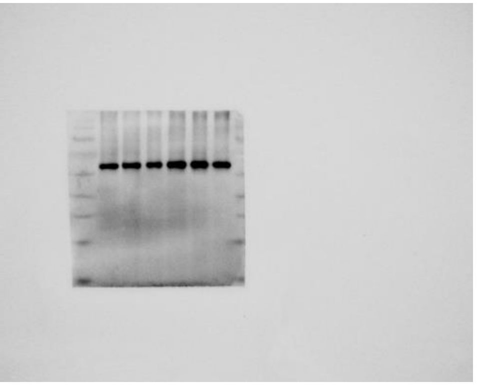
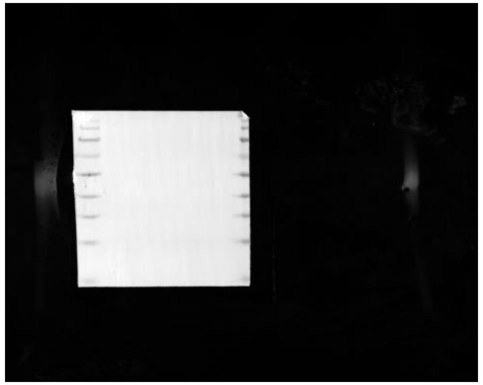
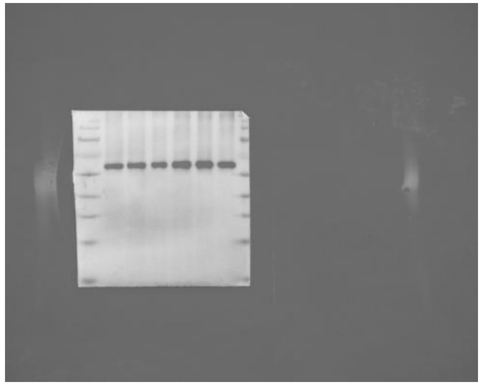

Western blot membrane of **AKT1 (~60 kDa)** protein detected with anti-AKT1 (ab32505; 1:1000; ab32505, Cambridge, US) antibody. Gel-separated proteins were transferred to PVDF membranes (0.45 μm pore size; Millipore, Billerice, USA) by semidry electroblotting (1.3 A,2.5V, 15 min). Membranes, incubated with a horseradish peroxidase-conjugated secondary antibody (BA1054; 1:5000–1:10000; Boater), were developed with Oriscience Supersensitive Kit (Oriscience Biotechnology). #Weight marker (molecular weight in kDa): Blue Plus IV Protein marker, 10 to 180 kDa; catalogue number: **R21223-V2**. Blot images, prior to the densitometry readings, were converted to grayscale with ImageJ (ImageJ, National Institutes of Health, Maryland, USA).

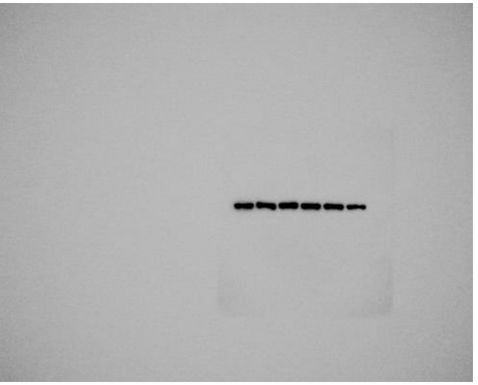
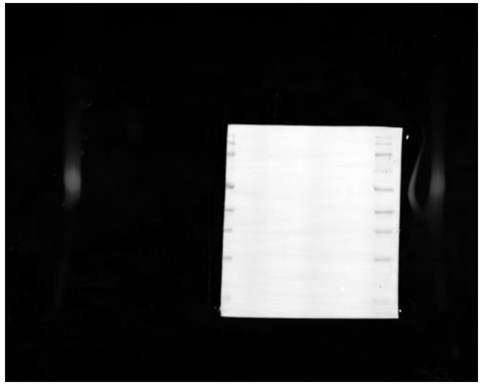
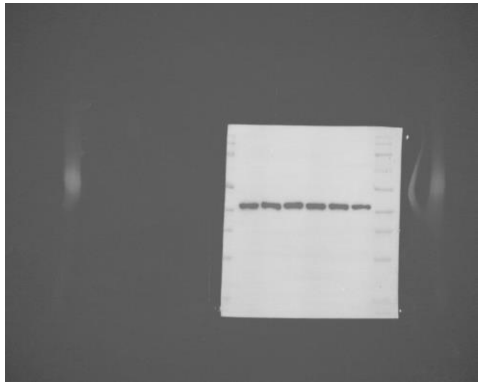

Western blot membrane of **β-actin** (~42 kDa) protein detected with anti-β-actin (BM0627; 1:8000; Boster, Wuhan, China) antibody. Gel-separated proteins were transferred to PVDF membranes (0.45 μm pore size; Millipore, Billerice, USA) by semidry electroblotting (1.3 A ,2.5V, 20 min). Membranes, incubated with a horseradish peroxidase-conjugated secondary antibody (BA1050; 1:5000–1:10000; Boster), were developed with Oriscience Supersensitive Kit (Oriscience Biotechnology). #Weight marker (molecular weight in kDa): Blue Plus IV Protein marker, 10 to 180 kDa; catalogue number: **R21223-V2**. Blot images, prior to the densitometry readings, were converted to grayscale with ImageJ (ImageJ, National Institutes of Health, Maryland, USA).

8-3、p-AKT/AKT (+LY294002）**figure5C**

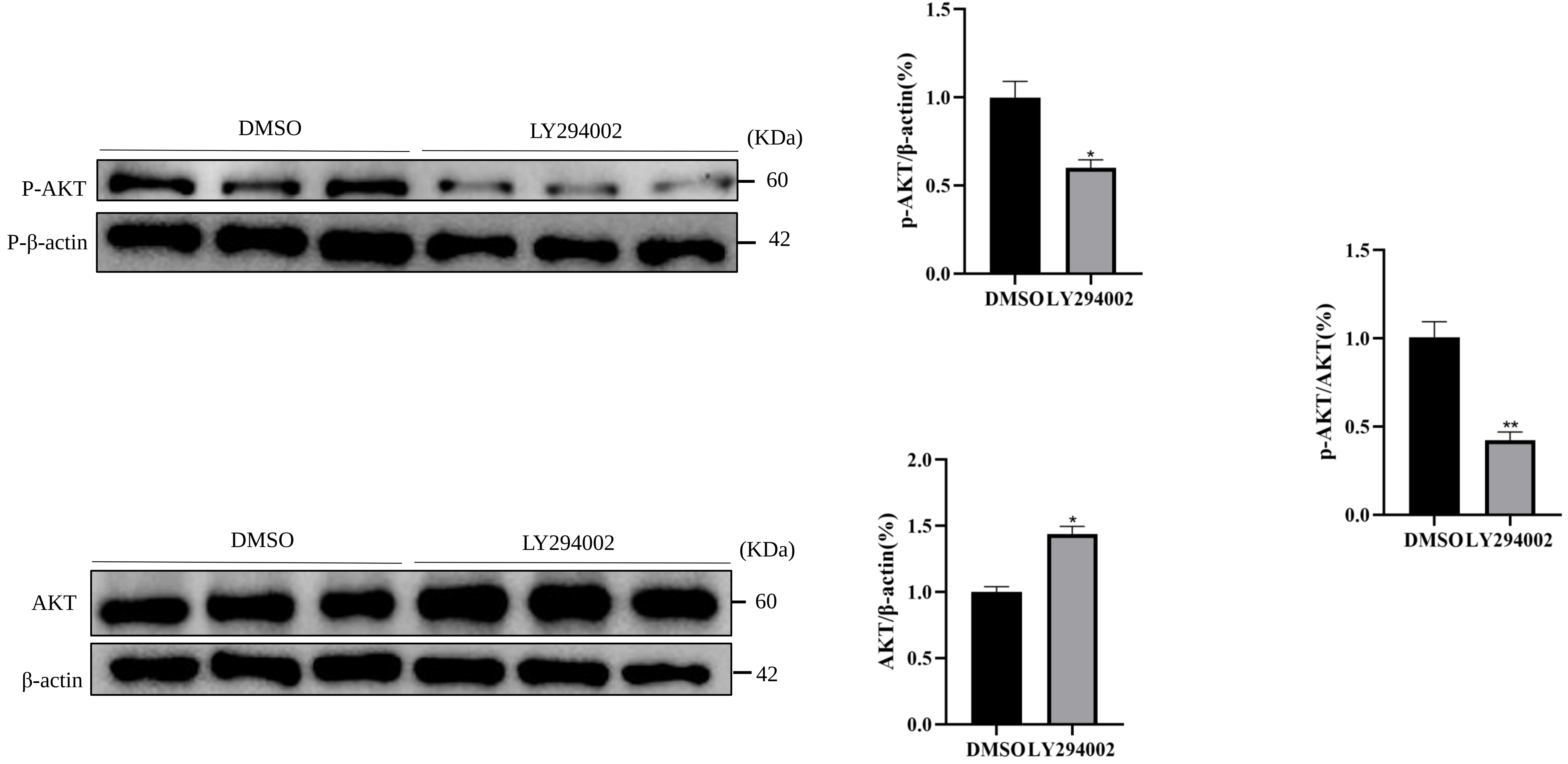

9、PPARγ(OE-PLIN5） **figure 6A**

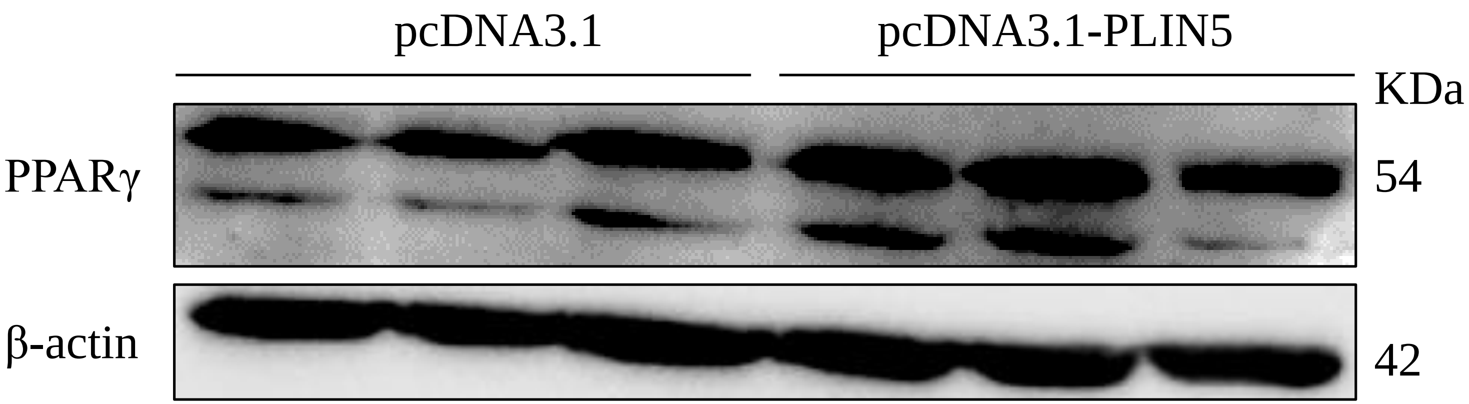

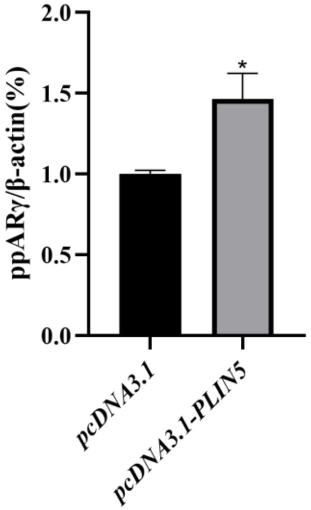

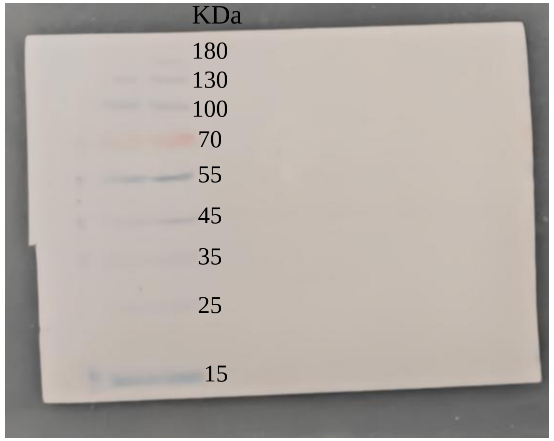

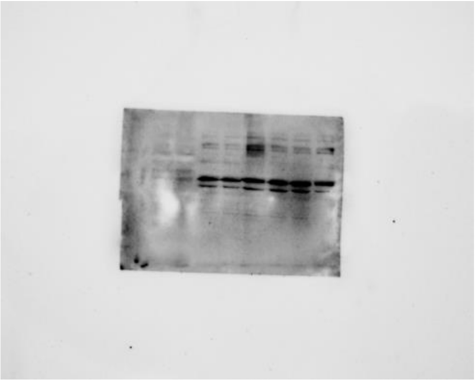

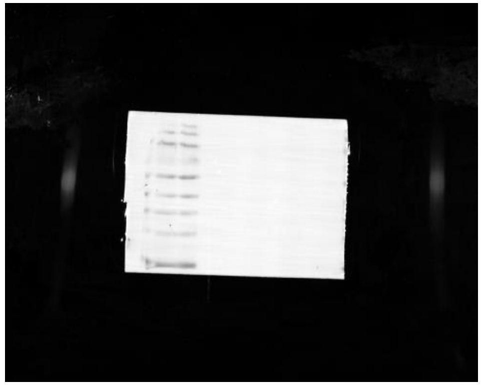

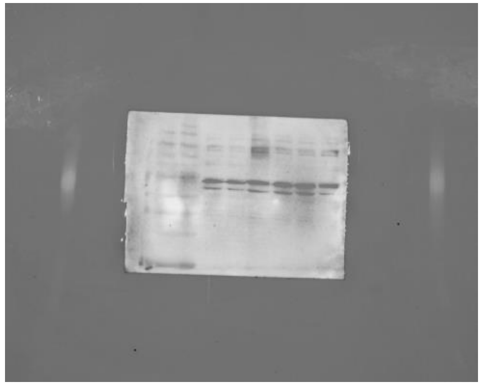

Western blot membrane of **PPARγ** (~54 kDa) protein detected with anti- PPARγ (16643-1-Ap; 1:8000; Proteintech, Wuhan, China) antibody. Gel-separated proteins were transferred to PVDF membranes (0.45 μm pore size; Millipore, Billerice, USA) by semidry electroblotting (1.3 A,2.5V, 15 min). Membranes, incubated with a horseradish peroxidase-conjugated secondary antibody (BA1054; 1:5000–1:10000; Boater), were developed with Oriscience Supersensitive Kit (Oriscience Biotechnology). #Weight marker (molecular weight in kDa): Blue Plus IV Protein marker, 10 to 180 kDa; catalogue number: **R21223-V2**. Blot images, prior to the densitometry readings, were converted to grayscale with ImageJ (ImageJ, National Institutes of Health, Maryland, USA).

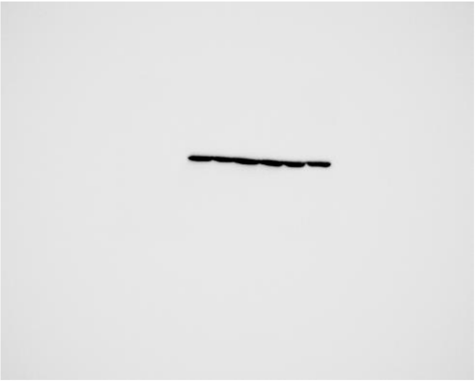

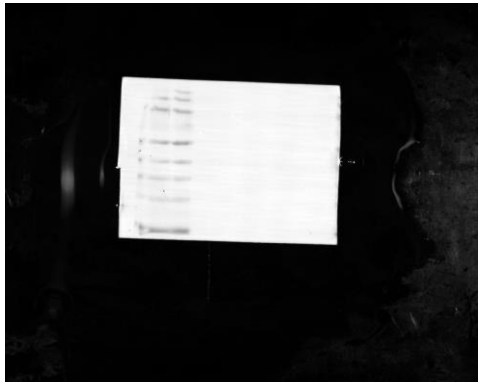

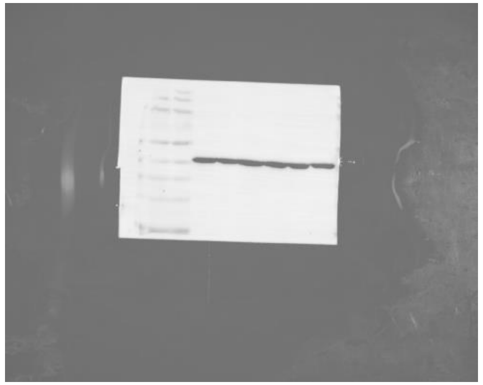

Western blot membrane of **β-actin** (~42 kDa) protein detected with anti-β-actin (BM0627; 1:8000; Boster, Wuhan, China) antibody. Gel-separated proteins were transferred to PVDF membranes (0.45 μm pore size; Millipore, Billerice, USA) by semidry electroblotting (1.3 A ,2.5V, 20 min). Membranes, incubated with a horseradish peroxidase-conjugated secondary antibody (BA1050; 1:5000–1:10000; Boster), were developed with Oriscience Supersensitive Kit (Oriscience Biotechnology). #Weight marker (molecular weight in kDa): Blue Plus IV Protein marker, 10 to 180 kDa; catalogue number: **R21223-V2**. Blot images, prior to the densitometry readings, were converted to grayscale with ImageJ (ImageJ, National Institutes of Health, Maryland, USA).

10、PPARγ(si-PLIN5） **figure6B**

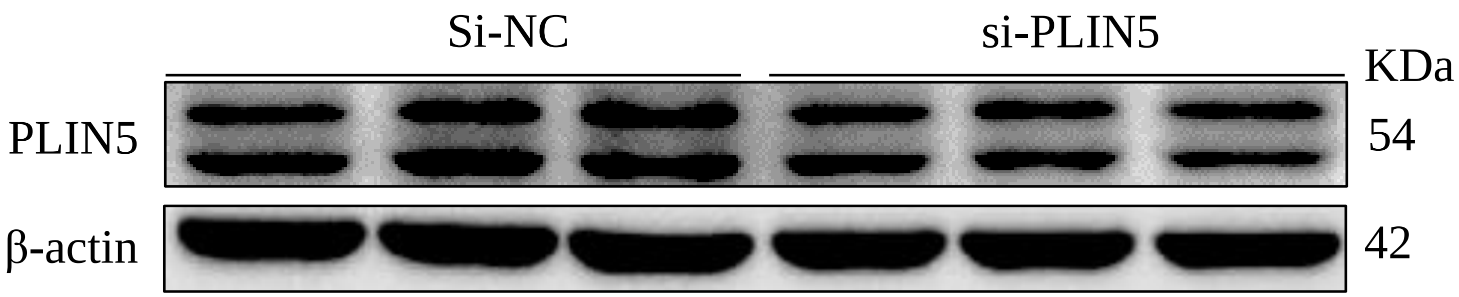

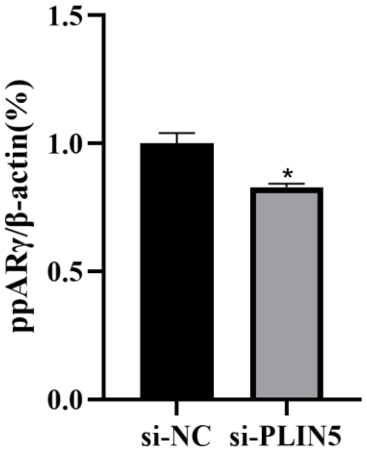

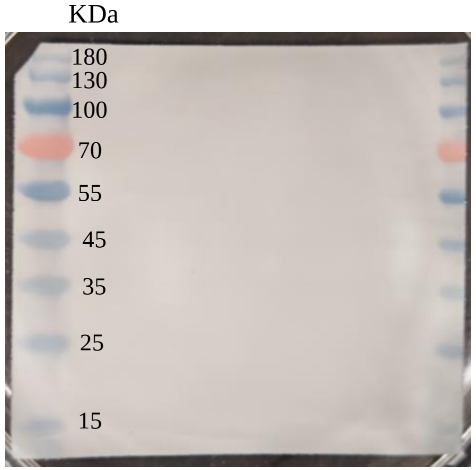

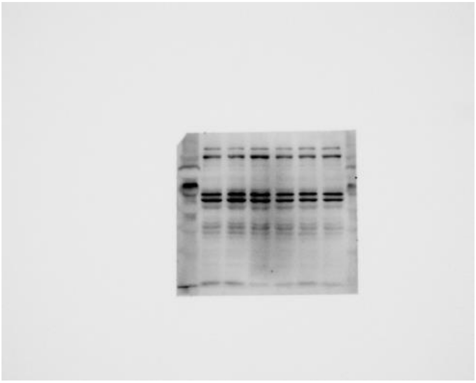
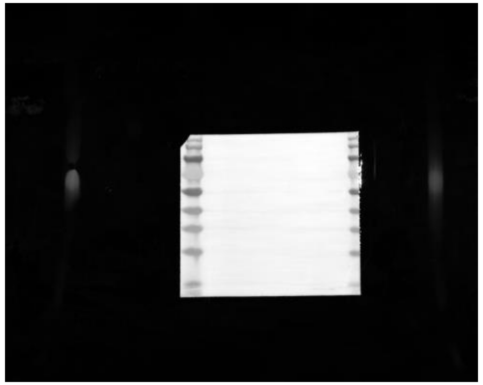
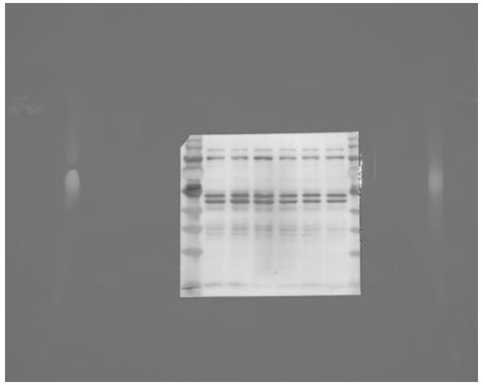

Western blot membrane of **PPARγ** (~54 kDa) protein detected with anti- PPARγ (16643-1-Ap; 1:8000; Proteintech, Wuhan, China) antibody. Gel-separated proteins were transferred to PVDF membranes (0.45 μm pore size; Millipore, Billerice, USA) by semidry electroblotting (1.3 A,2.5V, 15 min). Membranes, incubated with a horseradish peroxidase-conjugated secondary antibody (BA1054; 1:5000–1:10000; Boater), were developed with Oriscience Supersensitive Kit (Oriscience Biotechnology). #Weight marker (molecular weight in kDa): Blue Plus IV Protein marker, 10 to 180 kDa; catalogue number: **R21223-V2**. Blot images, prior to the densitometry readings, were converted to grayscale with ImageJ (ImageJ, National Institutes of Health, Maryland, USA).

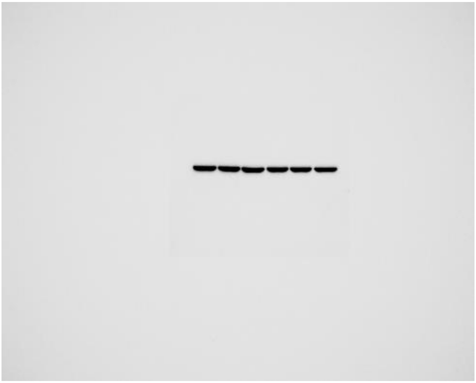
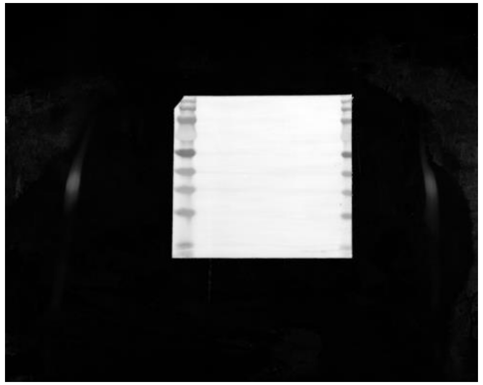
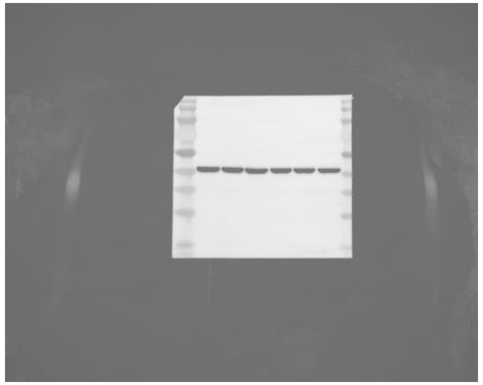

Western blot membrane of **β-actin** (~42 kDa) protein detected with anti-β-actin (BM0627; 1:8000; Boster, Wuhan, China) antibody. Gel-separated proteins were transferred to PVDF membranes (0.45 μm pore size; Millipore, Billerice, USA) by semidry electroblotting (1.3 A ,2.5V, 20 min). Membranes, incubated with a horseradish peroxidase-conjugated secondary antibody (BA1050; 1:5000–1:10000; Boster), were developed with Oriscience Supersensitive Kit (Oriscience Biotechnology). #Weight marker (molecular weight in kDa): Blue Plus IV Protein marker, 10 to 180 kDa; catalogue number: **R21223-V2**. Blot images, prior to the densitometry readings, were converted to grayscale with ImageJ (ImageJ, National Institutes of Health, Maryland, USA).

11、PPARγ(si- PPARγ ） **figure7A**

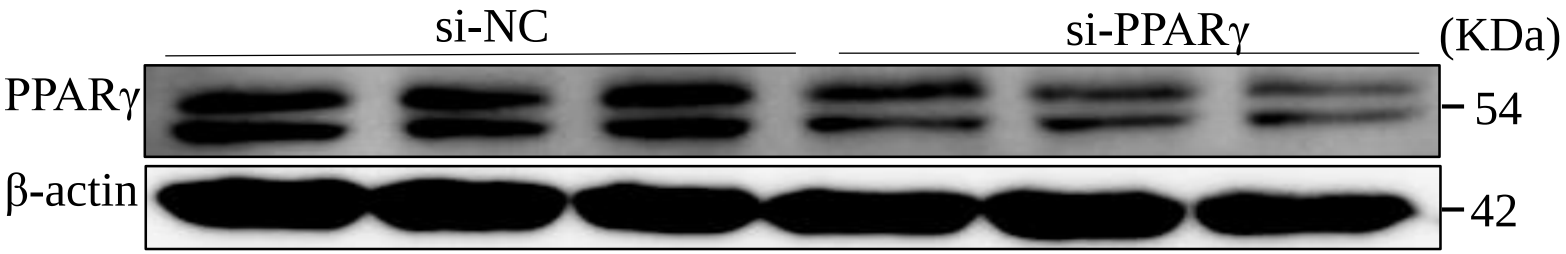

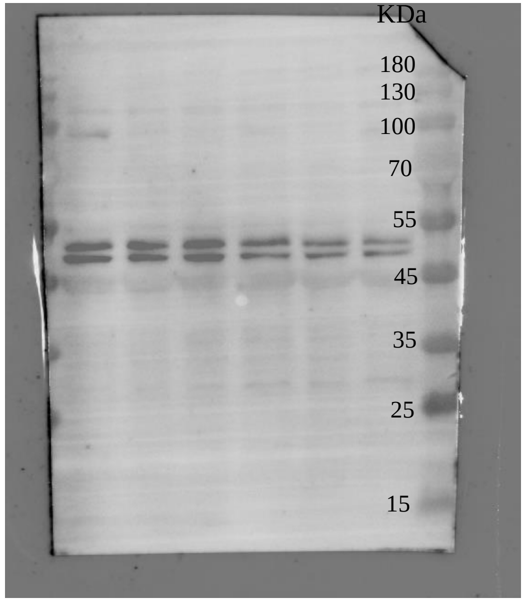

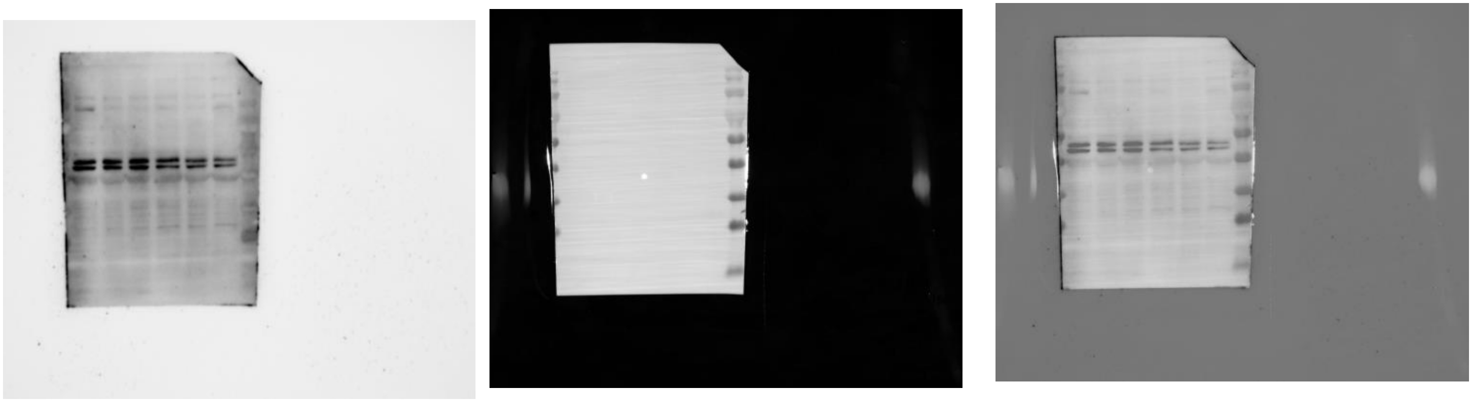

Western blot membrane of **PPARγ** (~54 kDa) protein detected with anti- PPARγ (16643-1-Ap; 1:8000; Proteintech, Wuhan, China) antibody. Gel-separated proteins were transferred to PVDF membranes (0.45 μm pore size; Millipore, Billerice, USA) by semidry electroblotting (1.3 A,2.5V, 15 min). Membranes, incubated with a horseradish peroxidase-conjugated secondary antibody (BA1054; 1:5000–1:10000; Boater), were developed with Oriscience Supersensitive Kit (Oriscience Biotechnology). #Weight marker (molecular weight in kDa): Blue Plus IV Protein marker, 10 to 180 kDa; catalogue number: **R21223-V2**. Blot images, prior to the densitometry readings, were converted to grayscale with ImageJ (ImageJ, National Institutes of Health, Maryland, USA).

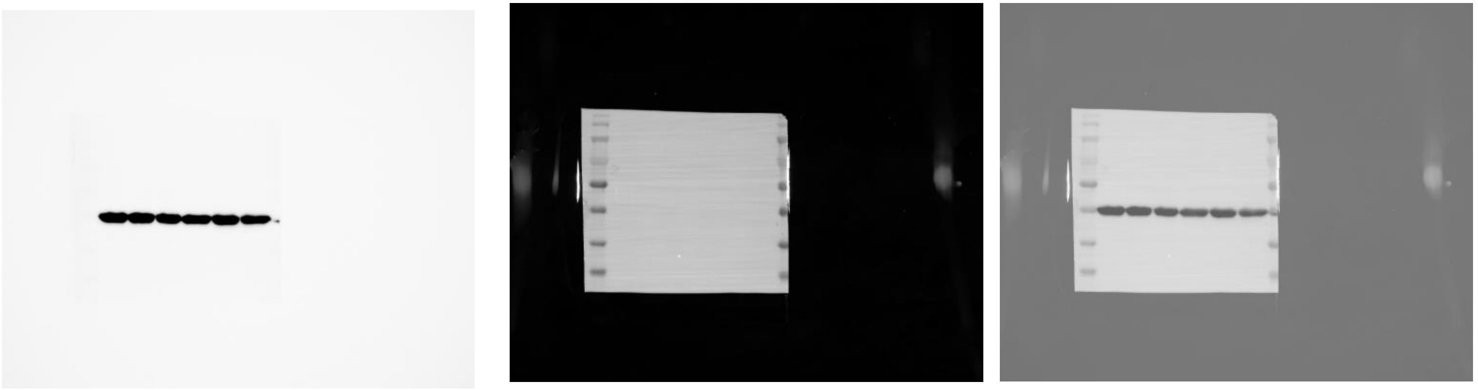

Western blot membrane of **β-actin** (~42 kDa) protein detected with anti-β-actin (BM0627; 1:8000; Boster, Wuhan, China) antibody. Gel-separated proteins were transferred to PVDF membranes (0.45 μm pore size; Millipore, Billerice, USA) by semidry electroblotting (1.3 A ,2.5V, 20 min). Membranes, incubated with a horseradish peroxidase-conjugated secondary antibody (BA1050; 1:5000–1:10000; Boster), were developed with Oriscience Supersensitive Kit (Oriscience Biotechnology). #Weight marker (molecular weight in kDa): Blue Plus IV Protein marker, 10 to 180 kDa; catalogue number: **R21223-V2**. Blot images, prior to the densitometry readings, were converted to grayscale with ImageJ (ImageJ, National Institutes of Health, Maryland, USA).

12-1、P-AKT(+GW9662) **figure7H**

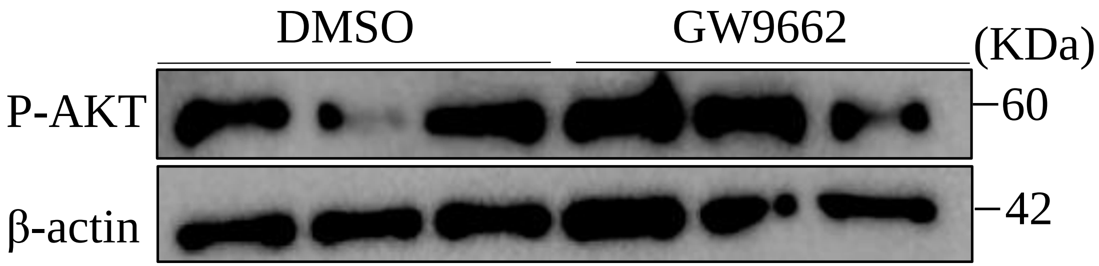

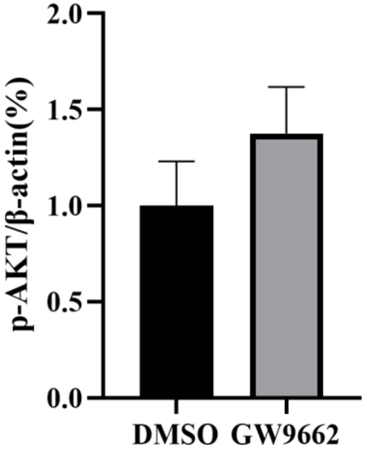

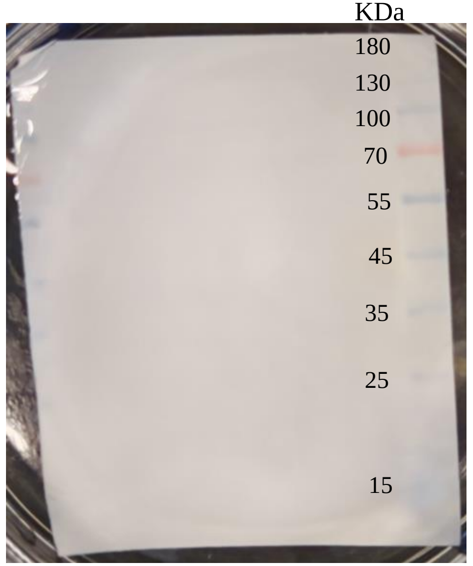

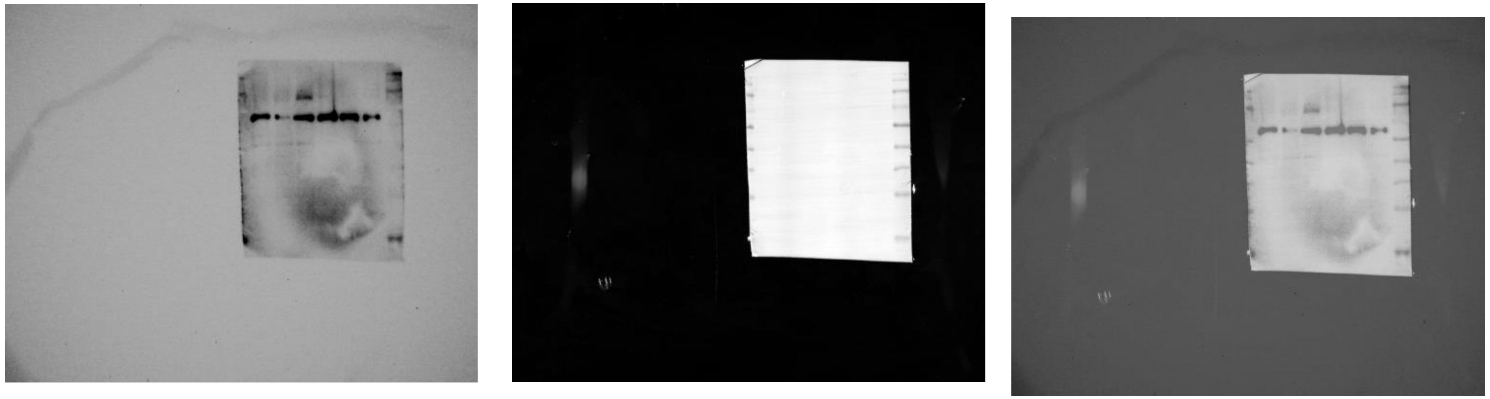

Western blot membrane of **AKT1 (~60 kDa)** protein detected with anti-AKT1 (ab32505; 1:1000; ab32505, Cambridge, US) antibody. Gel-separated proteins were transferred to PVDF membranes (0.45 μm pore size; Millipore, Billerice, USA) by semidry electroblotting (1.3 A,2.5V, 15 min). Membranes, incubated with a horseradish peroxidase-conjugated secondary antibody (BA1054; 1:5000–1:10000; Boater), were developed with Oriscience Supersensitive Kit (Oriscience Biotechnology). #Weight marker (molecular weight in kDa): Blue Plus IV Protein marker, 10 to 180 kDa; catalogue number: **R21223-V2**. Blot images, prior to the densitometry readings, were converted to grayscale with ImageJ (ImageJ, National Institutes of Health, Maryland, USA).

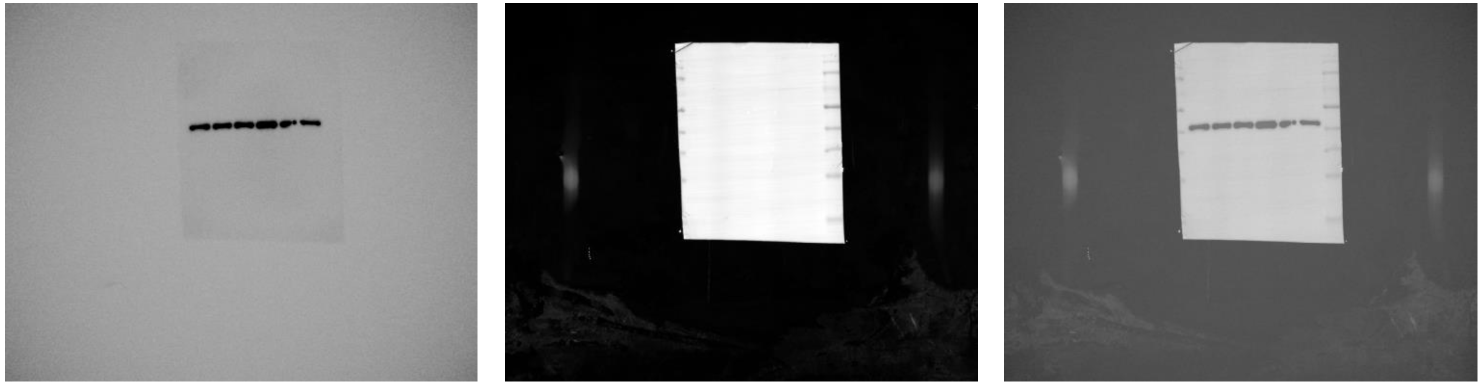

Western blot membrane of **β-actin** (~42 kDa) protein detected with anti-β-actin (BM0627; 1:8000; Boster, Wuhan, China) antibody. Gel-separated proteins were transferred to PVDF membranes (0.45 μm pore size; Millipore, Billerice, USA) by semidry electroblotting (1.3 A ,2.5V, 20 min). Membranes, incubated with a horseradish peroxidase-conjugated secondary antibody (BA1050; 1:5000–1:10000; Boster), were developed with Oriscience Supersensitive Kit (Oriscience Biotechnology). #Weight marker (molecular weight in kDa): Blue Plus IV Protein marker, 10 to 180 kDa; catalogue number: **R21223-V2**. Blot images, prior to the densitometry readings, were converted to grayscale with ImageJ (ImageJ, National Institutes of Health, Maryland, USA).

12-2、AKT(+GW9662) **figure7H**

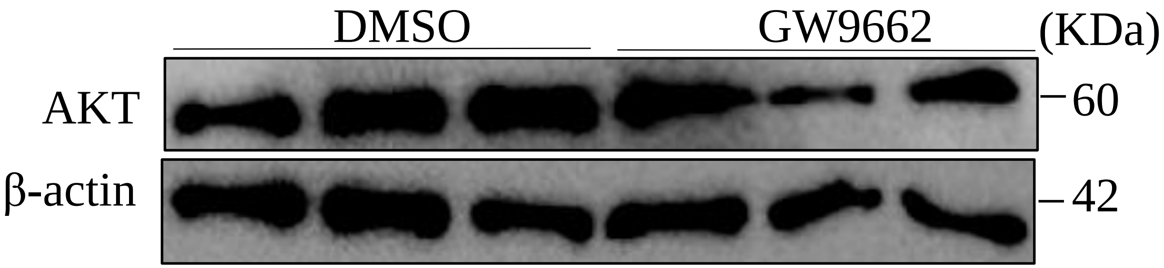

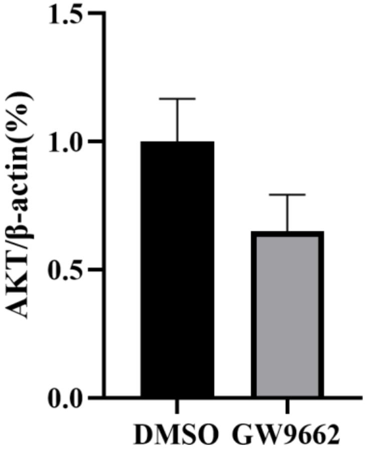

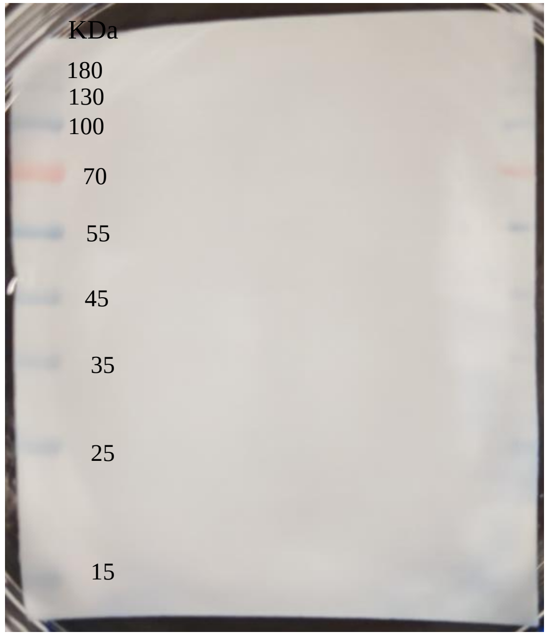

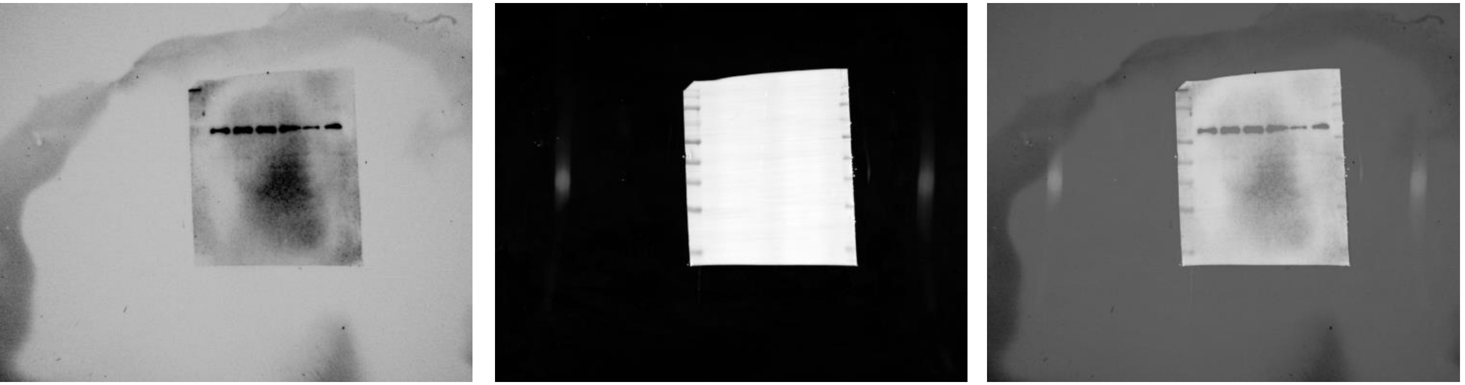

Western blot membrane of **p-AKT (~60 kDa)** protein detected with anti-p-AKT1-Ser473 (4060; 1:2000; Cell Signaling Technology, Massachusetts, USA) antibody. Gel-separated proteins were transferred to PVDF membranes (0.45 μm pore size; Millipore, Billerice, USA) by semidry electroblotting (1.3 A,2.5V, 15 min). Membranes, incubated with a horseradish peroxidase-conjugated secondary antibody (BA1054; 1:5000–1:10000; Boater), were developed with Oriscience Supersensitive Kit (Oriscience Biotechnology). #Weight marker (molecular weight in kDa): Blue Plus IV Protein marker, 10 to 180 kDa; catalogue number: **R21223-V2**. Blot images, prior to the densitometry readings, were converted to grayscale with ImageJ (ImageJ, National Institutes of Health, Maryland, USA).

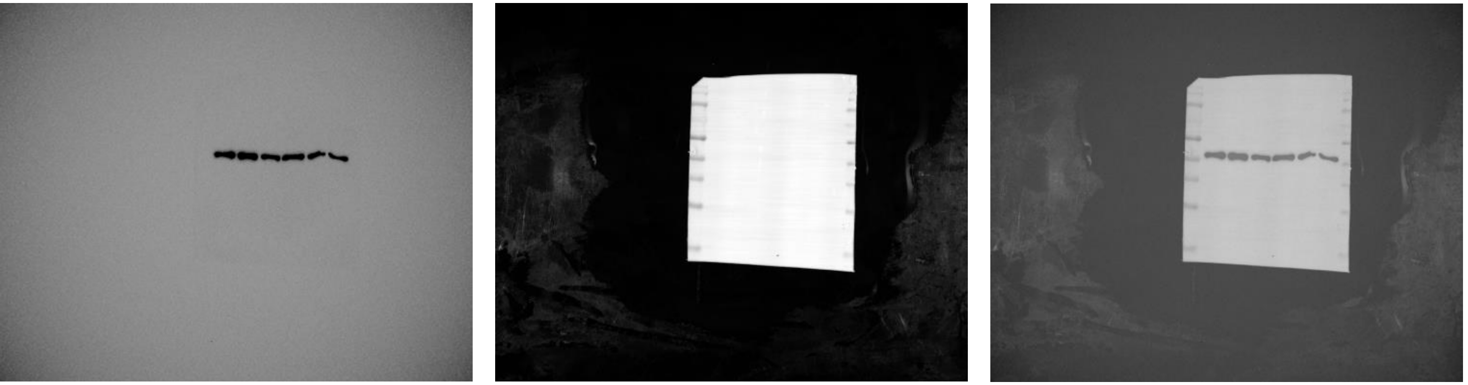

Western blot membrane of **β-actin** (~42 kDa) protein detected with anti-β-actin (BM0627; 1:8000; Boster, Wuhan, China) antibody. Gel-separated proteins were transferred to PVDF membranes (0.45 μm pore size; Millipore, Billerice, USA) by semidry electroblotting (1.3 A ,2.5V, 20 min). Membranes, incubated with a horseradish peroxidase-conjugated secondary antibody (BA1050; 1:5000–1:10000; Boster), were developed with Oriscience Supersensitive Kit (Oriscience Biotechnology). #Weight marker (molecular weight in kDa): Blue Plus IV Protein marker, 10 to 180 kDa; catalogue number: **R21223-V2**. Blot images, prior to the densitometry readings, were converted to grayscale with ImageJ (ImageJ, National Institutes of Health, Maryland, USA).

12-3、p-AKT/AKT (+GW9662) **figure7H**

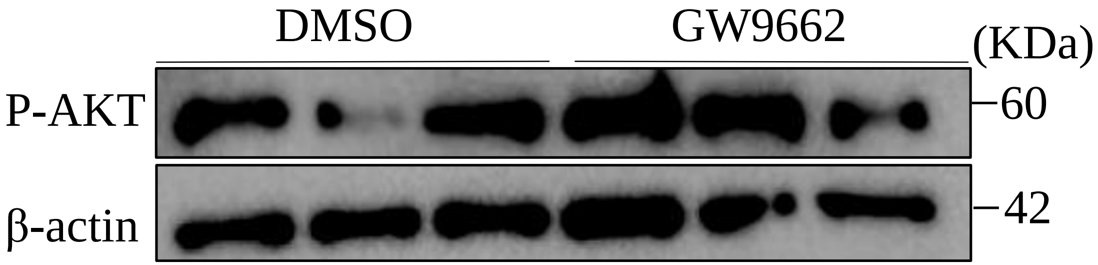

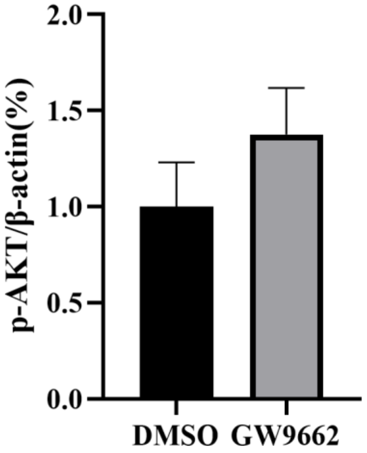

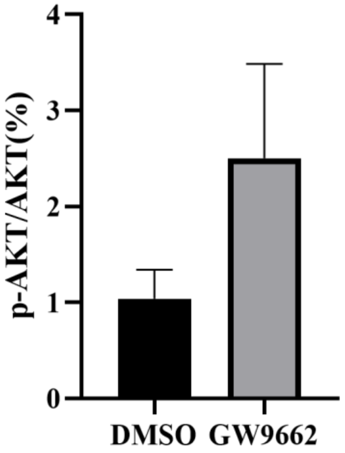

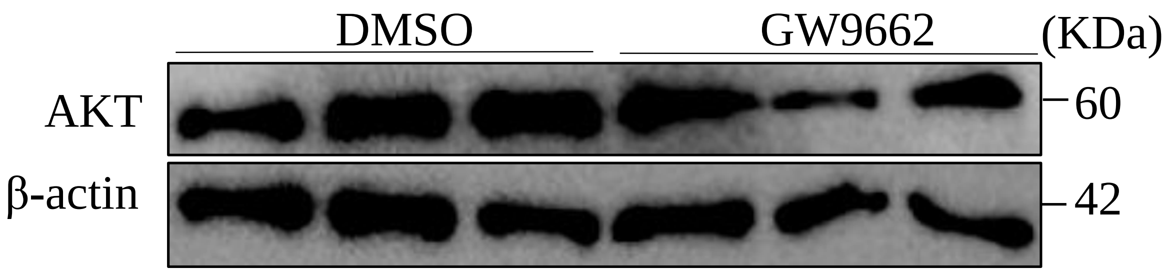

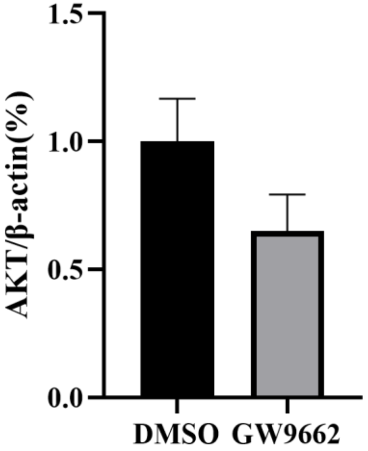

13-1、p-p38(OE-PLIN5） **figureS3C**

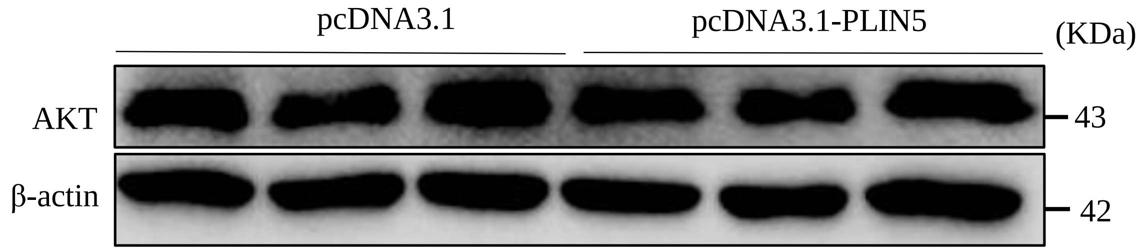

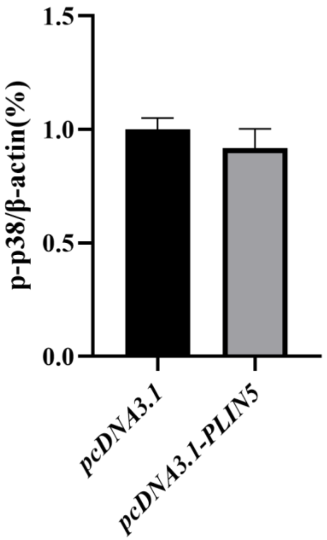

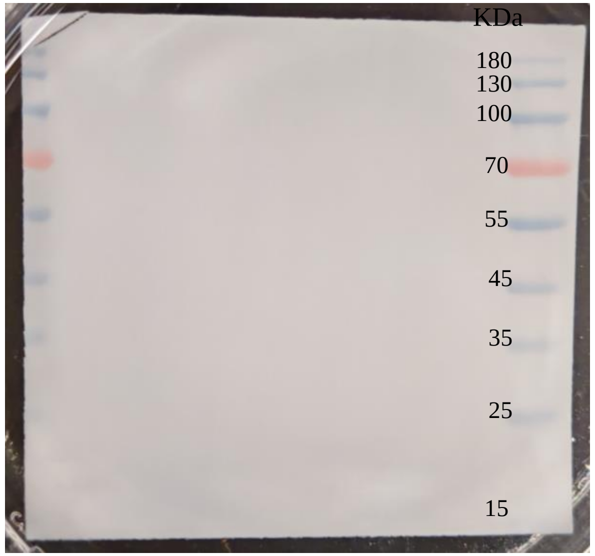

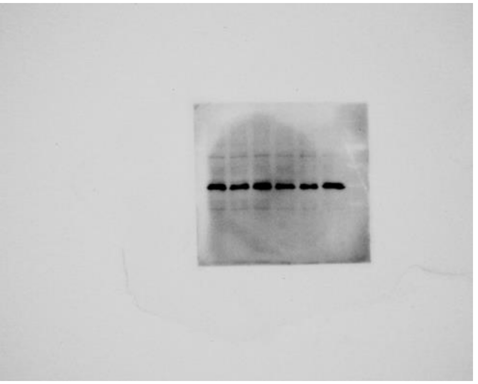

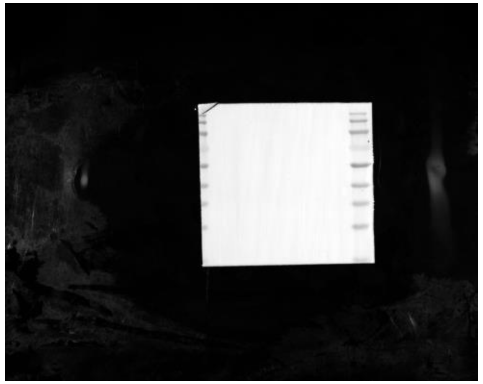

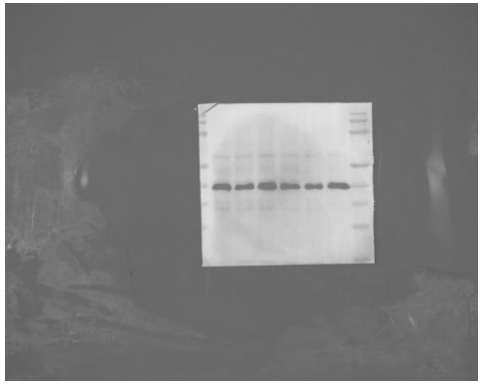

Western blot membrane of **p-p38 (~43 kDa)** protein detected with anti-p-p38-MAPK (3285S; 1:1000; Cell Signaling Technology, Massachusetts, USA) antibody.Gel-separated proteins were transferred to PVDF membranes (0.45 µm pore size; Millipore, Billerice, USA) by semidry electroblotting (1.3 A,2.5V, 15 min). Membranes, incubated with a horseradish peroxidase-conjugated secondary antibody (BA1054; 1:5000–1:10000; Boater), were developed with Oriscience Supersensitive Kit (Oriscience Biotechnology). #Weight marker (molecular weight in kDa): Blue Plus IV Protein marker, 10 to 180 kDa; catalogue number: **R21223-V2**. Blot images, prior to the densitometry readings, were converted to grayscale with ImageJ (ImageJ, National Institutes of Health, Maryland, USA).

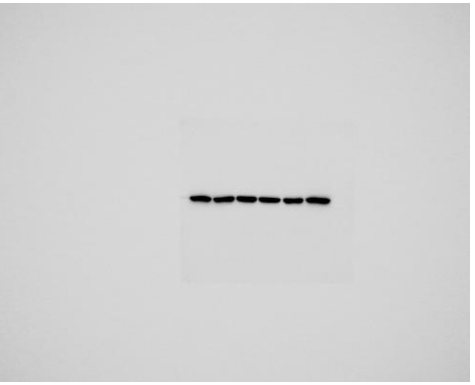

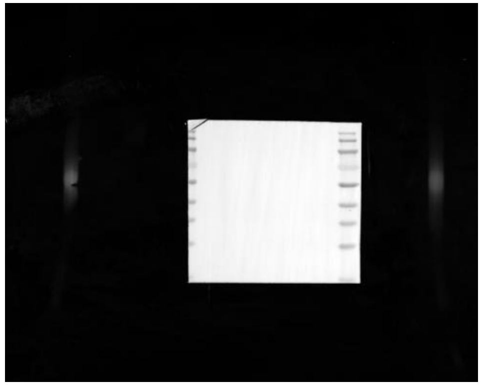

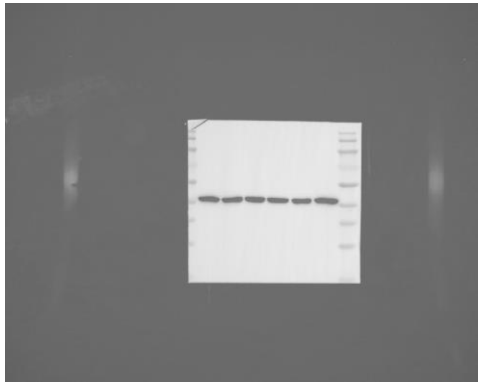

Western blot membrane of **β-actin** (~42 kDa) protein detected with anti-β-actin (BM0627; 1:8000; Boster, Wuhan, China) antibody. Gel-separated proteins were transferred to PVDF membranes (0.45 µm pore size; Millipore, Billerice, USA) by semidry electroblotting (1.3 A ,2.5V, 20 min). Membranes, incubated with a horseradish peroxidase-conjugated secondary antibody (BA1050; 1:5000–1:10000; Boster), were developed with Oriscience Supersensitive Kit (Oriscience Biotechnology). #Weight marker (molecular weight in kDa): Blue Plus IV Protein marker, 10 to 180 kDa; catalogue number: **R21223-V2**. Blot images, prior to the densitometry readings, were converted to grayscale with ImageJ (ImageJ, National Institutes of Health, Maryland, USA).

13-2、p-38(OE-PLIN5) **figureS3C**

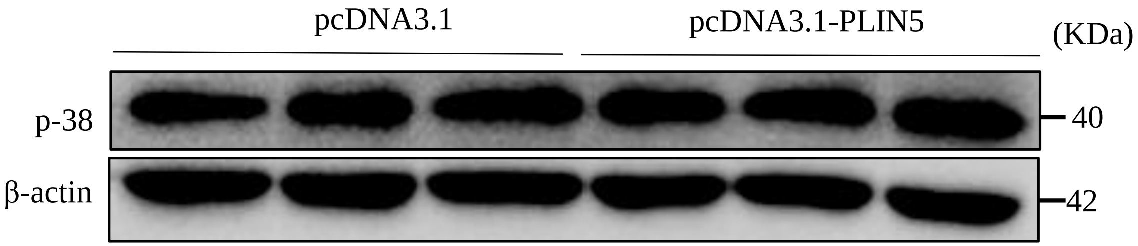

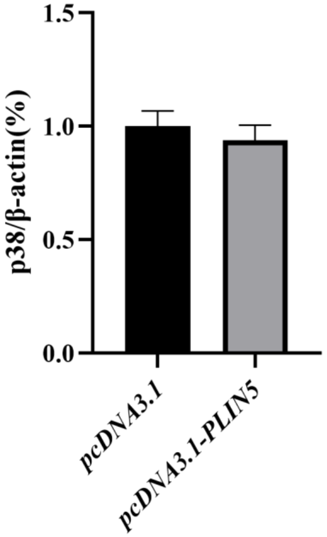

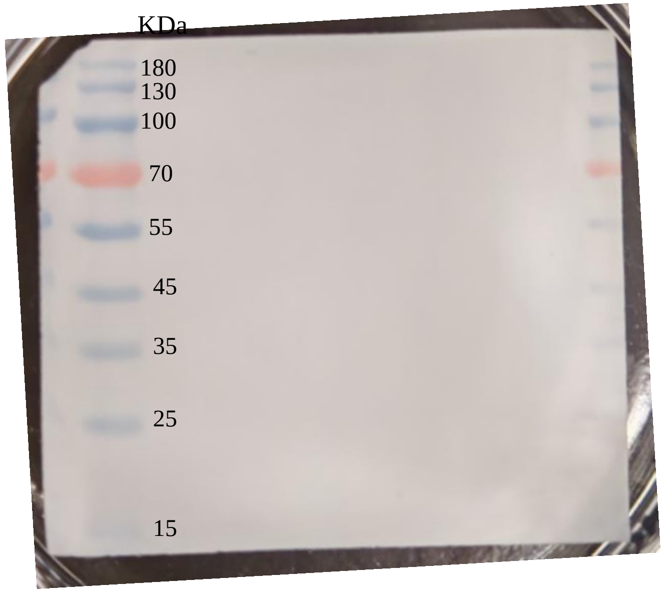

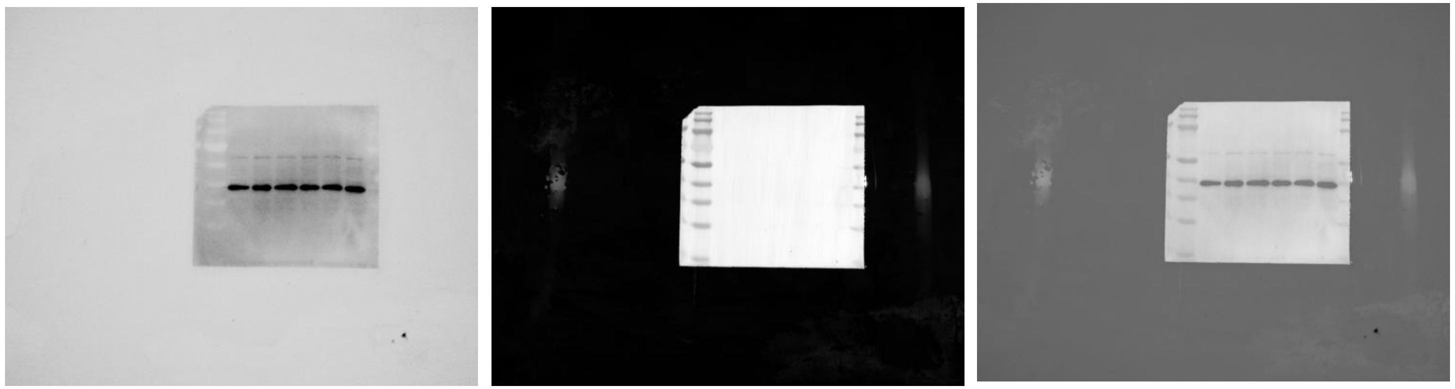

Western blot membrane of **p-38 (~40 kDa)** protein detected with anti- p38-MAPK(4; 1:1000; Cell Signaling Technology, Massachusetts, USA) antibody. Gel-separated proteins were transferred to PVDF membranes (0.45 µm pore size; Millipore, Billerice, USA) by semidry electroblotting (1.3 A,2.5V, 15 min). Membranes, incubated with a horseradish peroxidase-conjugated secondary antibody (BA1054; 1:5000–1:10000; Boater), were developed with Oriscience Supersensitive Kit (Oriscience Biotechnology). #Weight marker (molecular weight in kDa): Blue Plus IV Protein marker, 10 to 180 kDa; catalogue number: **R21223-V2**. Blot images, prior to the densitometry readings, were converted to grayscale with ImageJ (ImageJ, National Institutes of Health, Maryland, USA).

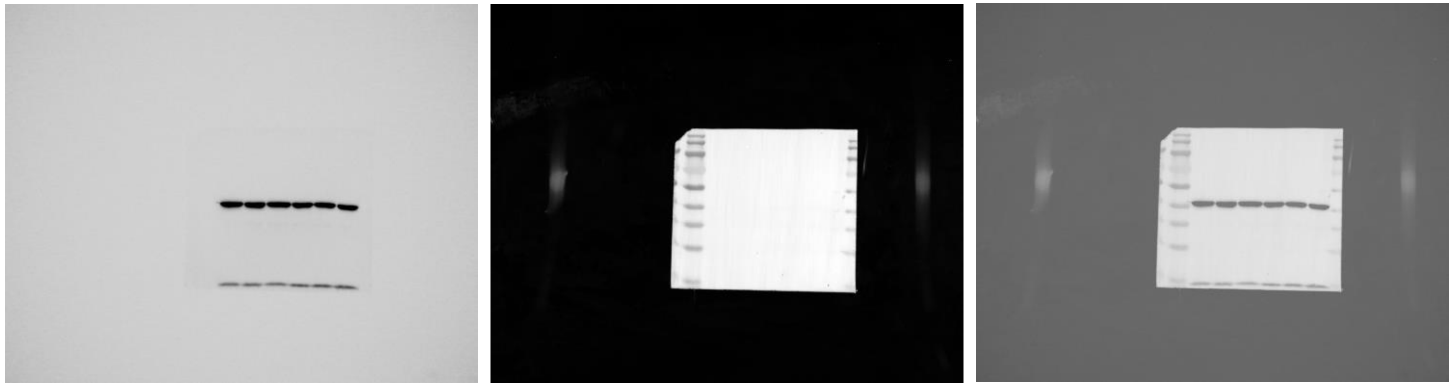

Western blot membrane of **β-actin** (~42 kDa) protein detected with anti-β-actin (BM0627; 1:8000; Boster, Wuhan, China) antibody. Gel-separated proteins were transferred to PVDF membranes (0.45 µm pore size; Millipore, Billerice, USA) by semidry electroblotting (1.3 A ,2.5V, 20 min). Membranes, incubated with a horseradish peroxidase-conjugated secondary antibody (BA1050; 1:5000–1:10000; Boster), were developed with Oriscience Supersensitive Kit (Oriscience Biotechnology). #Weight marker (molecular weight in kDa): Blue Plus IV Protein marker, 10 to 180 kDa; catalogue number: **R21223-V2**. Blot images, prior to the densitometry readings, were converted to grayscale with ImageJ (ImageJ, National Institutes of Health, Maryland, USA).

13-3、p-p38/p38 (OE-PLIN5) **figureS3C**

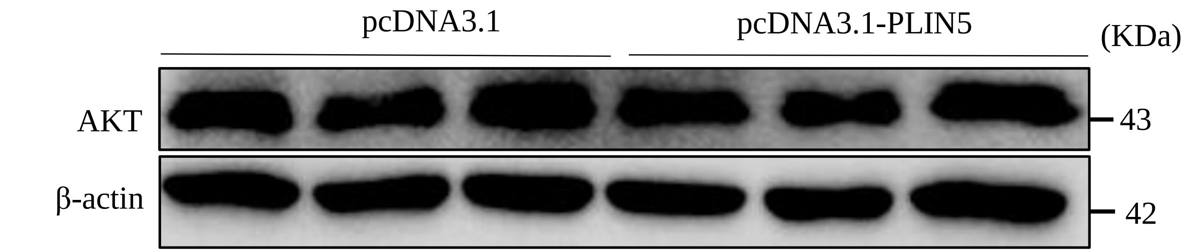

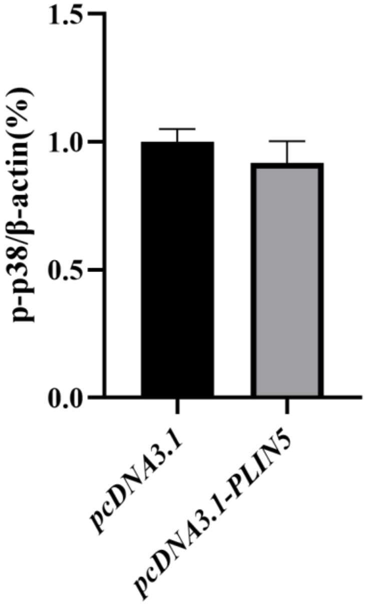

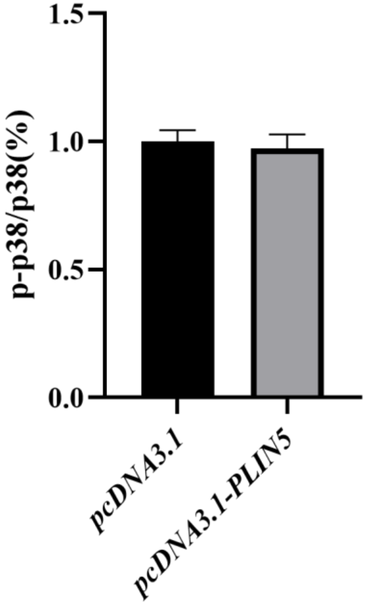

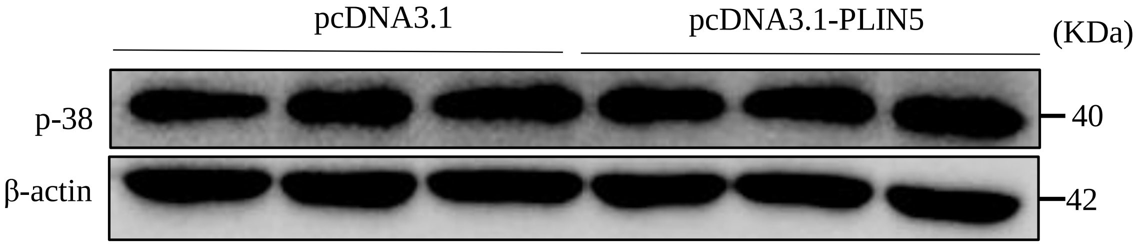

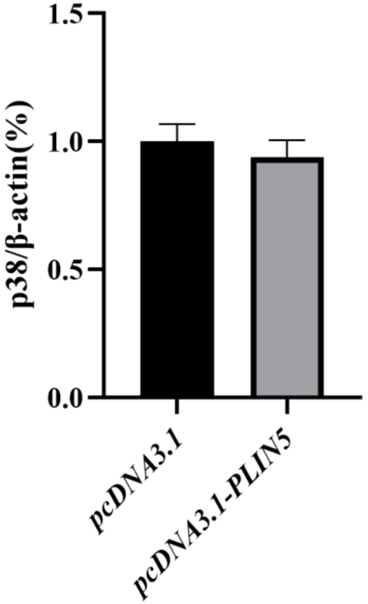

14-1、 p-AKT(si-PPARγ）**figureS5C**

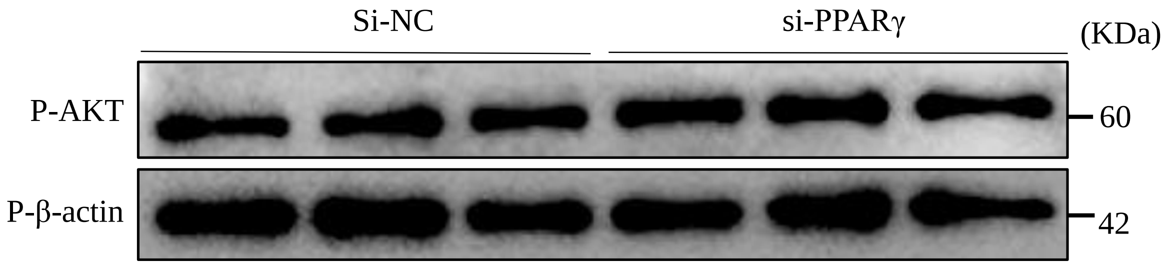

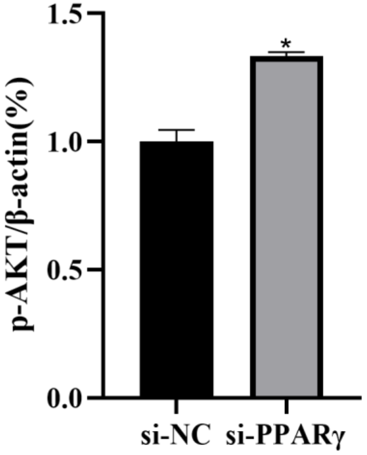

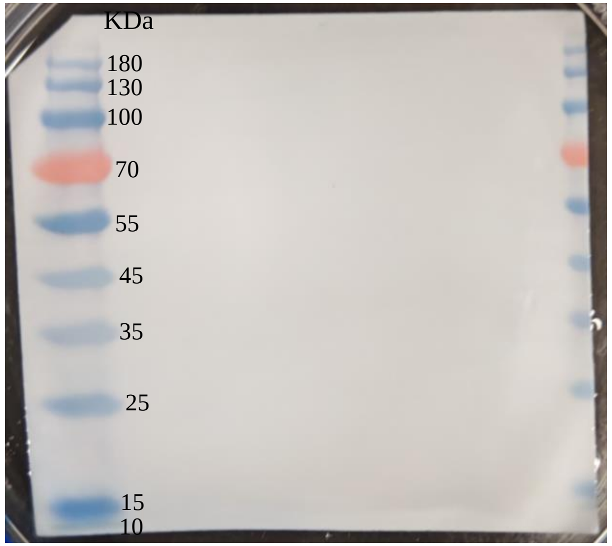

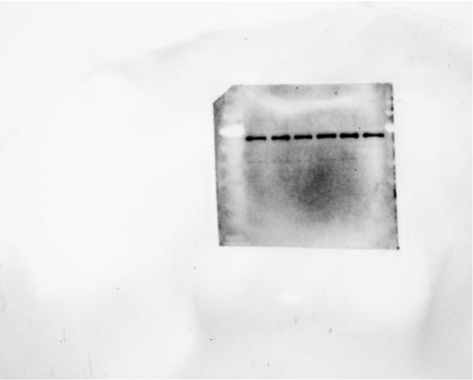

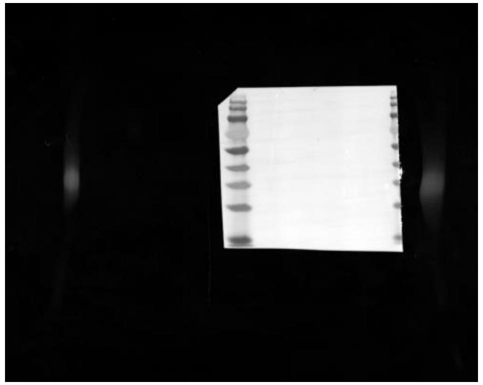

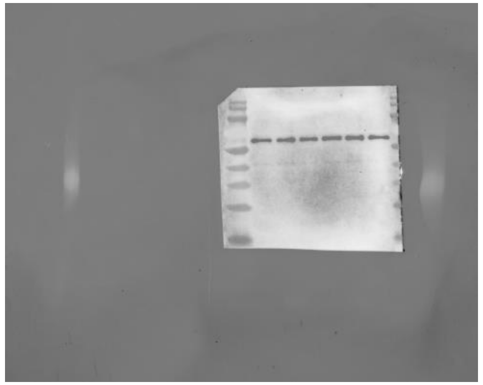

Western blot membrane of **p-AKT (~60 kDa)** protein detected with anti-p-AKT1-Ser473 (4060; 1:2000; Cell Signaling Technology, Massachusetts, USA) antibody. Gel-separated proteins were transferred to PVDF membranes (0.45 µm pore size; Millipore, Billerice, USA) by semidry electroblotting (1.3 A,2.5V, 15 min). Membranes, incubated with a horseradish peroxidase-conjugated secondary antibody (BA1054; 1:5000–1:10000; Boater), were developed with Oriscience Supersensitive Kit (Oriscience Biotechnology). #Weight marker (molecular weight in kDa): Blue Plus IV Protein marker, 10 to 180 kDa; catalogue number: **R21223-V2**. Blot images, prior to the densitometry readings, were converted to grayscale with ImageJ (ImageJ, National Institutes of Health, Maryland, USA).

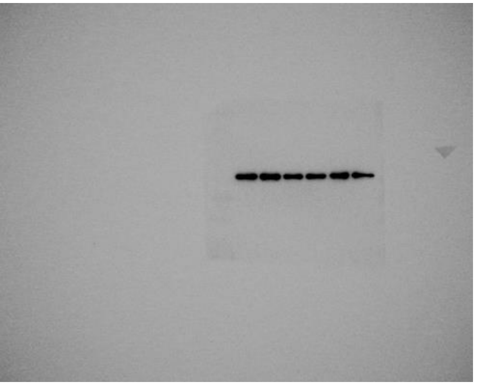

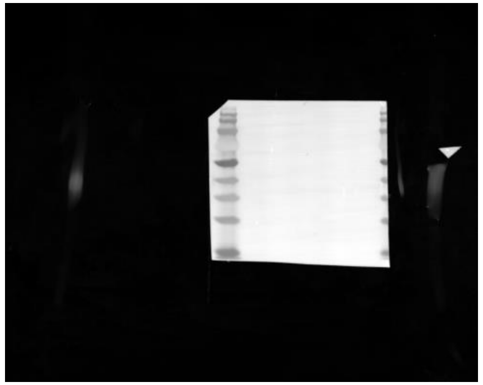

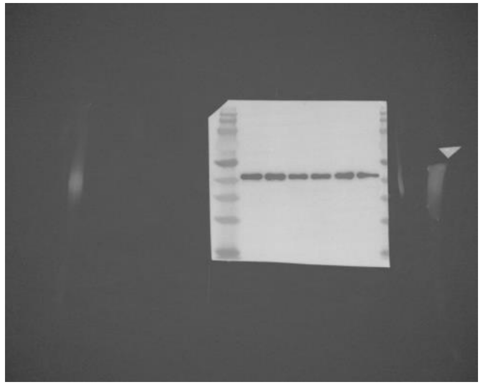

Western blot membrane of **β-actin** (~42 kDa) protein detected with anti-β-actin (BM0627; 1:8000; Boster, Wuhan, China) antibody. Gel-separated proteins were transferred to PVDF membranes (0.45 µm pore size; Millipore, Billerice, USA) by semidry electroblotting (1.3 A ,2.5V, 20 min). Membranes, incubated with a horseradish peroxidase-conjugated secondary antibody (BA1050; 1:5000–1:10000; Boster), were developed with Oriscience Supersensitive Kit (Oriscience Biotechnology). #Weight marker (molecular weight in kDa): Blue Plus IV Protein marker, 10 to 180 kDa; catalogue number: **R21223-V2**. Blot images, prior to the densitometry readings, were converted to grayscale with ImageJ (ImageJ, National Institutes of Health, Maryland, USA).

14-2、AKT(si-PPARγ）**figureS5C**

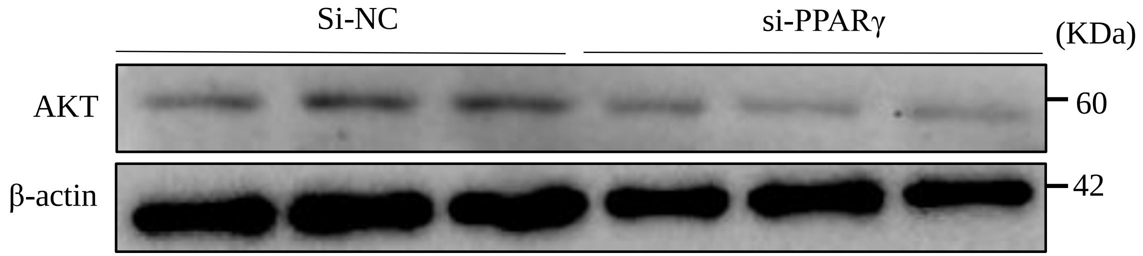

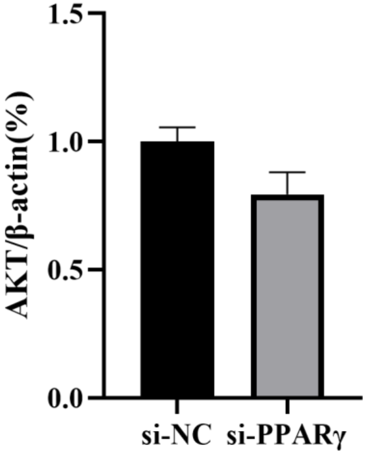

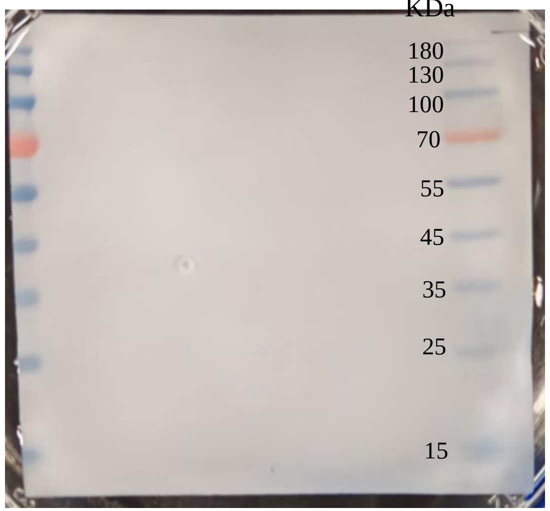

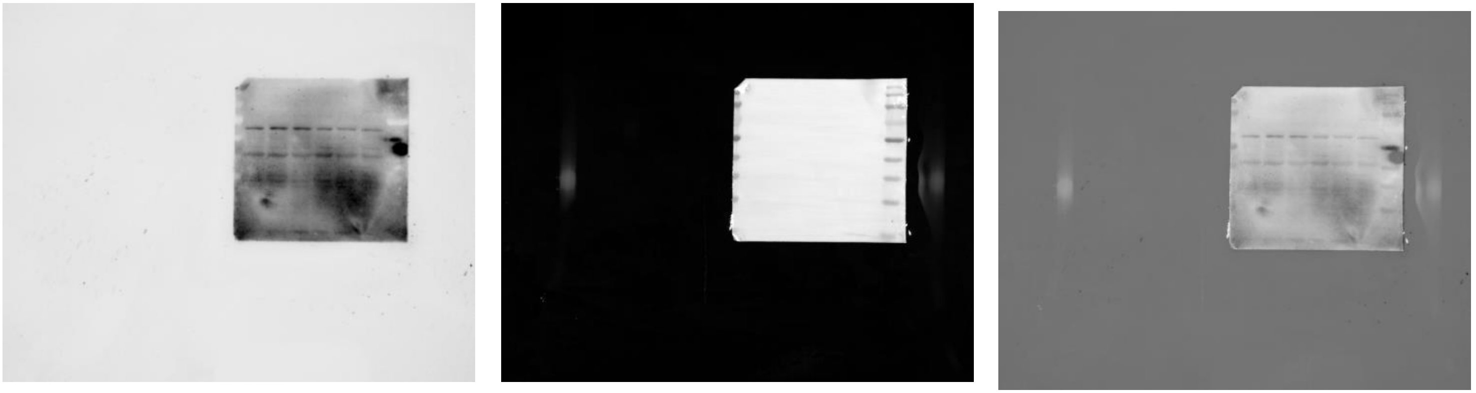

Western blot membrane of **AKT1 (~60 kDa)** protein detected with anti-AKT1 (ab32505; 1:1000; ab32505, Cambridge, US) antibody. Gel-separated proteins were transferred to PVDF membranes (0.45 μm pore size; Millipore, Billerice, USA) by semidry electroblotting (1.3 A,2.5V, 15 min). Membranes, incubated with a horseradish peroxidase-conjugated secondary antibody (BA1054; 1:5000–1:10000; Boater), were developed with Oriscience Supersensitive Kit (Oriscience Biotechnology). #Weight marker (molecular weight in kDa): Blue Plus IV Protein marker, 10 to 180 kDa; catalogue number: **R10519**. Blot images, prior to the densitometry readings, were converted to grayscale with ImageJ (ImageJ, National Institutes of Health, Maryland, USA).

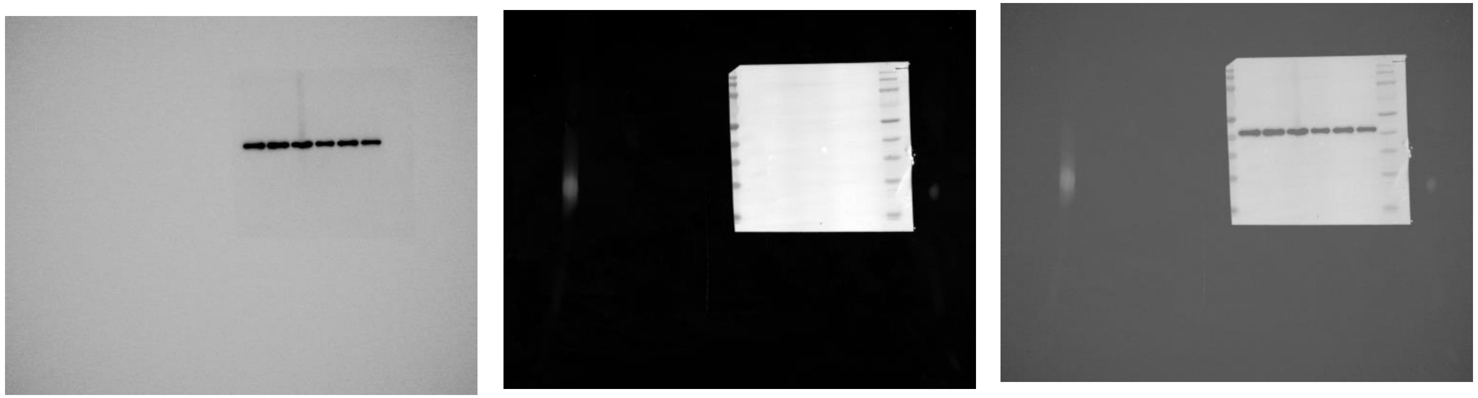

Western blot membrane of **β-actin** (~42 kDa) protein detected with anti-β-actin (BM0627; 1:8000; Boster, Wuhan, China) antibody. Gel-separated proteins were transferred to PVDF membranes (0.45 μm pore size; Millipore, Billerice, USA) by semidry electroblotting (1.3 A ,2.5V, 20 min). Membranes, incubated with a horseradish peroxidase-conjugated secondary antibody (BA1050; 1:5000–1:10000; Boster), were developed with Oriscience Supersensitive Kit (Oriscience Biotechnology). #Weight marker (molecular weight in kDa): Blue Plus IV Protein marker, 10 to 180 kDa; catalogue number: **R10519**. Blot images, prior to the densitometry readings, were converted to grayscale with ImageJ (ImageJ, National Institutes of Health, Maryland, USA).

14-3、p-AKT/AKT (si-PPARγ）**figureS5C**

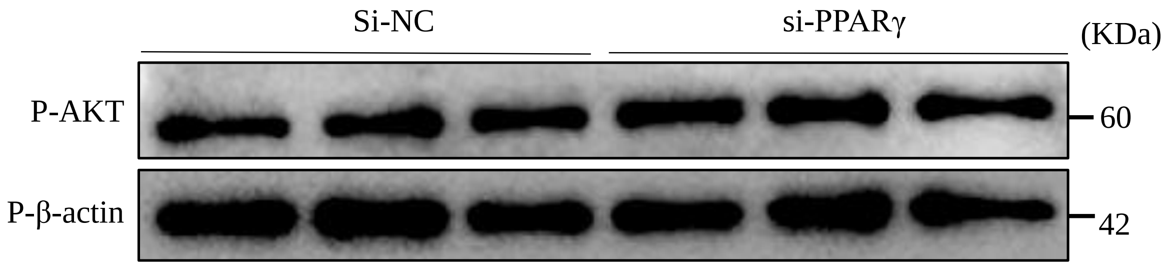

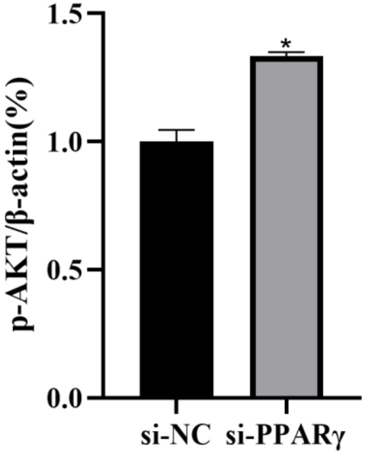

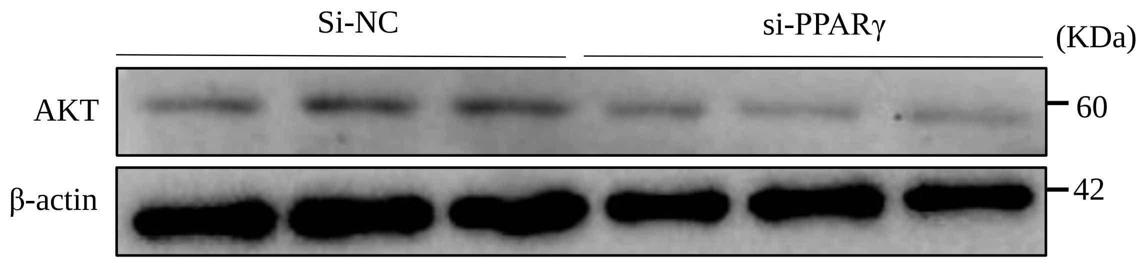

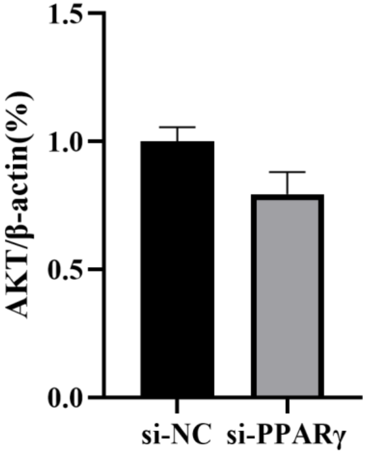

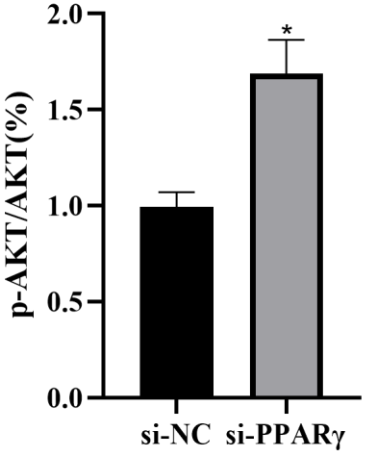

Supplement: Supplementary file 1 [file biology-14-01547-s001.zip › Supplementary figure S8.pdf]
