# Supplementary material for: PLIN5 Promotes Lipid Reconstitution in Goat Intramuscular Fat via the PPARγ Signaling Pathway
Source: Biology (Basel). 2025 Nov 4;14(11):1547. doi: 10.3390/biology14111547 (PMC12649955; doi:10.3390/biology14111547)
Supplement: Supplementary file 1 [file biology-14-01547-s001.zip › Supplementary figureS1-S6.pdf]

Supplementary figures

PLIN5 Promotes Lipid Reconstitution in Goat Intra-muscular Fat via the PPARγ Signaling Pathway

Yuhan Dai [1], Yuling Yang[1], Haiyang Li[1], YingGui, Wang[1] Yong Wang [1,2], Yaqiu Lin [1,2], Hua Xiang[1, 2], Lian Huang[1], Zhanyu Du[1], Changhui Zhang[1] *, and Jiangjiang Zhu[1, 2]*

***Correspondence:**
Changhui Zhang, zhangchanghui0074@163.com; Jiangjiang Zhu, zhujiang4656@hotmail.com

Supplementary Figure S1

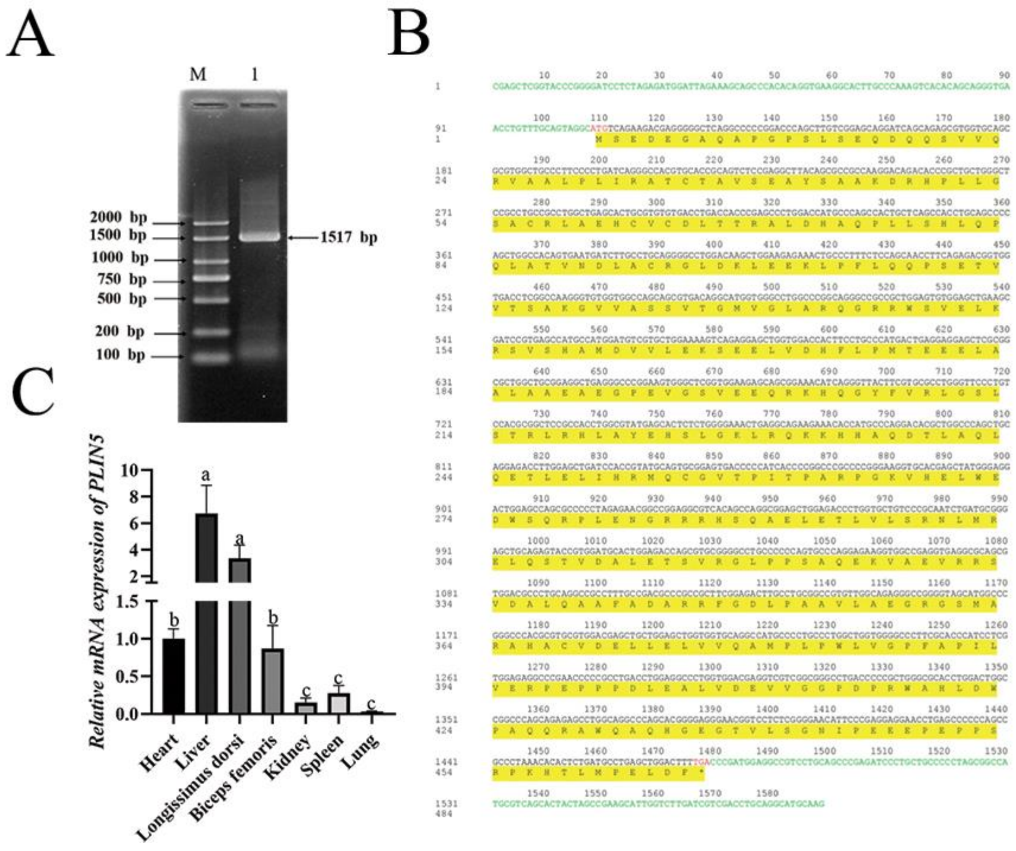

Figure S1: *Cloned a full-length cDNA for goat PLIN5.* (A) Electrophoretic analysis of the cloned goat *PLIN5* gene. Lane M: DNA Marker; Lane 1: PCR product of the *PLIN5* gene. (B) The nucleotide sequence and the translated 456 amino acid sequence of goat *PLIN5* (green indicates 5'UTR and 3'UTR, yellow indicates amino acid sequence, red indicates initiation codon and stop codon, and "*" Represents the stop codon). (C) The *PLIN5* mRNA level in heart, liver, longissimus dorsi, biceps femoris, kidney, spleen, and lung, n=3. Different lowercase letters indicate statistically

significant differences ($p < 0.05$). Different lowercase letters indicate statistically significant differences ($p < 0.05$)

Supplementary Figure S2

The theoretical molecular formula of PLIN5 was predicted by the Protparam program to be $C_{2194}H_{3520}N_{646}O_{673}S_{16}$, with a PI of 5.44 and a fat coefficient of 89.1. More detailed information can be found in Table S4. The amino acid sequences encoded by the PLIN5 gene in different species were compared using NCBI, and the similarity of the Jeju big-eared sheep with sheep (Ovis aries), cattle (Bos taurus), pigs (Sus scrofa), humans (Homo sapiens), and mice (Mus musculus) was 99.27, respectively. aries), cattle (Bos taurus), pig (Sus scrofa), human (Homo sapiens), and mouse (Mus musculus) with similarities of 99.27%, 97.45%, 89.47%, 83.92%, and 78.00%, respectively (Figure S2A). Using MEGA 5 software, the differences between the amino acid sequences of goat PLIN5 and the amino acid sequences of sheep, cattle, and mice were compared to build an evolutionary tree to determine the evolutionary relationships between species. In the evolutionary tree, there was a closer kinship between goats and antelope and sheep, and the most distant evolutionary distance with humans (Figure S2B). The transmembrane structure of the protein was analyzed using the online software TMHMM Server V 2.0 (Figure S2C), which showed that the PLIN5 protein had no transmembrane structure. The phosphorylation sites of PLIN5 protein were analyzed on the NetPhos 3.1 online website (Figure S2D), and the results showed that 31 phosphorylation sites existed. The Expasy.org website analyzed the hydrophilicity of PLIN5 amino acids (Figure S2E), and the maximum value of hydrophobicity in the protein was 4.5 (isoleucine amino acid, position 383), and the minimum value was -4.5 (arginine amino acid, position 277), and most regions showed hydrophilicity (Figure S2B). showed hydrophilicity, predicting that it might be a hydrophilic protein. Signal peptide prediction using EMBL-EBI (Figure S2F) revealed that goat PLIN5 did not have a signal peptide and was a non-secretory protein. The tertiary structure of the PLIN5 protein was predicted using SWISS-MODEL online software, and goat PLIN5 contained 68.64% α-helix, 27.63% irregularly coiled, and 1.54% β-folded (Figure S2G). Protein interaction analysis was performed by the STRING Interactive Database, and the analysis concluded that the PLIN5 protein may interact with PLIN1, PLIN2, PLIN4, PPARA, PNPLA2, PPARGC1A, and LIPE proteins (Figure S2H).

Physicochemical analysis of PLIN5 protein

Basic physical and chemical properties	Forecast result
Molecular formula	$C_{2194}H_{3520}N_{646}O_{673}S_{16}$
Ku Molecular weight	50228.97
Total number of atoms	7049
(PI) Theoretical pI	5.44
Aliphatic index	89.01
Instability index	58.56
Half-life	30
(GRAVY) Grand average of hydropathicity	-0.373
Total number of positively charged residues	66
Total number of negatively charged residues	47

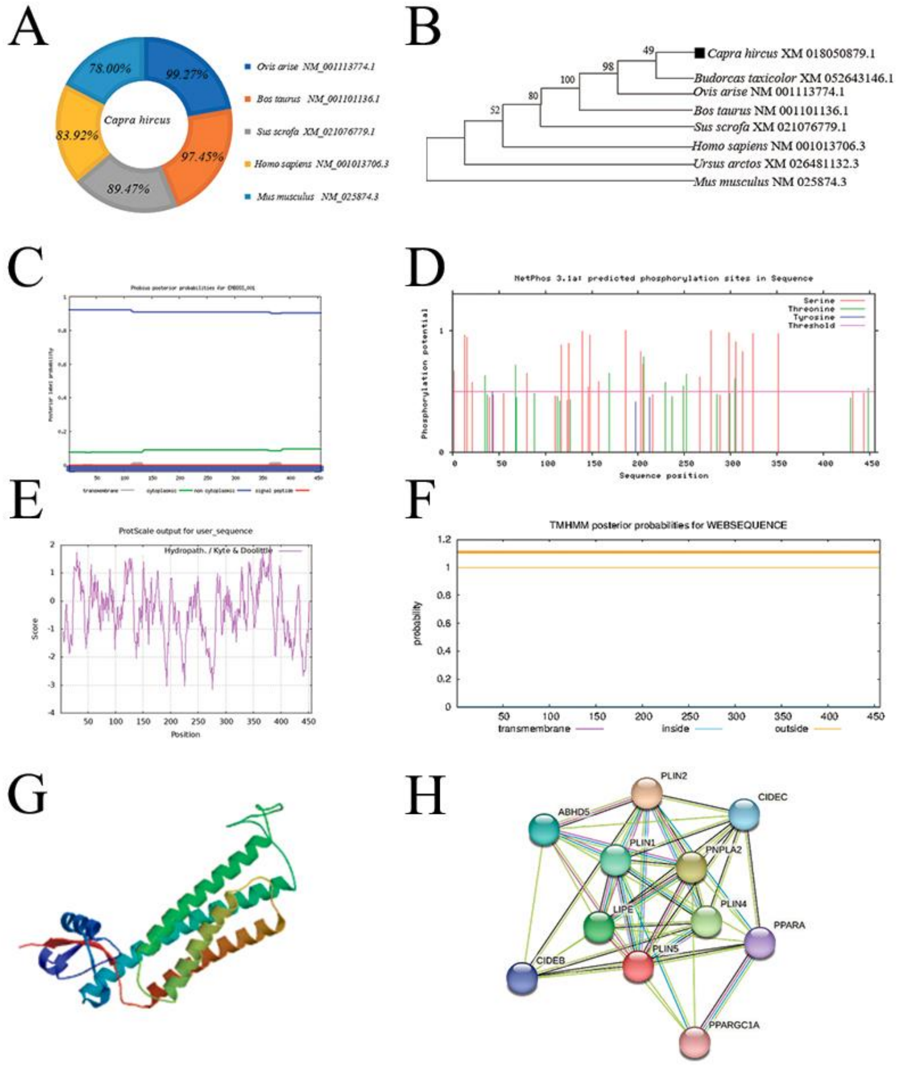

Figure S2 Characterization of a full-length cDNA for goat *PLIN5*. (A) *PLIN5* amino acid sequence phylogenetic tree was constructed by MEGA5.05. (B)Phylogenetic analysis of *PLIN5* across different species, demonstrating evolutionary relationships. (C) Signal peptide analysis of PLIN5 protein. (D) Phosphorylation site analysis of PLIN5. (E) Hydrophilicity analysis of PLIN5. (F) PLIN5 transmembrane Structural analysis. (G) Predicted tertiary structure of the PLIN5 protein. (H) Prediction of proteins interacting with goat PLIN5.

Supplementary Figure S3

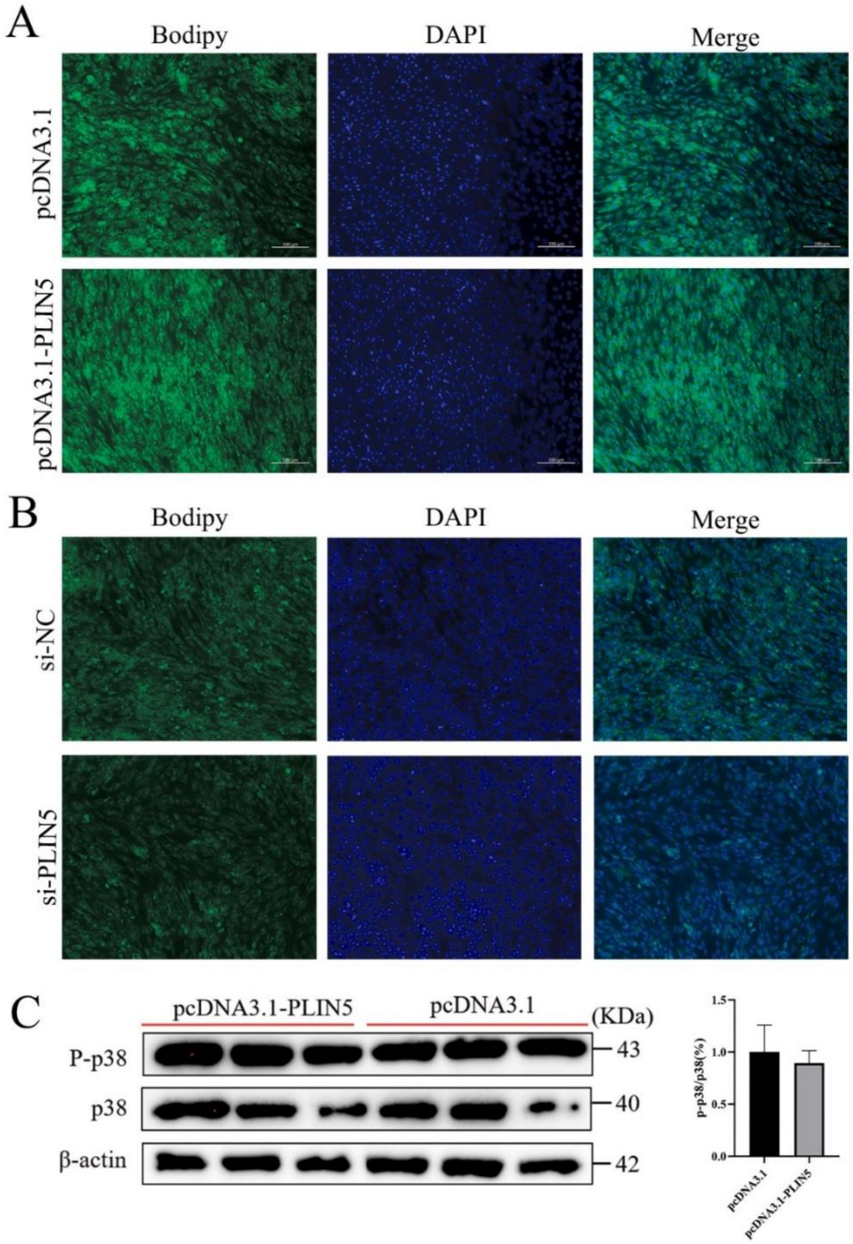

Figure S3 Bodipy staining (A) Bodipy staining after overexpression of *PLIN5*. (B) Bodipy staining after knockdown of *PLIN5*. (C) The protein expression of p-p38 and p38 in cells after transfected with pcDNA3.1-*PLIN5*.

Supplementary Figure S4

A total of 78 differentially expressed lipids were identified from 1447 lipid compounds in the positive ion mode, including 19 compounds that were up-regulated and 59 compounds that were down-regulated (Figure S4A and supplementary table S4G). The matchstick plot shows that in positive ion mode, triglycerides and diglycerides were significantly up-regulated, e.g., TG (19:0/18:1), DG (20:1/18:0), DG (20:1/16:0), DG (18:1/16:0), and TG (25:0/16:1/16:1) (Figure S4B and supplementary table 4H). The differentially expressed lipids were mainly involved in metabolism and organismal systems analyzed by KEGG classification enrichment (Figure S4C and supplementary table I).

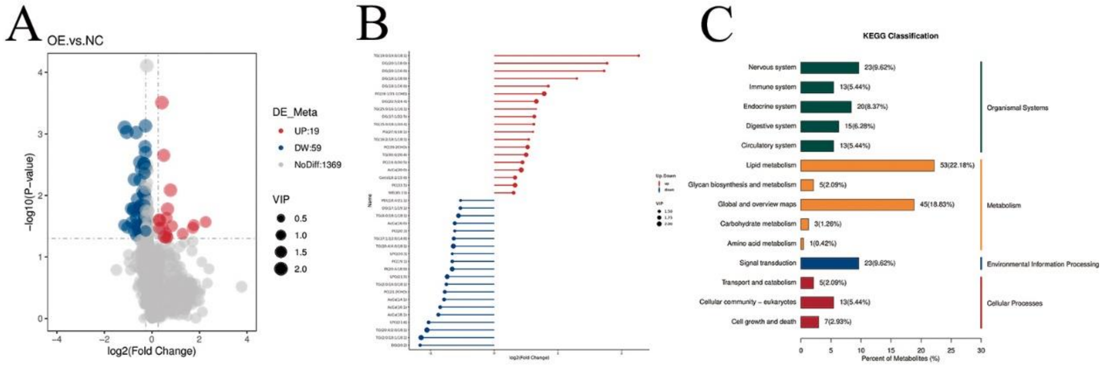

Figure S4 Identification and analysis of differential lipids after *PLIN5* overexpression. (A) Differential lipid volcano plot: significantly up-regulated lipid compounds are indicated by red dots, significantly down-regulated lipid compounds are indicated by blue dots, and the size of the dots represents the VIP value. (B) Matchstick plot of lipid compounds, with the color of the dots representing up- and down-regulation, blue representing down-regulation, and red representing up-regulation; the length of the rods representing the magnitude of log2 (fold change); and the size of the dots representing the magnitude of the VIP value. (C) KEGG classification analysis after *PLIN5* overexpression.

Supplementary Figure S5

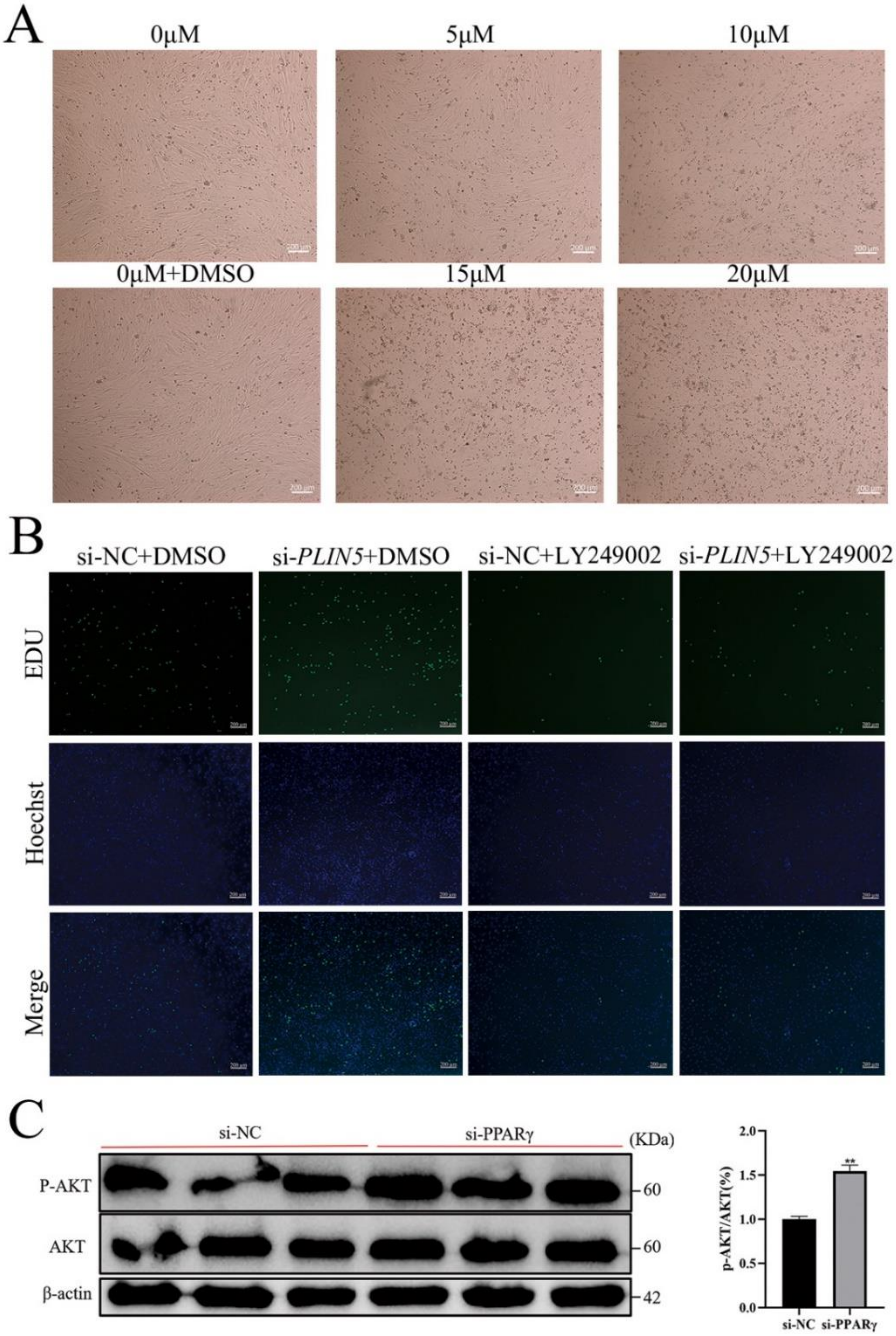

Figure S5 *PLIN5* inhibits cell proliferation by suppressing the PI3K-AKT signaling pathway (A) PI3k inhibitor (LY294002) concentration screening. The effect of PI3k inhibitor (LY294002) on intramuscular adipocytes growth at 48 h after PD169316 (0,5, 10, 15 and 20 uM) or DMSO treatment. (B) EDU staining was performed after adding LY294002 and si-*PLIN5*. (C) The expression of p-AKT / AKT after si-*PPARγ*. The data are expressed as means ± SEM. * $p < 0.05$, and ** $p < 0.01$.

Supplementary Figure S6

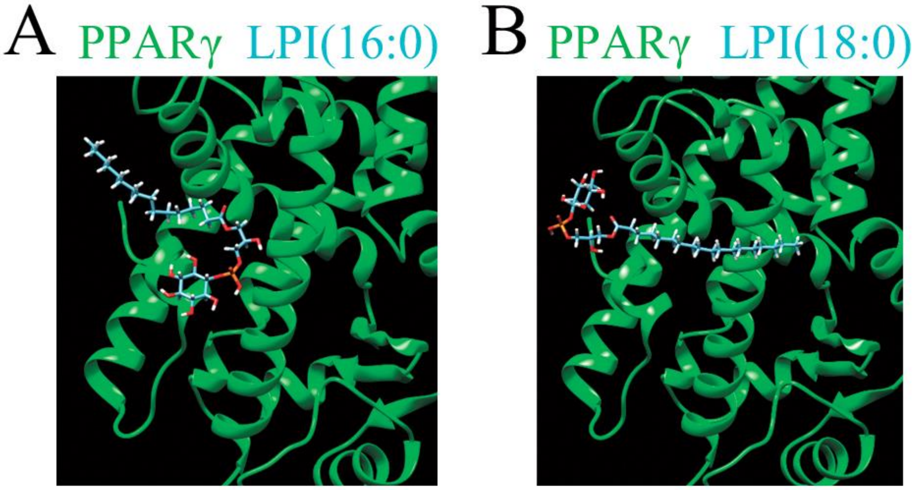

Figure S6 Molecular docking analysis (A) Molecular docking analysis of the complexes of PPARγ and LPI (16:0). (B) Molecular docking analysis of the complexes of LPI (18:0).
