# Supplementary material for: PLIN5 Promotes Lipid Reconstitution in Goat Intramuscular Fat via the PPARγ Signaling Pathway
Source: Biology (Basel). 2025 Nov 4;14(11):1547. doi: 10.3390/biology14111547 (PMC12649955; doi:10.3390/biology14111547)
Supplement: Supplementary file 1 [file biology-14-01547-s001.zip › Supplementary tables S1-S3.pdf]

Supplementary tables

PLIN5 Promotes Lipid Reconstitution in Goat Intra-muscular Fat via the PPARγ Signaling Pathway

Yuhan Dai [1], Yuling Yang[1], Haiyang Li[1], YingGui, Wang[1] Yong Wang [1,2], Yaqiu Lin [1,2], Hua Xiang[1, 2], Lian Huang[1], Zhanyu Du[1], Changhui Zhang[1] *, and Jiangjiang Zhu[1, 2*]
***Correspondence:** zhangchanghui0074@163.com; zhujiang4656@hotmail.com

Table S1. Primers for *PLIN5* cloning and subcloning

Gene	Full Name	Primer name	Sequence	Tm /℃	Application
PLIN5	Perilipin 5	Sense	CACACAGGTGAAGGCACTTG	55	PCR
		Antisense	TTCGGCTAGTAGTGCTGACG		
PLIN5	Perilipin 5	sub-Sense	CCC**AAGCTT**GCCACCATGGAGCAGAAACTCATCTCTGAAGAGCTGTCAGAAGACGAGGGGGC	55	PCR
		sub-Antisense	CCG**GAATTC**GATTACAAGGATGACGACGATAAGTCAAAAGTCCAGCTCAGGCA		

AAGCTT. *HindIII* restriction enzyme site; **GAATTC**. *EcoRI* restriction enzyme site

TableS2. Sequence analysis tools and corresponding analysis content

Analysis content	Use software tools
Primary structure analysis of amino acids	Expasy ProtParam tools
Amino acid hydrophilicity analysis	ProtScale analysis (expasy.org)
Protein phosphorylation site analysis	NetPhos 3.1 - DTU Health Tech - Bioinformatic Services

Protein signal peptide prediction	Phobius < Protein Functional Analysis < EMBL-EBI
Analysis of Protein Transmembrane	TMHMM result (dtu.dk)
Protein tertiary structure prediction	SWISS-MODEL Interactive Workspace (expasy.org)
Amino acid sequence comparison for protein tertiary structure prediction	DNAMAN
Protein interaction analysis	STRING Interactive Database
Construction of phylogenetic tree	MEGA 5.0

TableS3. Summary of genes, primers for RT-qPCR analysis.

Gene	Full Name	Primer name	Sequence	Tm/℃	Application
PLIN5	Perilipin 5	Sense	TGGAAGAGCAGCGGAAACAT	60	RT-qPCR
		Antisense	TGCATACGGTGGATCAGCTC		
ACSL1	Acyl-CoA Synthetase Long Chain Family Member 1	Sense	TGACTGTTGCTGGAGACTGG	60	RT-qPCR
		Antisense	CAGCCGTCTTTATCCAGAGC		
ACSS2	Acyl-CoA Synthetase short chain family member 2	Sense	GGCGAATGCCTCTACTGCTT	60	RT-qPCR
		Antisense	GGCCAATCTTTTCTCTAATCTGCTT		
FATP4	Fatty acid transport protein 4	Sense	CGTTCCCTACTCTGCTAC	60	RT-qPCR
		Antisense	CTCTAGATCCAGTATCCGC		
CD36	scavenger receptor B2	Sense	GTACAGATGCAGCCTCATTTCC	60	RT-qPCR
		Antisense	TGGACCTGCAAATATCAGAGGA		
FASN	de novo fatty acid synthesis gene	Sense	GGGCTCCACCACCGTGTTCCA	60	RT-qPCR
		Antisense	GCTCTGCTGGGCCTGCAGCTG		
ACC	Acetyl-CoA Carboxylase Alpha	Sense	CTCCAACCTCAACCACTACGG	60	RT-qPCR
		Antisense	GGGGAATCACAGAAGCAGCC		

SCD1	stearoyl-CoA desaturase 1	Sense	TGGCGTTCCAGAATGACGTT	60	RT-qPCR
		Antisense	ACCCCATAGATACCACGGCA		
SCD5	stearoyl-CoA desaturase 5	Sense	CTGCTCTGGGCCTATTTCTG	60	RT-qPCR
		Antisense	CCTCGACCACTCGAAGATGT		
ELOVL3	Long-chain fatty acid family member 3	Sense	CACTGTCGGTATCCTGGGCTT	60	RT-qPCR
		Antisense	CTCCAACCACTCACTGGCTC		
ELOVL6	Long-chain fatty acid family member 6	Sense	GGAAGCCTTTAGTGCTCTGGTC	60	RT-qPCR
		Antisense	ATTGTATCTCCTAGTTCGGGTGC		
DGAT1	Diacylglycerol O-Acyltransferase1	Sense	CCACTGGGACCTGAGGTGTC	60	RT-qPCR
		Antisense	GCATCACCACACACCAATTCA		
DGAT2	Diacylglycerol O-Acyltransferase2	Sense	CATGTACACATTCTGCACCGATT	60	RT-qPCR
		Antisense	TGACCTCCTGCCACCTTTCT		
HSL	Hormone-sensitive lipase	Sense	GGGAGCACTACAAACGCAACG	60	RT-qPCR
		Antisense	TGAATGATCCGCTCAAACTCG		
ATGL	Phospholipase Domain Containing 2	Sense	GGAGCTTATCCAGGCCAATG	60	RT-qPCR
		Antisense	TGCGGGCAGATGTCACTCT		
PPARα	Peroxisome proliferator-activated receptor alpha	Sense	TACTCTCGGCAGACTTCCTAC	60	RT-qPCR
		Antisense	CCTCCTCACATCTGTCATACAC		
PPARγ	Peroxisome proliferator-activated receptor gamma	Sense	AAGCGTCAGGGTTCCACTATG	60	RT-qPCR
		Antisense	GAACCTGATGGCGTTATGAGAC		
C/EBPα	Enhancer-binding	Sense	GCGGCAAAGCCAAGAAGTCC	60	RT-qPCR

	protein alpha	Antisense	CGGCTCAGTTGTTCCACCC		
SREBP1c	Sterol regulatory element-binding protein 1c	Sense	ACGCCATCGAGAAACGCTAC	60	RT-qPCR
		Antisense	GTGCGCAGACTCAGGTTCTC		
CCND2	cyclin D2	Sense	GGGCAAGTTGAAATGGAA	60	RT-qPCR
		Antisense	TCATCGACGGCGGGTAC		
CDK2	cyclin dependent kinase 2	Sense	GCCAGGAGTTACTTCTATGC	60	RT-qPCR
		Antisense	TGGAAGAAAGGGTGAGCC		
CDK4	cyclin dependent kinase 4	Sense	AAGTGGTGGGACAGTCAAGC	60	RT-qPCR
		Antisense	ACAGAAGAGAGGCTTTCGACG		
PCNA	proliferating cell nuclear antigen	Sense	ATCAGCTCAAGTGGCGTGAA	60	RT-qPCR
		Antisense	TGCCAAGGTGTCCGCATTAT		
Casepase7	Caspase7	Sense	GGAACAGATGGCAAGACAGCAATAAAG	60	RT-qPCR
		Antisense	GCCTGAATGAAGAAGAGTTTGGGTTTC		
Casepase3	Caspase3	Sense	GACGTGGATGCAGCAAACCTCA	60	RT-qPCR
		Antisense	TTCACCATGGCTTAGAAGCACG		
Bax	BCL2 associated X	Sense	TTTCCGACGGCAACTTCAA	60	RT-qPCR
		Antisense	TGAGCACTCCAGCCACAAA		
Bcl-2	cell lymphoma-2	Sense	ATGTGTGTGGAGAGCGTCAA	60	RT-qPCR
		Antisense	CCTTCAGAGACAGCCAGGAG		
UXT	Ubiquitously expressed prefoldin like chaperone	Sense	GCAAGTGGATTTGGGCTGTAAC	60	RT-qPCR
		Antisense	ATGGAGTCCTTGGTGAGGTTGT		
